# Comparative effectiveness and safety of acupuncture treatments for primary insomnia: a systematic review and network meta-analysis of randomized trial

**DOI:** 10.3389/fneur.2026.1750474

**Published:** 2026-03-03

**Authors:** Ting Fang, Xinrui Cao, Lin Liu, Shiyou Lu

**Affiliations:** 1 School of Acupuncture and Massage, Shandong University of Traditional Chinese Medicine, Jinan, Shandong, China; 2 Affiliated Hospital of Shandong University of Traditional Chinese Medicine, Jinan, Shandong, China; 3 Shandong Key Laboratory of Traditional Chinese Medicine Efficacy and Mechanism, Jinan, Shandong, China

**Keywords:** acupuncture therapy, insomnia, NMA, primary insomnia, systematic review

## Abstract

**Background:**

This study employed a Bayesian network meta-analysis (NMA) to systematically evaluate the efficacy and safety of various acupuncture therapies compared to conventional medication, sham acupuncture, and other interventions for primary insomnia.

**Methods:**

PubMed, Embase, Cochrane Library, Web of Science, CNKI, VIP Chinese Scientific Journals, Wanfang, and China Biology Medicine were searched from inception to July 16, 2025. Literature quality was assessed using the Cochrane Risk of Bias Tool v 2.0 (RoB 2.0). Statistical analyses were performed using Stata 18 and R 4.5.1.

**Results:**

In total, 80 studies involving 7,791 patients were included. Among these, 60.0% were rated as low RoB, 26.3% as unclear RoB, and 13.8% as high RoB. Statistical analysis showed that, compared with conventional medication, abdominal acupuncture (Weighted Mean Difference (MD) −3.73; 95% Credible Interval (95% CrI) [−6.88, −0.55]), acupuncture (MD −1.96; 95% CrI [−2.64, −1.27]), and catgut embedding (MD −3.08; 95% CrI [−5.18, −0.93]) significantly reduced the short-term Pittsburgh sleep quality index (PSQI) scores. Compared with acupuncture, warm acupuncture (MD −2.55; 95% CrI [−4.88, −0.21]) significantly reduced the long-term PSQI scores. Compared with sham acupuncture, abdominal acupuncture (Standardized Mean Difference (SMD) −3.06; 95% CrI [−6.08, −0.09]) and acupuncture (SMD −2; 95% CrI [−3.05, −0.98]) significantly reduced anxiety scores; meanwhile, acupuncture (SMD −1.52; 95% CrI [−2.79, −0.26]) significantly reduced depression scores. Compared with conventional medication, acupuncture (Relative Risk (RR) 1.19; 95% CrI [1.12, 1.27]) and catgut embedding (RR 1.25; 95% CrI [1.05, 1.52]) significantly improved clinical efficacy rates. However, no significant differences were observed in the relative effectiveness among different acupuncture therapies. The cumulative sample size included in the safety analysis was 1,772, from which 99 adverse events were reported (5.59%). No significant differences were detected across interventions; based on the surface under the cumulative ranking curve (SUCRA), wrist-ankle needle may show higher potential safety.

**Conclusion:**

Currently, no single intervention has emerged as optimal across all outcomes. Abdominal acupuncture, catgut embedding, electroacupuncture, and wrist-ankle needle ranked relatively high for certain outcomes based on SUCRA and showed potential advantages. However, given the potential publication bias, variations in acupuncture protocols, and insufficient long-term follow-up data, further validation is required.

**Systematic review registration:**

https://www.crd.york.ac.uk/PROSPERO/view/CRD420251040450, Identifier: CRD420251040450.

## 1 Background

Primary Insomnia (PI) represents a prevalent sleep dysfunction manifested as persistent challenges with initiating sleep, sleep maintenance problems, or early waking. It cannot be directly explained by other medical conditions, psychiatric disorders, or substance abuse (1). With the accelerating pace of contemporary existence and escalating work-related stress, the prevalence of PI has significantly increased. About 6–10% of people comply with the diagnostic criteria for PI (2, 3). Chronic insomnia severely affects patients’ daytime functioning, quality of life, and mental health. Also, it is strongly correlated with varied health issues like cardiovascular diseases and immune system disorders (4). Currently, Western medicine treatments for PI mainly focus on cognitive behavioral therapy for insomnia (CBT-I) and pharmacotherapy (5). Though benzodiazepines and non-benzodiazepine sedative-hypnotics exhibit significant short-term effects, long-term use might trigger dependency, tolerance, and side effects (6, 7). While CBT-I is suggested as the dominant treatment, its accessibility and adherence remain limited (8). Consequently, an increasing number of clinicians and patients are seeking complementary and alternative medicine treatments. As a traditional Chinese medicine (TCM) treatment, acupuncture is extensively utilized for treating PI.

Acupuncture manages the circulation of Qi and blood, balances Yin and Yang, improves sleep structure, and alleviates anxiety and depression symptoms (9). Common acupuncture therapies utilized in clinical practice include traditional acupuncture, electroacupuncture, abdominal acupuncture, catgut embedding, and press needle. Varied acupuncture methods may exert the effects through multiple targets and pathways (9). Recently, evidence has ascertained the potency and safety of various acupuncture interventions in treating PI (10–13). Xu et al. (12) is pioneering in implementing a Bayesian network meta-analysis (NMA) to contrast the efficiency of varied acupuncture treatments for PI. The study involved five acupuncture treatments (conventional acupuncture, electroacupuncture, scalp acupuncture, warm acupuncture, and combined electroacupuncture-scalp acupuncture). The primary outcome was clinical efficacy rates. It was observed that scalp acupuncture was the most efficient method. However, the encompassed studies had short treatment durations and lacked follow-up, which was insufficient to draw conclusions regarding the long-term effects of acupuncture. Subsequently, Zhao et al. (13) confirmed through a NMA that acupuncture could improve objective sleep indicators. Nevertheless, the interventions were confined to traditional acupuncture and electroacupuncture, which were relatively narrow in scope. Lu et al. (11) further compared the efficacy of various acupuncture therapies for PI through a Bayesian NMA, including conventional acupuncture, electroacupuncture, catgut embedding, auricular acupuncture, and others. They detected that catgut embedding was the best method for diminishing the Pittsburgh sleep quality index (PSQI) scores, although the risk of adverse events was not considered. Currently, the quantity of high-quality studies on acupuncture for PI is increasing, covering more acupuncture methods like press needle (14) and warm acupuncture (15). Therefore, it is necessary to implement a novel and comprehensive Bayesian NMA. We should include more studies with expanded sample sizes, a wider spectrum of outcome indicators, and various acupuncture methods to enable a more thorough evaluation.

This research implemented a Bayesian NMA involving nine acupuncture methods: conventional acupuncture, abdominal acupuncture, electroacupuncture, catgut embedding, press needle, scalp acupuncture, wrist ankle needle, warm acupuncture, and fire needle. We evaluated outcome indicators like PSQI, anxiety scores, depression scores, TCM syndrome scores, clinical efficacy rates, and adverse events, and considered both short-term and long-term effects. This comprehensive analysis provides robust evidence for clinical practice by ascertaining the relative effectiveness and ranking of varied acupuncture therapies in treating PI.

## 2 Methods

### 2.1 Design and registration

The NMA was implemented in accordance with the Preferred Reporting Items for Systematic Reviews and Meta-Analyses (PRISMA) guideline (16). The study was prospectively registered with the International Prospective Register of Systematic Reviews (PROSPERO; identifier CRD420251040450).

### 2.2 Inclusion and exclusion criteria

This study adhered impeccably to the Population, Intervention, Comparison, Outcomes, and Study (PICOS) framework for the formulation of inclusion and exclusion criteria.

Inclusion criteria were outlined below: (i) patients diagnosed with PI, regardless of age; (ii) the intervention group consisted of patients receiving any acupuncture method involving skin penetration, encompassing electroacupuncture, press needle, catgut embedding, or other traditional or modified acupuncture therapies and their related acupuncture adjunct techniques; (iii) the control group included patients receiving sham acupuncture, conventional medications, physical therapy, or any of the aforementioned acupuncture treatments. If both the intervention and control groups received basic treatment, it should be identical for both groups; (iv) studies ought to document at least one of the subsequent outcome measures: PSQI, anxiety scores (including Hamilton Anxiety Rating Scale, the Hospital Anxiety and Depression Scale, and the Self-Rating Anxiety Scale), depression scores (including the Hamilton Depression Rating Scale, the Hospital Anxiety and Depression Scale, and the Self-Rating Depression Scale), TCM syndrome score, clinical efficacy rates (overall response rate (%) = [(number of cases with complete response + marked response + response)/total number of cases] × 100%), and adverse events; (v) randomized controlled trials (RCTs) published within the past decade (these studies increased considerably during this phase and their clinical practices align with current standards, with patient demographics more closely resembling the present situation); (vi) the language was constrained to English and Chinese.

Exclusion criteria were outlined below: (i) studies with unclear intervention measures; (ii) cohort studies, review articles, case reports, descriptive studies, opinion articles, or abstracts; (iii) studies containing inaccurate or incomplete data that cannot be synthesized; (iv) studies without relevant outcome measures.

### 2.3 Search strategy

Two researchers (TF and XC) independently conducted extensive searches in PubMed, Embase, Cochrane Library, Web of Science, CNKI, VIP Chinese Scientific Journals, Wanfang, and China Biology Medicine up to April 29, 2025. The search was unrestricted by document type, date/time, or publication status. We implemented the literature search utilizing MeSH and free-text terms, encompassing acupuncture, PI, electroacupuncture, and catgut embedding. Given the importance of data timeliness, we performed an update retrieval on July 16, 2025, following the data extraction. Assessors searched and monitored the citation lists of experiments and associated systematic reviews to discern potentially suitable studies. (The search strategy is demonstrated in Appendix A).

### 2.4 Literature screening and data extraction

Two researchers (TF and XC) independently screened the procured literature based upon the inclusion and exclusion criteria. The retrieved literature was imported into EndNote 2021. After deduplication, the remaining literature was screened by reviewing the titles and abstracts. Complete-text articles were attained for those complying with the preliminary criteria. After reviewing the complete texts, the final studies were included. Any divergences were figured out through discussions or by consulting a third researcher (SL).

Two researchers (TF and XC) independently extracted data from the eligible studies. The extracted data encompassed title, first author, publication year, country, study type, sample size, age, diagnostic criteria, treatments, specific treatment design, treatment duration, follow-up time, and outcome measures. Any divergences were figured out through discussions or by consulting a third researcher (SL).

### 2.5 Quality assessment

Two researchers (TF and XC) independently estimated the risk of bias (RoB) utilizing the Cochrane RoB Tool for Randomized Trials, Version 2 (RoB 2.0) (17). Every study was appraised as low RoB, some concerns, or high RoB in the following themes: bias stemming from the randomization process, bias owing to deviations from the intended interventions, bias owing to missing outcome data, bias in the measurement of outcomes, bias in the selection of reported results, and bias related to the registered protocol. If one or more themes were rated as high RoB, the trial was rated as overall high RoB. If all themes were rated as low RoB, the trial was rated as overall low RoB. Following independent assessment, both reviewers cross-verified their evaluations. Any divergences were figured out through discussions or by consulting a third researcher (SL).

### 2.6 Data synthesis and statistical analysis

The statistical model was implemented utilizing JAGS in R (version 4.5.1) (RStudio, Boston, MA, United States). Standardized mean differences (SMD) or weighted mean differences (WMD) with their 95% credible intervals (CrI) were leveraged to estimate continuous variables to ascertain effect sizes. Relative risks (RR) and their 95% CrI were leveraged to estimate binary variables. All NMA adopted random-effects models owing to clinical heterogeneity across included trials (e.g., variations in countries, acupuncture methods, acupoints, and treatment frequencies). Four Markov chains were run for each outcome, with 50,000 iterations per chain, discarding the first 20,000 as burn-in. Convergence was estimated utilizing plots and Gelman-Rubin-Brooks statistics. The surface under the cumulative ranking curve (SUCRA) was leveraged to appraise the relative ranking of interventions for every outcome (18), where elevated values indicate superior ranking (18). Deviance Information Criterion (DIC) was utilized to contrast consistency and inconsistency models. A DIC difference <5 indicated acceptable consistency, and the consistency model was leveraged (19). Publication bias was estimated utilizing comparison-adjusted funnel plots. Network plots and comparison-adjusted funnel plots were generated in Stata (version 18.0).

## 3 Results

### 3.1 Retrieval results

**Figure 1:**
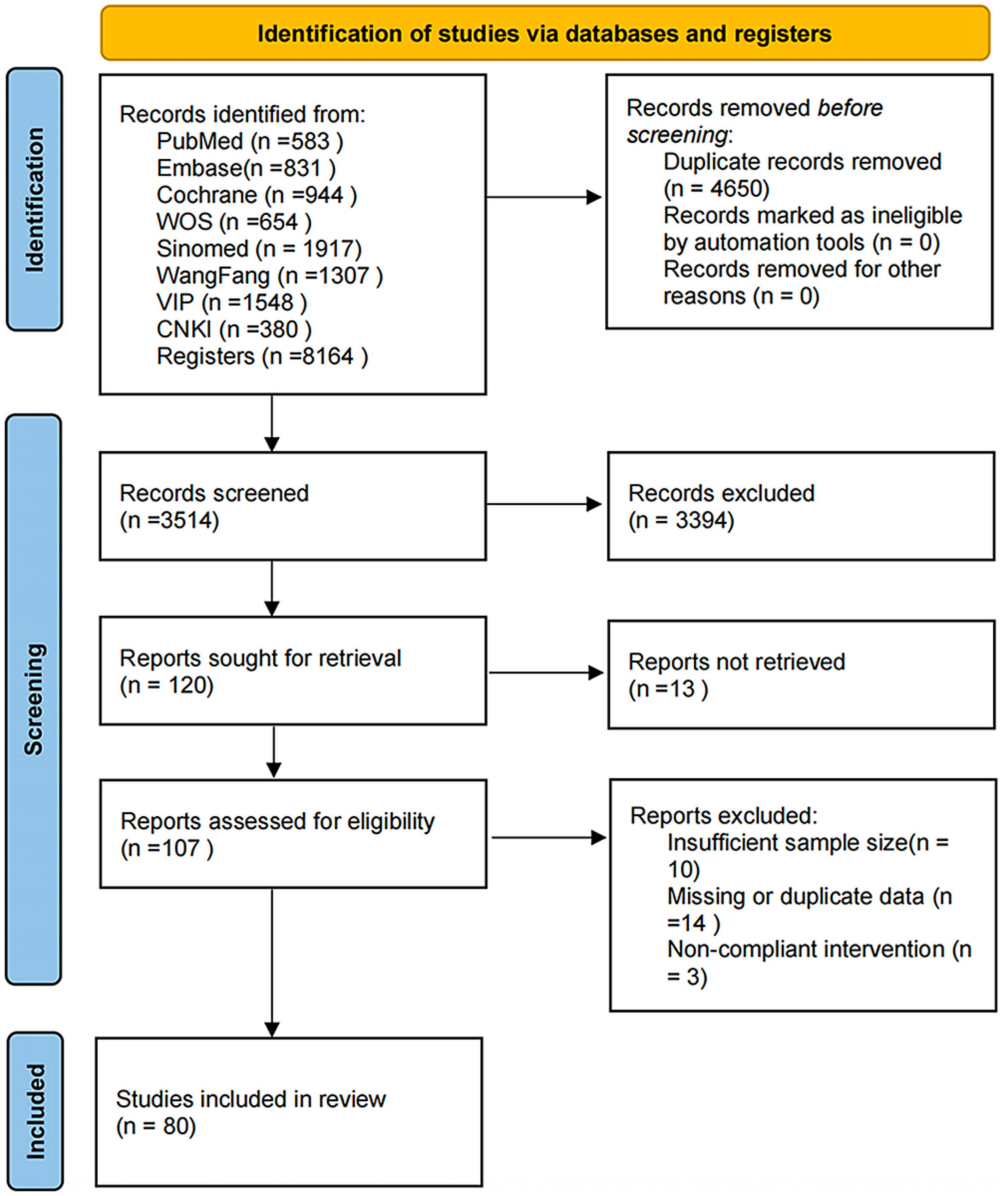
PRISMA flowchart for the retrieval and selection of eligible studies for the NMA.

The process of study selection is demonstrated in Figure 1. In total, 8,164 potentially pertinent investigations were discerned from the aforementioned 8 databases. After eliminating 4,650 duplicate studies, the titles, abstracts, and publication years of the remaining studies were screened based upon the inclusion and exclusion criteria. We solely included studies published after 2015. Subsequently, 3,394 studies were excluded. The remaining 120 studies were further appraised for qualification by scrutinizing the complete texts. Thirteen articles were excluded for inaccessible complete texts, while 27 studies were excluded owing to insufficient sample size, inappropriate interventions, or lack of data (Figure 1). Finally, 80 studies were included.

### 3.2 Features of included studies

The features of every included study are demonstrated in Tables 1 and 2. Among the 80 qualified studies published between 2015 and 2025, all were implemented in Asia (14, 15, 20–97), enrolling 7,791 patients. The sample sizes were from 60 to 510 participants, with mean age spanning 14.8–68 years. Regarding acupuncture interventions, 67 studies involved acupuncture needling (14, 20–23, 25–40, 42, 45, 48, 52, 53, 56–81, 83–97), 6 used catgut embedding (38–43), 6 employed electroacupuncture (24, 44–48), 4 utilized warm acupuncture (15, 49–51), 3 used press needle, 2 used scalp acupuncture (26, 52), 2 employed abdominal acupuncture (53, 54), 1 study used wrist ankle needle (55), and 1 study used fire needle (56). Regarding non-acupuncture interventions, 49 studies used sham acupuncture, 3 used physiotherapy, and 1 used transcranial stimulation. Regarding outcome indicators, 55 studies reported the PAQI score, 20 reported anxiety scores, 15 reported depression scores, 5 reported TCM syndrome scores, 57 reported clinical efficacy rates, and 18 reported adverse events.

**Table 1:** Principal features of included studies.

Author (year)	Nationality	Study type	Sample size (male) (*n*)			Age (years) (mean ± SD)			Intervention			Diagnostic criteria	Follow-up time	Outcomes
			Intervention						1	2	3			
			1	2	3	1	2	3						
Zhu et al. (2025) (20)	CN	SC	30 (13)	30 (12)		46.3 ± 10.8	45.6 ± 11.2		Acupuncture	Sham		ICD-10	12w	a,b,c,d,f
Jiang et al. (2024) (25)	CN	SC	30 (9)	30 (11)		36.67 ± 2.13	37.5 ± 1.8		Acupuncture	Sham		DSM-5	n.r	a,b,c
Zhang et al. (2024) (22)	CN	SC	51 (11)	53 (10)		52 ± 11.86	55.79 ± 12.72		Acupuncture	Sham		ICSD-3	n.r	a
Yu et al. (2024) (24)	CN	SC	33 (13)	34 (15)		43 ± 10	44 ± 9		Electroacupuncture	CM		CGDTI-Adults (2017)	n.r	a,d,e,f
Liu et al. (2024) (23)	CN	SC	49 (17)	48 (16)		46 ± 11	47 ± 10		Acupuncture	CM		ICSD-3	n.r	a
Geng et al. (2024) (21)	CN	SC	31 (9)	32 (11)		56.5 ± 9.8	57.1 ± 12.7		Press needle	Acupuncture		ICD-10, CCMD-3	n.r	e
Cao et al. (2024) (15)	CN	SC	30 (8)	30 (15)		66 ± 5	67 ± 4		Warm acupuncture	CM		ICSD-3, CCMD-3	4w	a,e,f
Zhang et al. (2023) (14)	CN	SC	32 (19)	32 (17)		50.24 ± 5.06	49.24 ± 5.16		Press needle	Acupuncture		ICSD-3	4w	a,e
Wu et al. (2023) (27)	CN	SC	45 (22)	45 (24)		43 ± 8	43 ± 8		Acupuncture	CM		ICSD-3	n.r	a,e,f
Ding et al. (2023) (26)	CN	SC	30 (10)	30 (8)	30 (8)	44.83 ± 14.31	43.8 ± 16.5	45.53 ± 14.4	Scalp needle	CM	Acupuncture	CGDTI-Adults (2017)	4w	a,d,e,f
Zhang et al. (2022) (30)	CN	SC	44 (16)	46 (16)		38.09 ± 13.33	39.41 ± 13.93		Acupuncture	Sham		DSM-5	6/18/42w	a,b,c,e
Lu et al. (2022) (31)	CN	SC	69 (28)	68 (29)		59.2 ± 6.1	60.1 ± 5.8		Acupuncture	Sham		DSM-5	4w	a
Yu et al. (2022) (29)	CN	SC	80 (18)	75 (21)		51.14 ± 13.47	48.41 ± 16.98		Acupuncture	CM		DSM-5, CCMD-3	4w	a,b,c,e,f
Gao et al. (2022) (28)	CN	SC	60 (35)	60 (32)		46.8	48.2		Physiotherapy	Acupuncture		CCMD-3	n.r	e
Yeung et al. (2021) (32)	CN	SC	70 (14)	70 (14)		42.2 ± 12.8	42 ± 13.3		Acupuncture	Sham		DSM-5	5w	b,c
Wang et al. (2021) (33)	CN	SC	41 (13)	41 (10)		57 ± 11.75	58 ± 12		Acupuncture	Sham		ICSD-3	4w	a
Zhang et al. (2020) (57)	CN	SC	48 (22)	48 (21)		36.6 ± 14.4	39.2 ± 13.8		Acupuncture	Sham		DSM-5	4w	a,b,c,e,f
Lee et al. (2020) (46)	Korea	SC	49 (9)	52 (9)		51.78 ± 8.86	52 ± 8.91		Electroacupuncture	Sham		DSM-5	4/8w	a,b,c,f
Wu et al. (2020) (34)	CN	SC	76 (34)	76 (33)		52.5 ± 8.6	51.5 ± 8.4		Acupuncture	CM		CGDTI-Adults (2012)	12w	e
Zhang et al. (2020) (44)	CN	SC	32 (14)	32 (13)		44.21 ± 10.8	43.52 ± 11.51		Physiotherapy	Electroacupuncture		CCMD-3	4w	a,e
Liang et al. (2020) (36)	CN	SC	30 (8)	30 (15)		56.07 ± 10.46	60.1 ± 11.22		Acupuncture	CM		Individualized clinical practice guidelines for insomnia in TCM.	4w	a,b
Wu et al. (2020) (45)	CN	SC	35 (16)	34 (14)		42 ± 10	43 ± 10		Electroacupuncture	Acupuncture		DSM-5	8w	a,b,c,e
Sun et al. (2020) (53)	CN	SC	30 (12)	30 (14)		36 ± 12	39 ± 11		Abdominal needle	Acupuncture		CCMD-3	n.r	b,e
Li et al. (2020) (38)	CN	SC	34 (8)	34 (10)		37 ± 12	41 ± 11		Catgut embedding	Acupuncture		Diagnosis and Treatment Guidelines for Common Diseases in Internal Medicine of TCM-Section on TCM Diseases and Syndromes.	n.r	a,b,c,d,e,f
Chen et al. (2020) (37)	CN	SC	43 (20)	42 (19)		52.33 ± 10	52.43 ± 9.67		Acupuncture	CM		CCMD-3	2w	a,e
Zou et al. (2020) (35)	CN	SC	30 (19)	30 (17)		54.32 ± 6.09	54.15 ± 6.26		Acupuncture	CM		CCMD	n.r	a,e
Zhao et al. (2019) (58)	CN	SC	32 (13)	32 (15)		36.94 ± 10.22	39.31 ± 10.88		Acupuncture	Sham		ICSD-3, DSM-I4	n.r	a,f
Xu et al. (2019) (39)	CN	SC	170	170	170	n.r			Catgut embedding	Acupuncture	CM	CCMD-3	n.r	f
Guan et al. (2019) (59)	CN	SC	40 (18)	39 (19)		34.3 ± 6.6	33.2 ± 6.3		Acupuncture	CM		ICSD-3	n.r	a,e
Zhu et al. (2019) (60)	CN	SC	30 (16)	30 (12)		42.08 ± 13.11	44.51 ± 11.47		Acupuncture	CM		CCMD-3	n.r	a,e
Li et al. (2019) (55)	CN	SC	75 (44)	75 (47)		41.43 ± 4.4	41.7 ± 5.31		Wrist ankle needle	CM		CCMD-3	n.r	e,f
Zhang et al. (2019) (61)	CN	SC	30 (17)	30 (12)		42.9 ± 10.03	42.56 ± 10.58		Acupuncture	CM		ICD-10	n.r	a,e
Zhang et al. (2019) (62)	CN	SC	32 (7)	32 (12)		39 ± 11.7	41 ± 13.5		Acupuncture	Sham		ICSD-3	8w	a
Yuan et al. (2019) (63)	CN	SC	40 (33)	42 (36)		47.41 ± 5.84	48.13 ± 4.31		Acupuncture	Sham		DSM-4	n.r	a,f
Wang et al. (2019) (64)	CN	SC	60 (31)	60 (28)		42 ± 11	41 ± 11		Acupuncture	CM		CGDTI-Adults (2012)	n.r	a,e
Guo et al. (2019) (65)	CN	SC	60 (11)	60 (15)		46.17 ± 3.71	48.67 ± 3.14		Acupuncture	Sham		DSM-5	8w	a,f
Li et al. (2019) (54)	CN	SC	30	30		n.r	n.r		Abdominal needle	CM		DSM-4	4/12w	a
Qi et al. (2019) (66)	CN	SC	60 (34)	60 (32)		47.86 ± 6.73	47.95 ± 6.82		Acupuncture	CM		n.r	n.r	a,b
Zhao et al. (2018) (67)	CN	MC	30 (12)	30 (14)		36.8 ± 10.7	38.4 ± 10.8		Acupuncture	Sham		ICSD-3, DSM-5, CCMD-3	n.r	a,f
Zhuo et al. (2018) (68)	CN	SC	56 (34)	56 (33)		42.32 ± 14.25	41.62 ± 13.61		Acupuncture	CM		DSM-4	n.r	a,b,c,e
Wan et al. (2018) (47)	CN	MC	36 (16)	36 (19)		38.72 ± 12.99	39.64 ± 12.59		Transcranial stimulation	Electroacupuncture		CCMD-3	n.r	e
Dong et al. (2018) (69)	CN	SC	36 (24)	36 (11)		45 ± 18	44 ± 21		Acupuncture	Sham		CGDTI-Adults (2012)	4w	a,e
Xie et al. (2018) (70)	CN	SC	45 (25)	45 (23)		48 ± 7	46 ± 8		Acupuncture	CM		n.r	n.r	b,e
Zhang et al. (2018) (71)	CN	MC	33 (15)	34 (17)		31.36 ± 13.15	35.62 ± 10.25		Acupuncture	Sham		CCMD-3	4/12w	a,b
Cai et al. (2018) (72)	CN	SC	93 (48)	93 (47)		45 ± 4	46 ± 5		Acupuncture	CM		CCMD-3	n.r	a,b,e
Hao et al. (2018) (73)	CN	SC	45 (24)	45 (23)		52.3 ± 9.75	53.1 ± 9.5		Acupuncture	CM		n.r	n.r	a,e
Kan et al. (2018) (49)	CN	SC	35 (16)	35 (17)		49.74 ± 8.64	48.63 ± 8.44		Warm acupuncture	CM		CCMD-3	n.r	a,e
Zhou et al. (2018) (74)	CN	SC	30 (12)	30 (14)		49.1 ± 16.7	48.7 ± 15.4		Acupuncture	CM		ICD-10, CCMD-3	n.r	a,e
Guo et al. (2018) (75)	CN	SC	30 (10)	31 (13)		52.2 ± 13.21	55.94 ± 13.47		Acupuncture	CM		CCMD-3	n.r	b,c
Xuan et al. (2017) (76)	CN	SC	36 (17)	36 (15)		16.4 ± 3.3	14.8 ± 3.9		Acupuncture	Sham		DSM-IV	4w	b,c,f
Zhao et al. (2017) (77)	CN	SC	106 (23)			42.98 ± 14.19			Acupuncture	CM		CCMD-3	n.r	e,f
Wang et al. (2017) (41)	CN	SC	30 (10)	30 (9)		46.98 ± 15.13	47.25 + 16.29		Catgut embedding	CM		CCMD	n.r	d
Han et al. (2017) (78)	CN	SC	68 (28)	65 (30)		48.2 ± 9.6	46.2 ± 8.6		Acupuncture	CM		CCMD-3	n.r	a,e
Xie et al. (2017) (79)	CN	SC	30 (14)	30 (13)		45.23 ± 5.12	46.15 ± 4.74		Acupuncture	CM		CCMD-3	4w	a,e
Shao et al. (2017) (80)	CN	SC	56 (20)	56 (22)		44.6 ± 13.5	45.8 ± 14.1		Acupuncture	CM		CCMD-3	n.r	a,e
Hong et al. (2017) (52)	CN	SC	30 (9)	30 (11)		50 ± 11	54 ± 13		Scalp needle	Acupuncture		ICD-10	n.r	e
Sun et al. (2017) (56)	CN	MC	136 (49)	128 (46)		41.6 ± 10.3	40.8 ± 11.1		Fire needle	Acupuncture		CCMD-3	n.r	a,e
Liu et al. (2017) (81)	CN	SC	30 (10)	31 (10)		47.52 ± 10.48	47.8 ± 10.36		Acupuncture	CM		DSM-5	n.r	a
Liang et al. (2017) (82)	CN	SC	35 (14)	35 (16)		68 ± 6	67 ± 7		Press needle	CM		CCMD-3	n.r	a,e
Zhang et al. (2017) (40)	CN	SC	40 (16)	39 (18)	38 (17)	41.75 ± 11.1	40.18 ± 10.17	42.28 ± 10.31	Catgut embedding	Acupuncture	CM	CCMD-2	4w	a,e
Wang et al. (2016) (48)	CN	SC	88 (34)			16–68			Electroacupuncture	Acupuncture		CCMD-3	12w	e
Bo et al. (2016) (51)	CN	SC	40 (24)	40 (22)		43.25 ± 9.56	47.21 ± 8.31		Warm acupuncture	CM		ICSD-2	n.r	a,e
Li et al. (2016) (83)	CN	SC	35 (15)	35 (14)		42 ± 3	44 ± 4		Physiotherapy	Acupuncture		CCMD-3	2w	e
Hua et al. (2016) (84)	CN	SC	45 (22)	45 (23)		29.6 ± 4.3	28.9 ± 5		Acupuncture	CM		CCMD-3	n.r	e
Gou et al. (2016) (85)	CN	SC	32 (6)	30 (6)		40 ± 16	37 ± 14		Acupuncture	Sham		DSM-4	4w	e
Wang et al. (2016) (86)	CN	MC	34 (9)	34 (11)		53 ± 13.43	53 + 11.37		Acupuncture	CM		CCMD-3	n.r	a,e
Wang et al. (2016) (50)	CN	SC	32 (18)	30 (17)		46.72 ± 9.16	47.6 ± 9.09		Warm acupuncture	CM		CCMD-3	4w	a,e
Luo et al. (2016) (87)	CN	MC	31	30		40.17 ± 13.53			Acupuncture	CM		CCMD-2	n.r	b,c,e
Wang et al. (2016) (88)	CN	SC	64			n.r			Acupuncture	CM		CCMD-3	n.r	a,e
Zhang et al. (2015) (89)	CN	SC	38 (18)	37 (20)		42 ± 12	41 ± 11		Acupuncture	CM		n.r	n.r	e
Ji et al. (2015) (90)	CN	SC	94 (41)	93 (39)		46 ± 7	45 ± 6		Acupuncture	CM		CCMD-3	n.r	e
Ding et al. (2015) (43)	CN	SC	33 (14)	31 (16)		44 ± 15	41 ± 12		Catgut embedding	CM		ICD-10, CCMD-3	n.r	a,e
Zou et al. (2015) (91)	CN	SC	60 (24)	40 (12)		17 ~ 77	20 ~ 74		Acupuncture	CM		CCMD-3	n.r	e
Liu et al. (2015) (92)	CN	SC	96	95		21–70	23–68		Acupuncture	CM		ICD-10, CCMD-3	n.r	e,f
Liu et al. (2015) (42)	CN	SC	40 (21)	40 (22)		50 ± 8	50 ± 9		Catgut embedding	Acupuncture		CCMD-3	n.r	a,e
Hong et al. (2015) (93)	CN	SC	150	150		n.r	n.r		Acupuncture	CM		Diagnostic criteria for circadian sleep disorders established by the American Sleep Disorders Association in 2005	n.r	e
Wang et al. (2015) (94)	CN	SC	60 (28)			38.83 ± 7.04			Acupuncture	CM		CCMD-3, ICD-10	n.r	b,c,e
Zhang et al. (2015) (95)	CN	SC	62 (24)	57 (23)		44.98 ± 11.67	43.18 ± 12.08		Acupuncture	CM		Diagnostic Criteria for Internal Diseases	n.r	a
Liu et al. (2015) (96)	CN	SC	31	31		44.71 ± 3.04	44.32 ± 3		Acupuncture	CM		CCMD-3	n.r	a,e
Ji et al. (2015) (97)	CN	SC	35 (17)	35 (19)		37 ± 11	36 ± 13		Acupuncture	CM		ICD-10	n.r	a,c,e

CCMD, Classification and Diagnostic Criteria of Mental Disorders in China; CGDTI-Adults, Chinese Guidelines for the Diagnosis and Treatment of Insomnia in Adults; CN, China; CM, conventional medicine; DSM, The Diagnostic and Statistical Manual of Mental Disorders; ICD, International Classification of diseases; ICSD, International Classification of Sleep Disorders; MC: multi-center; SC: single-center; a, Pittsburgh Sleep Quality Index; b, Anxiety Score; c, Depression Score; d, TCM Syndrome Score; e, clinical effective rate; f, adverse event; n.r, not report.

**Table 2:** Acupuncture points, retention time, and treatment frequency.

Author (year)	Acupoint		Needle retention time/treatment frequency
	Intervention 1	Intervention 2	
Zhu et al. (2025) (20)	GV20, EX-HN1, GV24, CV12, ST25, PC6, HT7, SP6, ST36	Sham	3 times/wk × 4 wks
Jiang et al. (2024) (25)	GV20, GV24, EX-HN1, HT7, GB13, PC6, SP6	Sham	30 min, 3 times/wk × 4 wks
Zhang et al. (2024) (22)	BL15, BL20, GV20, HT7, KI6, BL62, EX-HN17, SP6, ST36	Sham	14 sessions every other day over 4 wks
Yu et al. (2024) (24)	GV20, EX-HN1, GB20, Gongxue, LR3, SP6	CM	30 min,6 times/wk × 4 wks
Liu et al. (2024) (23)	GV20, HT7, SP6, BL62, KI6, BL15, BL23	CM	5 times/wk × 4 wks
Geng et al. (2024) (21)	Ear Shenmen, CO10, Ear Stomach (CO4), CO15	GV20, HT7, SP6, ST36, LR3	Press needle: 4 h; AP: 30 min; 6 times/wk × 2 wks
Cao et al. (2024) (15)	Dinghui, Heyi, Xin	CM	20 min, 3 times/wk × 3 wks
Zhang et al. (2023) (14)	HT7, EX-HN17, BL62, KI6, AT4, Ear Shenmen (TF4), CO15, CO13	GV20, HT7, SP6, EX-HN17, BL62, KI6, BL15, BL20	Press needle: 3 d, 2 times/wk × 4 wks; AP: 30 min, 6 times/wk × 4 wks
Wu et al. (2023) (27)	EX-HN1, HT7, SP6	CM	6 times/wk × 4 wks
Ding et al. (2023) (26)	MS1, MS2, MS2	A:CM	Scalp needle: 30 min, QD for 20 days AP: 30 min, QD for 20 days
		B: BL62, EX-HN17, SP6, KI6, GV20, HT7	
Zhang et al. (2022) (30)	EX-HN17, PC6, HT7, LI4, ST36, KI6, BL62, LR3	Sham	30 min, 5 times/wk × 2 wks
Lu et al. (2022) (31)	GV20, GV24, EX-HN3, EX-HN17, HT7, SP6	Sham	30 min, 3 times/wk × 4 wks
Yu et al. (2022) (29)	GV24 to GV20, CV6 to CV4	CM	30 min, 5 times/wk × 4 wks
Gao et al. (2022) (28)	Physiotherapy	EX-HN3, GV20, GV16, Ermen (TE21)	30 min, QD for 2 wks
Yeung et al. (2021) (32)	Ear Shenmen, Touwei (ST8), EX-HN1, EX-HN3, GV20	Sham	30 min, 2 times/wk × 4 wks
Wang et al. (2021) (33)	HT7, Fuliu (KI7)	Sham	30 min, 3 times/wk, 10 sessions total
Zhang et al. (2020) (57)	EX-HN17, PC6, HT7, LI4, ST36, KI6, BL62, LR3	Sham	30 min, 5 times/wk × 2 wks
Lee et al. (2020) (46)	GV20, EX-HN3, HT7, PC6, Jinmen (BL63), Dazhong (KI4)	Sham	30 min, 2–3 times/wk × 4 wks, 10 sessions total
Wu et al. (2020) (34)	GV24 to GV20, CV4 to CV6	CM	5 times/wk × 4 wks
Zhang et al. (2020) (44)	Baihui Bazhen, Fengfu Bazhen, Shendao Bazhen, Heche Lu	KI6, BL62, HT7, SP6, EX-HN17, EX-HN1	30 min, 5 times/wk × 4 wks
Liang et al. (2020) (36)	ST36, PC6, CV12	CM	30 min, QD for 4 wks
Wu et al. (2020) (45)	GB20, Gongxue, EX-HN5, EX-HN1	BL62, KI6, HT7, SP6, EX-HN17, EX-HN1	30 min, 6 times/wk × 4 wks
Sun et al. (2020) (53)	1. Three points below xiphoid process: midline: 0.5 cun below xiphoid (CV 15↓0.5); lateral: 0.5 cun bilateral to midline point 2. Four points around umbilicus: Superior/inferior: 0.5 cun above/below umbilicus (CV 8 ± 0.5); left/right: 0.5 cun bilateral to umbilicus	EX-HN1, EX-HN17, HT7, SP6, KI6, BL62	30 min, QD for 20 days
Li et al. (2020) (38)	GV20, HT7, PC6, LR3, BL18, BL15	GV20, HT7, PC6, LR3, BL18, BL15	Catgut embedding: once a week for 6 wks AP: 3 times/wk × 6 wks
Chen et al. (2020) (37)	CV14, ST25, CV6, CV4, GV24, HT7	CM	25 min, 20 treatments in cycles of 10 days on, 1 day off
Zou et al. (2020) (35)	EX-HN1, GV20	CM	5 times/wk × 4 wks
Zhao et al. (2019) (58)	GV24, GB13, EX-HN1, GV11, HT7	Sham	30 min, 3 times/wk × 8 wks
Xu et al. (2019) (39)	PC6, ST36, SP6	A: PC6, ST36, SP6	Catgut embedding: once every 10 days for a total of 3 treatments AP: QD excluding weekends, for a total of 30 days
		B:CM	
Guan et al. (2019) (59)	CV12, CV10, CV6, CV4, GV20, EX-HN1, GV24, EX-HN3	CM	30 min, 3 times/wk × 4 wks
Zhu et al. (2019) (60)	GV20, EX-HN3, GV26, Chengjiang (CV24), CV17, EX-HN5, PC6, HT7, Shaofu (HT8), ST36, SP6, KI3, Gongsun (SP4)	CM	40 min, 5 times/wk × 4 wks
Li et al. (2019) (55)	At two finger-widths proximal to the wrist crease, in the depression between the ulnar border of the ulna and the flexor carpi ulnaris muscle.	CM	5 h, 6 times/wk × 3 wks
Zhang et al. (2019) (61)	GV20, PC6, HT7, EX-HN1	CM	6 times/wk × 4 wks
Zhang et al. (2019) (62)	Shangwan (CV13), CV12, CV10, CV6, ST36, ST25, PC6	Sham	30 min, 3 times/wk × 4 wks
Yuan et al. (2019) (63)	GV20, GV24, EX-HN1, GB13, HT7, PC6, SP6	Sham	30 min, every other day over 4 wks
Wang et al. (2019) (64)	GV20, SJ5, PC6, Weishu (BL21), CV12, KI3	CM	30 min, QD for 4 wks
Guo et al. (2019) (65)	GV20, GV24, EX-HN1, GB13, HT7, PC6, SP6	Sham	30 min, 3 times/wk × 4 wks
Li et al. (2019) (54)	CV12, CV10, CV6, CV4	CM	30 min, 6 times/wk × 4 wks
Qi et al. (2019) (66)	CV17, CV12, CV6, Xuehai (SP10), ST36, SJ5	CM	30 min, frequency: QD; course Duration: 10 consecutive days; courses: 3 total; inter-course interval: 2–3 days
Zhao et al. (2018) (67)	EX-HN1, GV24, GV20, GB13, KI3, HT7	Sham	30 min, 3 times/wk × 8 wks
Zhuo et al. (2018) (68)	Qianding (GV21), Houding (GV19), Yintang +0.5 cun, Yangbai +0.5 cun, HT7, PC6, SP6	CM	6 times/wk × 8 wks
Wan et al. (2018) (47)	EX-HN3, EX-HN17	EX-HN3, EX-HN17	30 min, QD for 15 days
Dong et al. (2018) (69)	EX-HN3, GV20, GV14	Sham	30 min, 3 times/wk × 4 wks
Xie et al. (2018) (70)	GB15, Tongtian (BL7), GV24	CM	1 h, QD for 30 days
Zhang et al. (2018) (71)	HT7, EX-HN17, KI6, BL62, PC6, LI4, LR3	Sham	30 min, 10 treatments in cycles of 5 days on, 2 days off
Cai et al. (2018) (72)	GV26, EX-HN3, GV20, EX-HN1, PC6, HT7, LI4, LR3	CM	30 min,6 times/wk × 6 wks
Hao et al. (2018) (73)	SP6, HT7, PC6	CM	30 min, QD for 4 wks
Kan et al. (2018) (49)	GV20, EX-HN1, BL15, BL20, PC6, SP6, HT7	CM	30 min, QD for 6 wks
Zhou et al. (2018) (74)	Shangxing (GV23), EX-HN3, GV20, EX-HN1	CM	30 min, QD for 4 wks
Guo et al. (2018) (75)	KI6, BL62, HT7, EX-HN3, EX-HN1, EX-HN17	CM	30 min, 10 treatments in cycles of 5 days on, 2 days off
Xuan et al. (2017) (76)	GV20, GV24, EX-HN3, EX-HN17, HT7, SP6	Sham	30 min,3 times/wk × 4 wks
Zhao et al. (2017) (77)	Zhenjing, Sanchasan, Huozhu	CM	30 min, QD for 30 days
Wang et al. (2017) (41)	EX-HN1, PC6, HT7, SP6	CM	Once every 10 days for a total of 3 treatments
Han et al. (2017) (78)	GV20, EX-HN1	CM	6 times/wk × 4 wks
Xie et al. (2017) (79)	GV24, GV20, GV16, GV14, GV11, Mingmen (GV4)	CM	QD for 2 wks
Shao et al. (2017) (80)	GV20, EX-HN1, EX-HN17	CM	30 min, 30 treatments in cycles of 10 days on, 1 day off
Hong et al. (2017) (52)	GV20, GB15, Shuaigu (GB8), EX-HN3	EX-HN17, HT7, KI6, BL62	30 min,3 times/wk × 8 wks
Sun et al. (2017) (56)	ST45, SP1, EX-HN17, HT7, PC6, BL15, BL20	ST45, SP1, EX-HN17, HT7, PC6, BL15, BL20	Fire needle: no retention AP
Liu et al. (2017) (81)	EX-HN1, EX-HN17, HT7, SP6, KI6, BL62	CM	30 min, 5 times/wk × 4 wks
Liang et al. (2017) (82)	Ear Shenmen, Ear Sympathetic (Auricular Helix 6a), AT4, CO15, CO13, CO10, Ear Anterior Lobe (AT3)	CM	every other day over 30 days
Zhang et al. (2017) (40)	BL15, BL18, BL20, BL23, ST36, SP6, PC6, CV6, CV4	A: BL15, BL18, BL20, BL23, ST36, SP6, PC6, CV6, CV4	Catgut embedding: once every 10 days for a total of 3 treatments AP: 30 min, frequency: QD; course duration: 5 consecutive days; courses: 30 days; inter-course Interval: 2 days
		B:CM	
Zhang et al. (2017) (40)	EX-HN17, GV20, EX-HN1, GV24, HT7	EX-HN17, GV20, EX-HN1, GV24, HT7	30 min,5 times/wk × 3 wks
Bo et al. (2016) (51)	Dinghui, Heyi, Xin	CM	30 min, frequency: 4 times daily; course duration: 9 days; intervals: a 3-day interval between courses; total courses: 6.
Li et al. (2016) (83)	Physiotherapy	SP6, HT7, EX-HN1	20–30 min, 5 times/wk × 2 wks
Hua et al. (2016) (84)	BL18, BL15, BL20, BL13, BL23, Qimen (LR14), BL1, KI1	CM	30 min, 4 times/wk × 2 wks
Gou et al. (2016) (85)	GV20, EX-HN3, GV24, HT7, EX-HN17, SP6	Sham	30 min, 3 times/wk × 4 wks
Wang et al. (2016) (86)	GV20, GV24, EX-HN1, EX-HN17, HT7, LR3, KI3, CV12, ST25, SP9	CM	15–40 min, 5 times/wk × 4wks
Wang et al. (2016) (50)	GV11	CM	Warm AP: 5 times/wk × 2 wks
Luo et al. (2016) (87)	HT7, Zhigou (SJ6), ST36, SP6, GV20, EX-HN1, Qineihuanxue	CM	30 min, 6 times/wk × 2 wks
Wang et al. (2016) (88)	HT7, KI3, Laogong (Pericardium Meridian 8), Shuiquan (KI5)	CM	30 min, 7 times/wk × 8 wks
Zhang et al. (2015) (89)	GV20, HT7, SP6	CM	1 h, QD for 20 days
Ji et al. (2015) (90)	GV20, EX-HN3, HT7, LI4, LR3	CM	30 min, QD for 20 days
Ding et al. (2015) (43)	GV20, GV24, GB20	CM	Once a week for 4 weeks
Zou et al. (2015) (91)	BL62, Fuyang (BL59), EX-HN17, EX-HN1	CM	30 min, 5 times/wk × 4 wks
Liu et al. (2015) (92)	EX-HN1, EX-HN17, SP6, HT7, KI3, ST36, BL62	CM	1–2 h, 20 treatments in cycles of 10 days on, 2 days off
Liu et al. (2015) (42)	Tender points along the GV meridian	HT7, PC6, SP6, KI3, ST36, SP9, BL62, KI6, EX-HN1	Catgut embedding: once every 2 wks for 12 wks AP: 30 min, 6 times/wk × 12 wks
Hong et al. (2015) (93)	SP6, HT7, EX-HN1	CM	30 min, QD for 7 days
Wang et al. (2015) (94)	GV20, EX-HN1, EX-HN17, BL18, Geshu (BL17), LR3	CM	30 min, 6 times/wk × 4 wks
Zhang et al. (2015) (95)	BL1, KI1, BL15, BL20, BL13, BL23	CM	30 min, QD for 30 days
Liu et al. (2015) (96)	CV14, BL15, Zhangmen (LR13), BL20	CM	QD for 4 wks
Ji et al. (2015) (97)	HT7, LI4, LR3, GV20, EX-HN3	CM	20 min, 28 treatments in cycles of 14 days on, 1 day off

AP, acupuncture; AT4, Ear Subcortex (Auricular Therapy Point 4); BL1, Jingming (Bladder Meridian 1); BL13, Feishu (Bladder Meridian 13); BL15, Xinshu (Bladder Meridian 15); BL18, Ganshu (Bladder Meridian 18); BL20, Pishu (Bladder Meridian 20); BL23, Shenshu (Bladder Meridian 23); BL62, Shenmai (Bladder Meridian 62); CM, Conventional medication; CO10, Ear Kidney (Concha 10); CO13, Ear Spleen (Concha 13); CO15, Ear Heart (Concha 15); CV4, Guanyuan (Conception Vessel 4); CV6, Qihai (Conception Vessel 6); CV10, Xiawan (Conception Vessel 10); CV12, Zhongwan (Conception Vessel 12); CV14, Juque (Conception Vessel 14); CV17, Danzhong (Conception Vessel 17); EX-HN1, Sishencong (Extra Point-Head and Neck 1); EX-HN3, Yintang (Extra Point-Head and Neck 3); EX-HN5, Taiyang (Extra Point-Head and Neck 5); EX-HN17, Anmian (Extra Point-Head and Neck 17); GB13, Benshen (Gallbladder Meridian 13); GB15, Toulinqi (Gallbladder Meridian 15); GB20, Fengchi (Gallbladder Meridian 20); GV11, Shendao (Governor Vessel 11); GV14, Dazhui (Governor Vessel 14); GV16, Fengfu (Governor Vessel 16); GV20, Baihui (Governor Vessel 20); GV24, Shenting (Governor Vessel 24); GV26, Shuigou (Governor Vessel 26); HT7, Shenmen (Heart Meridian 7); KI1, Yongquan (Kidney Meridian 1); KI3, Taixi (Kidney Meridian 3); KI6, Zhaohai (Kidney Meridian 6); LI4, Hegu (Large Intestine Meridian 4); LR3, Taichong (Liver Meridian 3); PC6, Neiguan (Pericardium Meridian 6); QD, Once daily; SJ5, Waiguan (Sanjiao Meridian 5); SP1, Yinbai (Spleen Meridian 1); SP6, Sanyinjiao (Spleen Meridian 6); SP9, Yinlingquan (Spleen Meridian 9); ST25, Tianshu (Stomach Meridian 25); ST36, Zusanli (Stomach Meridian 36); ST45, Lidui (Stomach Meridian 45); wk, week.

### 3.3 Quality appraisal

**Figure 2:**
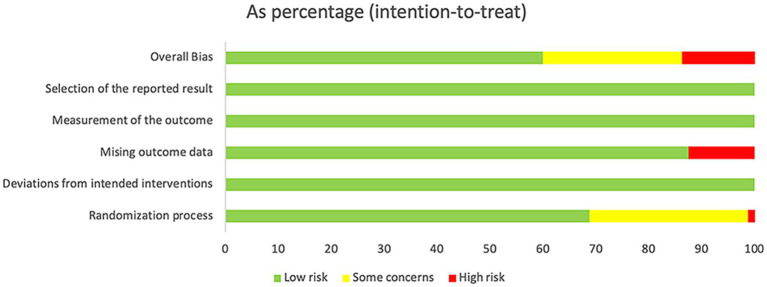
Risk of bias assessment results.

The RoB appraisal results are demonstrated in Figure 2. The majority of RCTs exhibited low RoB in the randomization process (*n* = 68.8%) and in dealing with missing outcome data (*n* = 87.5%). Owing to insufficient reporting of specific randomization methods, 30% of the RCTs were appraised as having some concerns in the randomization process. Some studies exhibited a high RoB owing to missing outcome data, loss of follow-up, and unreported specific reasons. Detailed results of each study are demonstrated in Appendix B.

### 3.4 NMA

#### 3.4.1 PSQI

##### 3.4.1.1 PSQI within 4 weeks

**Figure 3:**
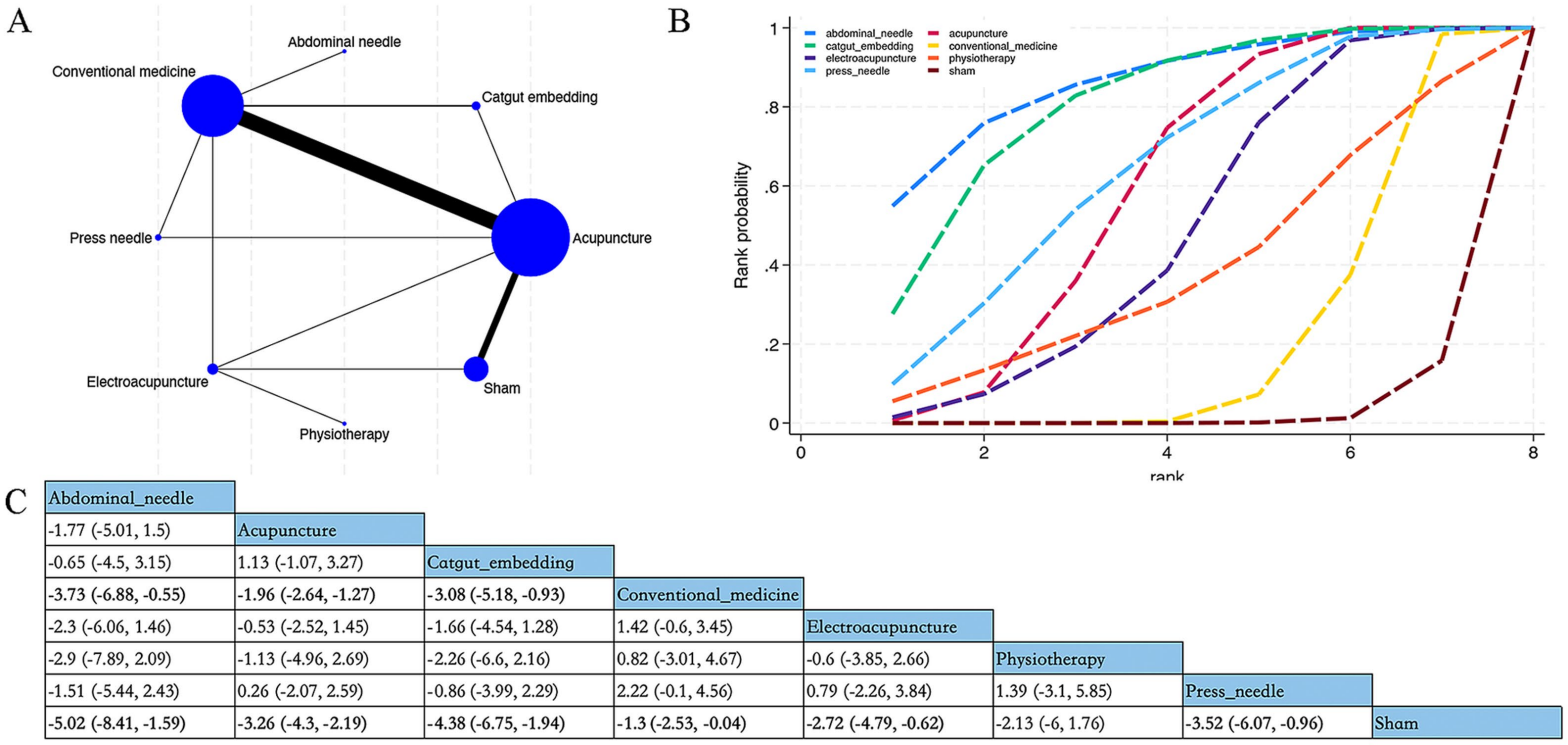
Network plot, NMA results, and SUCRA plot. **(A)** Network plot for PSQI within 4 weeks; **(B)** relative effects of varied interventions on PSQI within 4 weeks; **(C)** SUCRA plot for PSQI within 4 weeks. Estimates are depicted as MD with 95% CrI (in brackets). Treatment comparisons are displayed horizontally (left to right). Supplementary effect estimates appear at column-row intersections. Significant results are exhibited in bold.

Overall, 38 RCTs estimated the effects of 5 varied acupuncture therapies on the short-term effects of the PSQI (treatment duration: 4 weeks) (Figure 3A). The dominant findings from the NMA are demonstrated in Figure 3B. Relative to conventional medication, abdominal acupuncture (MD −3.73; 95% CrI [−6.88, −0.55]), acupuncture (MD −1.96; 95% CrI [−2.64, −1.27]), and catgut embedding (MD −3.08; 95% CrI [−5.18, −0.93]) considerably diminished PSQI scores of patients within 4 weeks. Based on SUCRA, abdominal acupuncture may demonstrate potential advantages in reducing PSQI scores over the 4-week period (SUCRA = 86%) (Figure 3C) (Appendix C).

##### 3.4.1.2 Long-term effects on PSQI

**Figure 4:**
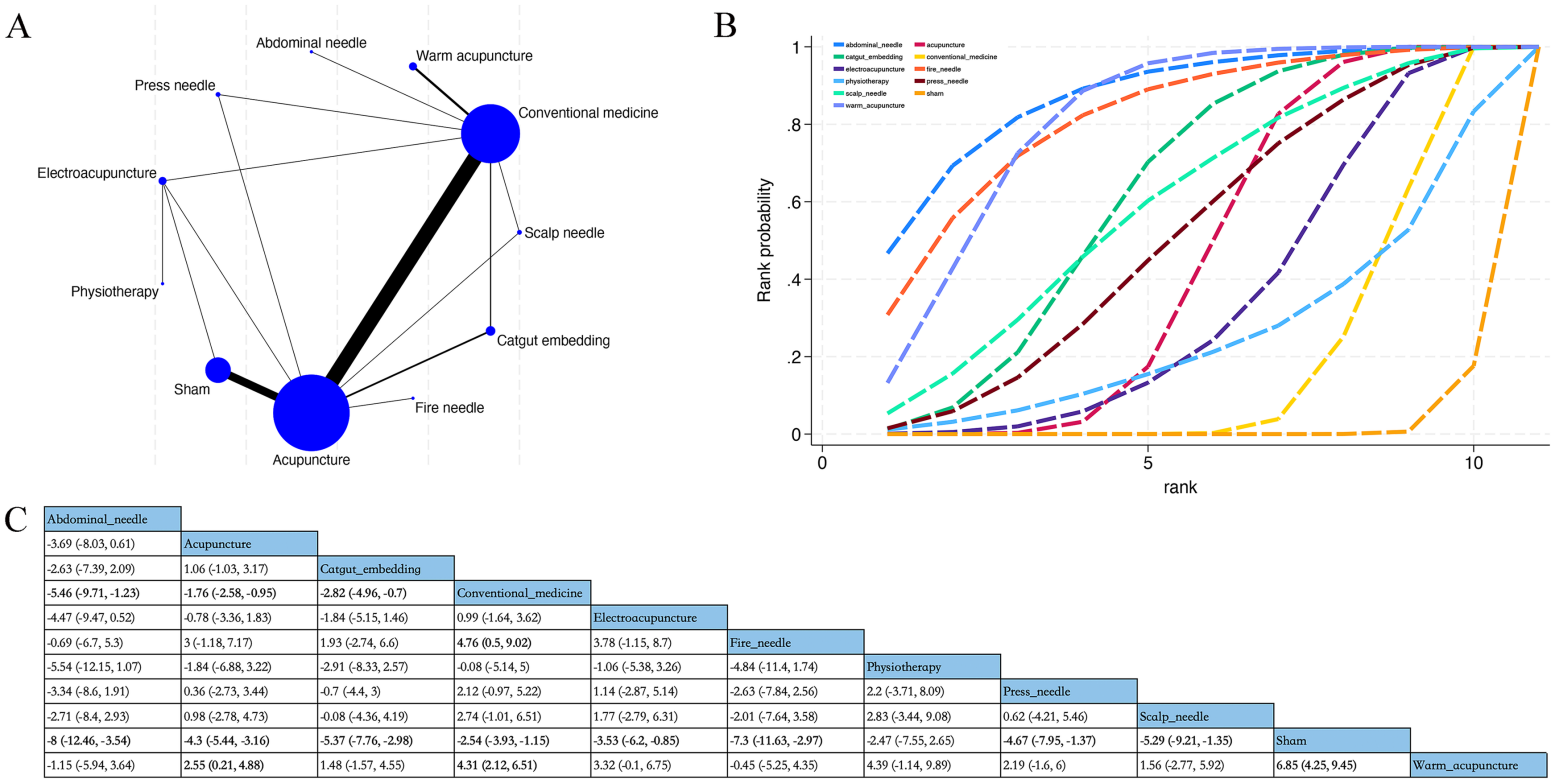
Network plot, NMA results, and SUCRA plot. **(A)** Network plot for PSQI maximum time (score); **(B)** relative effects of varied interventions on PSQI maximum time; **(C)** SUCRA plot for PSQI maximum time. Estimates are depicted as MD with 95% CrI (in brackets). Treatment comparisons are displayed horizontally (left to right). Supplementary effect estimates appear at column-row intersections. Significant results are exhibited in bold.

Overall, 55 RCTs estimated the effects of 8 varied acupuncture therapies on the long-term effects of PSQI (treatment duration ≥4 weeks) (Figure 4A). The dominant findings from the NMA are exhibited in Figure 4B. Relative to conventional medication, abdominal acupuncture (MD −5.46; 95% CrI [−9.71, −1.23]), acupuncture (MD −1.76; 95% CrI [−2.58, −0.95]), catgut embedding (MD −2.82; 95% CrI [−4.96, −0.7]), fire needle (MD- 4.76; 95% CrI [−9.02, −0.5]), and warm acupuncture (MD −4.31; 95% CrI [−6.51, −2.12]) considerably diminished long-term PSQI scores of patients. Relative to acupuncture, warm acupuncture (MD −2.55; 95% CrI [−4.88, −0.21]) considerably diminished long-term PSQI scores of patients (Figure 4B). Based on SUCRA, abdominal acupuncture may demonstrate potential advantages in reducing long-term PSQI scores (SUCRA = 87%; Figure 4C; Appendix C).

#### 3.4.2 Anxiety scores

**Figure 5:**
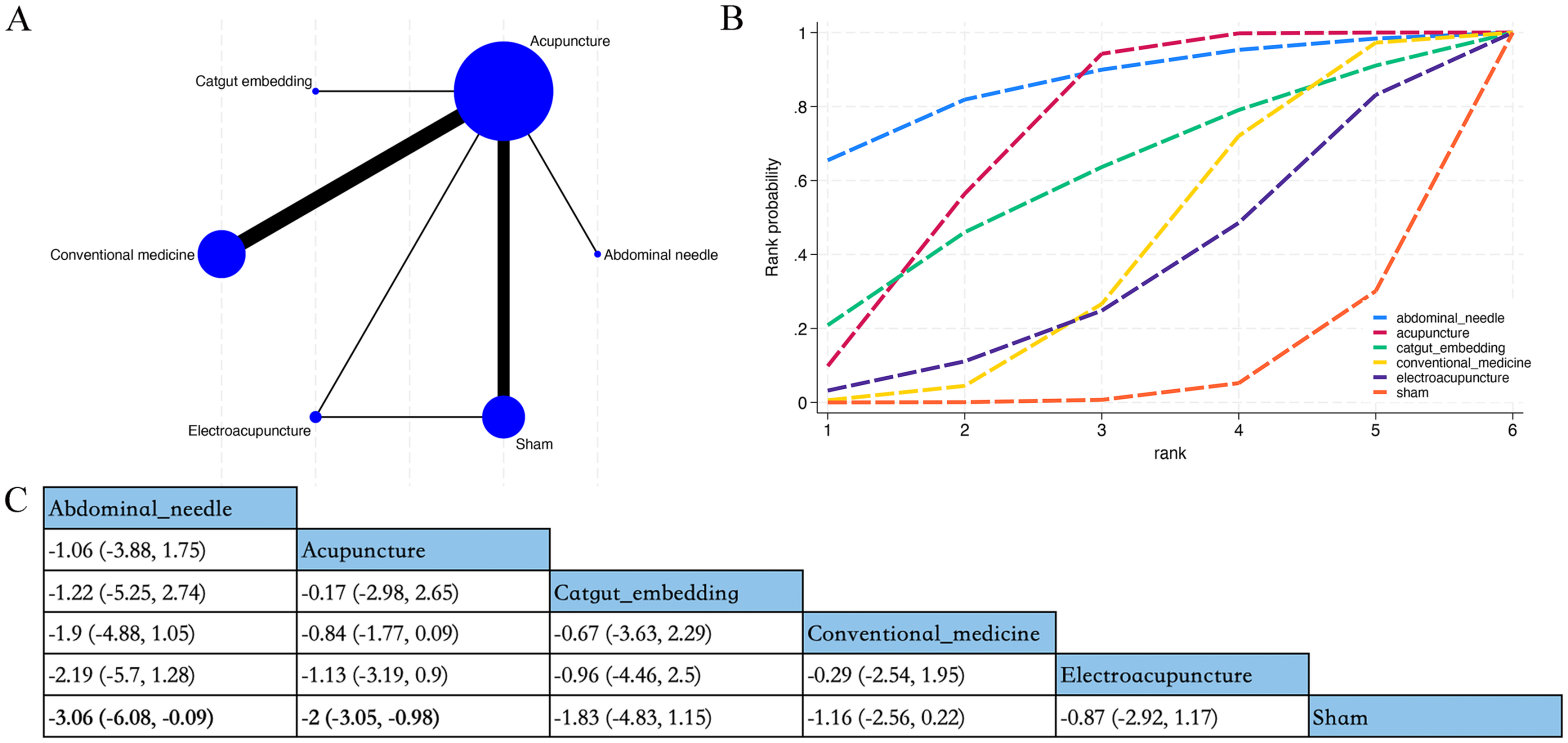
Network plot, NMA results, and SUCRA plot. **(A)** Network plot for anxiety scores; **(B)** relative effects of varied interventions on anxiety scores; **(C)** SUCRA plot for anxiety scores. Estimates are depicted as MD with 95% CrI (in brackets). Treatment comparisons are displayed horizontally (left to right). Supplementary effect estimates appear at column-row intersections. Significant results are exhibited in bold.

Overall, 20 RCTs estimated the effects of 4 varied acupuncture therapies on anxiety scores (Figure 5A). The dominant findings from the NMA are demonstrated in Figure 5B. Relative to sham acupuncture, abdominal acupuncture (SMD −3.06; 95% CrI [−6.08, −0.09]) and acupuncture (SMD −2.00; 95% CrI [−3.05, −0.98]) considerably diminished anxiety scores of patients (Figure 5B). Based on SUCRA, abdominal acupuncture may demonstrate potential advantages in reducing anxiety scores (SUCRA = 86%) (Figure 5C; Appendix C).

#### 3.4.3 Depression scores

**Figure 6:**
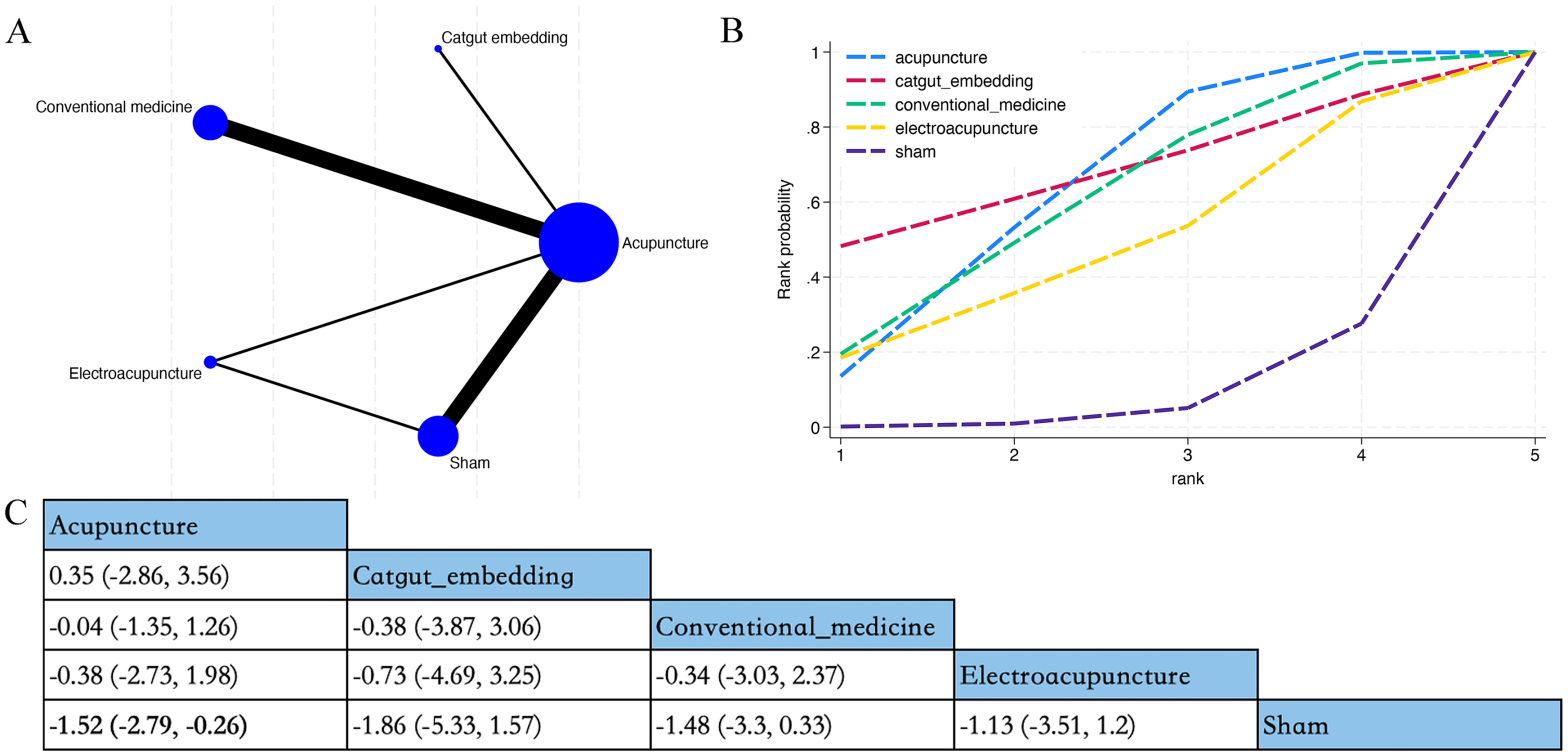
Network plot, NMA results, and SUCRA plot. **(A)** Network plot for depression scores (points); **(B)** relative effects of varied interventions on depression scores; **(C)** SUCRA plot for depression scores. Estimates are depicted as MD with 95% CrI (in brackets). Treatment comparisons are displayed horizontally (left to right). Supplementary effect estimates appear at column-row intersections. Significant results are exhibited in bold.

Overall, 15 RCTs estimated the effects of 3 varied acupuncture therapies on depression scores (Figure 6A). The dominant findings from the NMA are demonstrated in Figure 6B. Relative to sham acupuncture, acupuncture (SMD −1.52; 95% CrI [−2.79, −0.26]) considerably diminished depression scores of patients (Figure 6B). Based on SUCRA, catgut embedding may exhibit potential superiority in reducing depression scores (SUCRA = 68%) (Figure 6C; Appendix C).

#### 3.4.4 TCM syndrome scores

**Figure 7:**
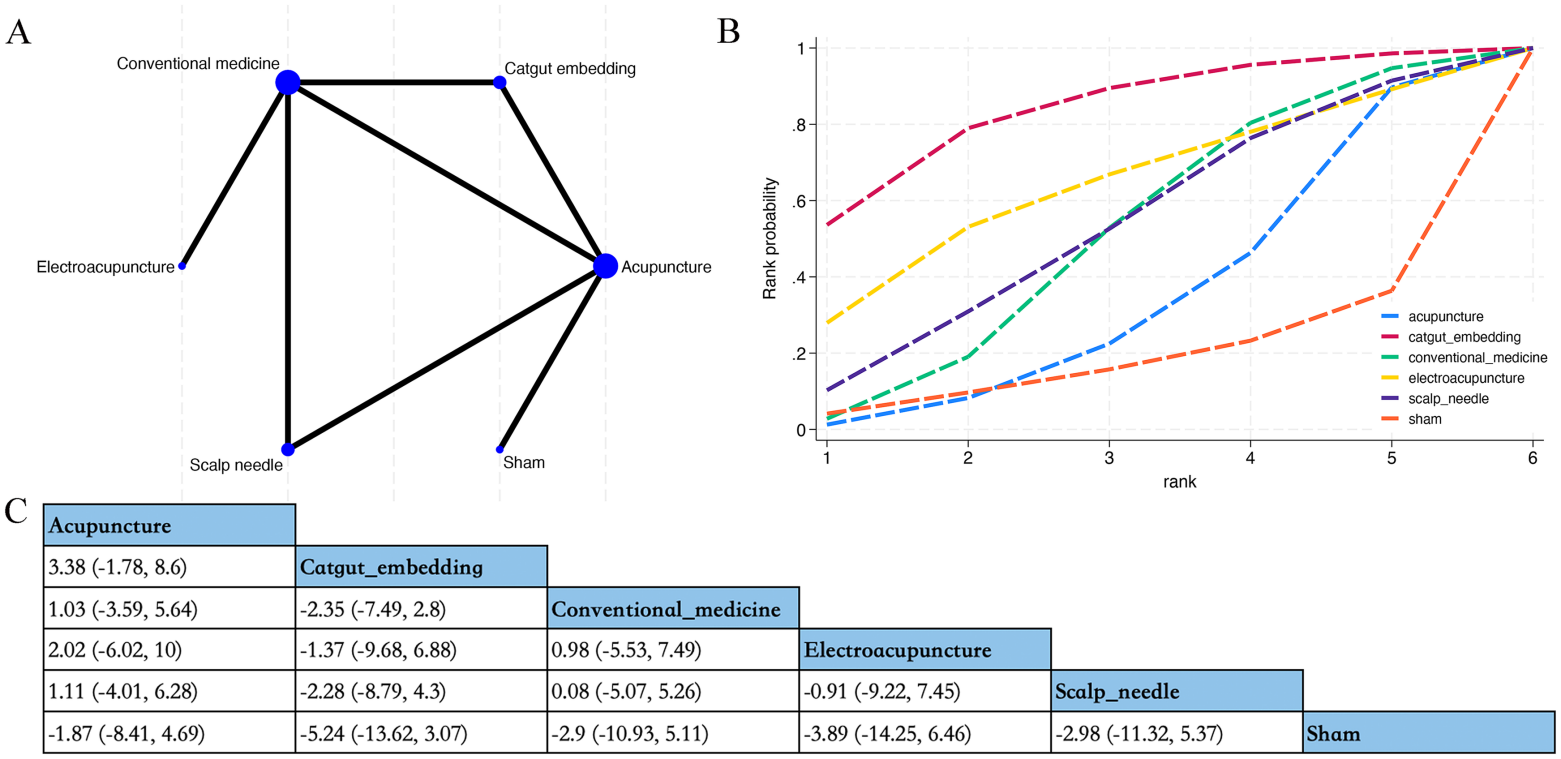
Network plot, NMA results, and SUCRA plot. **(A)** Network plot for TCM syndrome scores (points); **(B)** relative effects of varied interventions on TCM syndrome scores; **(C)** SUCRA plot for TCM syndrome scores. Estimates are depicted as MD with 95% CrI (in brackets). Treatment comparisons are displayed horizontally (left to right). Supplementary effect estimates appear at column-row intersections. Significant results are exhibited in bold.

Overall, five RCTs estimated the effects of five varied acupuncture therapies on TCM syndrome scores (Figure 7A). The dominant findings from the NMA are demonstrated in Figure 7B. No marked distinctions in the comparative effects among these interventions were detected (Figure 7B). Based on SUCRA, catgut embedding may demonstrate potential superiority in reducing TCM syndrome scores (SUCRA = 83%) (Figure 7C; Appendix C).

#### 3.4.5 Clinical efficacy rates

**Figure 8:**
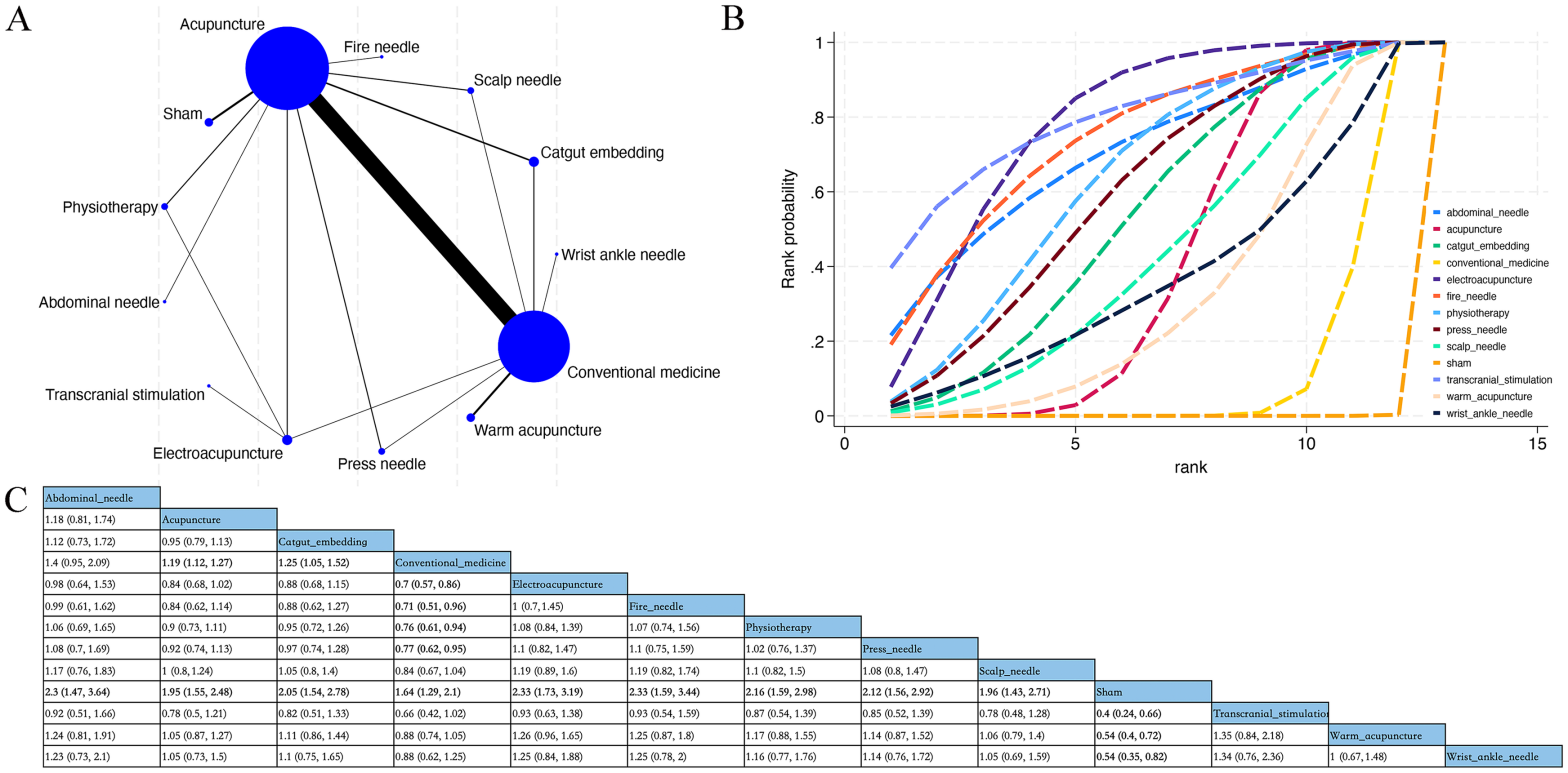
Network plot, NMA results, and SUCRA plot. **(A)** Network plot for clinical efficacy rates; **(B)** relative effects of varied interventions on clinical efficacy rates; **(C)** SUCRA plot for clinical efficacy rates. Estimates are depicted as MD with 95% CrI (in brackets). Treatment comparisons are displayed horizontally (left to right). Supplementary effect estimates appear at column-row intersections. Significant results are exhibited in bold.

Overall, 57 RCTs estimated the effects of 10 varied acupuncture therapies on clinical efficacy rates (Figure 8A). The dominant findings from the NMA are demonstrated in Figure 8B. Relative to conventional Western medicine, acupuncture (RR 1.19; 95% CrI [1.12, 1.27]), catgut embedding (RR 1.25; 95% CrI [1.05, 1.52]), electroacupuncture (RR 0.70; 95% CrI [0.57, 0.86]), fire needle (RR 0.71; 95% CrI [0.51, 0.96]), physiotherapy (RR 0.76; 95% CrI [0.61, 0.94]), and press needle (RR 0.77; 95% CrI [0.62, 0.95]) considerably improved clinical efficacy rates in patients (Figure 8B). No marked distinctions were detected in the relative effects of varied acupuncture therapies. Based on SUCRA, electroacupuncture may demonstrate potential advantages in enhancing clinical efficacy (SUCRA = 78%) (Figure 8C; Appendix C).

#### 3.4.6 Adverse events

**Figure 9:**
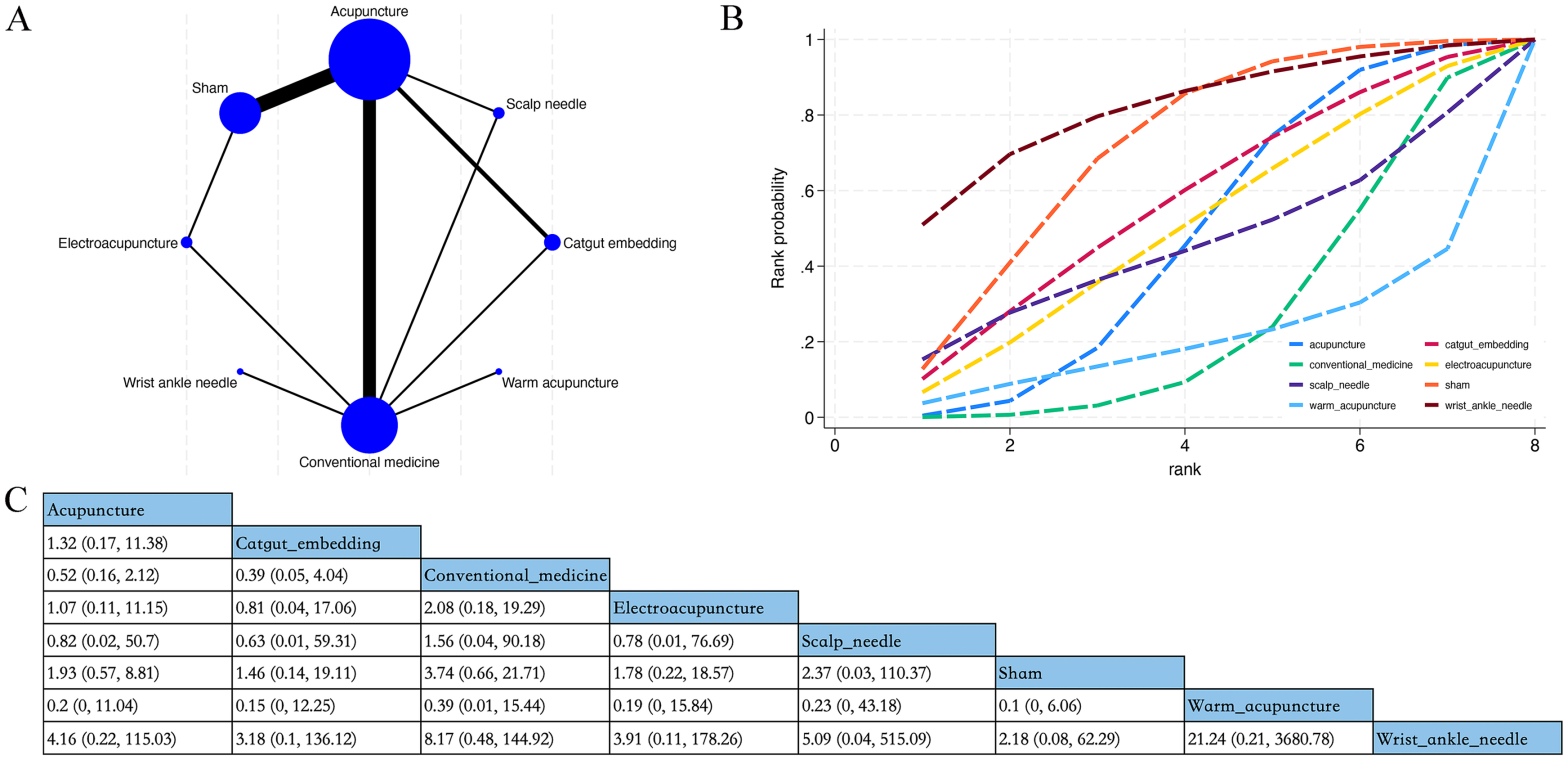
Network plot, NMA results, and SUCRA plot. **(A)** Network plot for the adverse event occurrence; **(B)** relative effects of varied interventions on the adverse event occurrence; **(C)** SUCRA plot for the occurrence of adverse events. Estimates are depicted as MD with 95% CrI (in brackets). Treatment comparisons are displayed horizontally (left to right). Supplementary effect estimates appear at column-row intersections. Significant results are exhibited in bold.

Overall, 18 RCTs estimated the effects of 6 varied acupuncture therapies on adverse events (Figure 9A). The dominant findings from the NMA are demonstrated in Figure 9B. Based upon the NMA results, no acupuncture method considerably diminished the adverse event incidence, and no marked distinctions were detected in the relative effects (Figure 9B). The cumulative sample size for acupuncture therapies included in the safety analysis was 1,772. In total, 99 adverse events were reported (5.59%). The most common adverse events associated with acupuncture interventions included pain, hematoma, and dizziness, primarily involving transient and localized reactions during needle insertion (Appendix D). Based on SUCRA, wrist ankle needle may demonstrate a relatively favorable safety profile (SUCRA = 82%) (Figure 9C; Appendix C).

### 3.5 Assessment of heterogeneity, consistency, and publication bias

Heterogeneity was assessed using the *I*^2^ statistic. For all outcome measures (PSQI, anxiety scores, depression scores, TCM syndrome scores, clinical efficacy rate, and the incidence of adverse events), the *I*^2^ values exceeded 50%. This indicated significant heterogeneity (Appendix E). The DIC was leveraged to contrast the consistency and inconsistency model. The variations in every closed-loop model were <5, indicating good consistency with DIC. Local inconsistency was estimated for PSQI, anxiety scores, depression scores, TCM syndrome scores, clinical efficacy rates, and adverse event incidence. No marked local inconsistency was detected (Table 3). Regarding assessment of publication bias, potential publication bias was observed for PSQI, anxiety scores, depression scores, clinical efficacy rates, and the incidence of adverse events in the comparison-adjusted funnel plot (Appendix F).

**Table 3:** Local inconsistency.

Outcomes	Comparison	Direct	Indirect	Network	*p*-value CrI
PSQI 4	Catgut embedding vs. acupuncture	−1.83 (−4.76,1.10)	1.56 (−1.61,4.75)	−1.13 (−3.29,1.06)	0.12195
	Conventional medicine vs. acupuncture	1.94 (1.28,2.61)	3.24 (0.368,6.05)	1.95 (1.27,2.65)	0.3744
	Electroacupuncture vs. acupuncture	−0.0153 (−3.61,3.59)	0.746 (−1.62,3.17)	0.545 (−1.46,2.51)	0.726775
	Press needle vs. acupuncture	−0.202 (−3.46,3.05)	−0.341 (−3.75,3.09)	−0.266 (−2.62,2.08)	0.955875
	Sham vs. acupuncture	3.46 (2.39,4.51)	0.351 (−3.65,4.37)	3.26 (2.18,4.29)	0.13885
	Electroacupuncture vs. conventional medicine	−2.73 (−5.91,0.441)	−0.535 (−3.15,2.02)	−1.41 (−3.44,0.568)	0.284025
	Press needle vs. conventional medicine	−2.29 (−5.65,1.09)	−2.15 (−5.50,1.18)	−2.22 (−4.56,0.122)	0.95365
	Sham vs. electroacupuncture	0.835 (−2.41,4.04)	3.94 (1.33,6.49)	2.72 (0.653,4.80)	0.138125
PSQI maximum time	Catgut embedding vs. acupuncture	−1.60 (−4.10,0.903)	1.48 (−3.05,6.00)	−1.07 (−3.16,1.04)	0.235575
	Conventional medicine vs. acupuncture	1.75 (0.896,2.63)	1.76 (−1.49,5.04)	1.77 (0.948,2.59)	0.9969
	Electroacupuncture vs. acupuncture	−0.0146 (−4.68,4.60)	1.18 (−2.01,4.37)	0.787 (−1.81,3.40)	0.672725
	Press needle vs. acupuncture	−0.192 (−4.51,4.15)	−0.533 (−5.03,3.96)	−0.356 (−3.46,2.74)	0.9113
	Sham vs. acupuncture	4.49 (3.33, 5.62)	0.354 (−4.99,5.66)	4.30 (3.16,5.44)	0.133575
	Conventional medicine vs. catgut embedding	2.35 (−0.786, 5.45)	3.35 (0.123, 6.54)	2.83 (0.685,4.97)	0.652425
	Electroacupuncture vs. conventional medicine	−2.76 (−7.04,1.54)	0.0920 (−3.26,3.42)	−0.980 (−3.62,1.66)	0.297975
	Press needle vs. conventional medicine	−2.28 (−6.70,2.10)	−1.98 (−6.38,2.46)	−2.12 (−5.22,0.978)	0.923075
	Sham vs. electroacupuncture	0.923 (−3.42,5.26)	5.04 (1.70,8.35)	3.52 (0.818,6.19)	0.138375
Clinical effective rate	Catgut embedding vs. acupuncture	1.67 (0.637,4.37)	0.330 (0.0364,2.94)	1.29 (0.550,3.03)	0.175675
	Conventional medicine vs. acupuncture	0.300 (0.219,0.404)	0.825 (0.199,3.22)	0.312 (0.230,0.418)	0.16475
	Electroacupuncture vs. acupuncture	3.29 (1.03,11.3)	4.39 (0.909,24.6)	3.63 (1.41,9.67)	0.7733
	Physiotherapy vs. acupuncture	3.21 (0.856,12.8)	2.25 (0.313,16.2)	2.84 (0.971,8.48)	0.7641
	Press needle vs. acupuncture	3.49 (0.921,14.6)	0.727 (0.107,5.59)	2.11 (0.699,6.63)	0.20005
	Conventional medicine vs. catgut embedding	0.286 (0.0806,1.02)	0.240 (0.0641,0.890)	0.241 (0.101,0.575)	0.84785
	Electroacupuncture vs. conventional medicine	14.4 (1.43,469)	11.6 (4.06,34.8)	11.6 (4.41,32.2)	0.877775
	Press needle vs. conventional medicine	2.37 (0.363,18.0)	11.4 (2.99,48.7)	6.77 (2.22,21.8)	0.193125
	Scalp needle vs. conventional medicine	4.02 (0.541,42.9)	3.20 (0.0655,144)	4.07 (0.821,25.7)	0.897325
	Physiotherapy vs. electroacupuncture	0.652 (0.121,3.32)	0.958 (0.171,5.33)	0.778 (0.240,2.51)	0.75115
Anxiety score	Electroacupuncture vs. acupuncture	0.361 (−2.50,3.23)	2.00 (−1.04,5.07)	1.14 (−0.910,3.20)	0.41112
	Sham vs. acupuncture	2.12 (1.04,3.23)	0.471 (−3.56,4.52)	2.00 (0.975,3.05)	0.4104
	Sham vs. electroacupuncture	0.107 (−2.73,2.96)	1.74 (−1.31,4.80)	0.866 (−1.18,2.93)	0.41163
Depression score	Electroacupuncture vs. acupuncture	−0.468 (−3.78,2.85)	1.37 (−2.20,4.95)	0.385 (−1.94,2.74)	0.41905
	Sham vs. acupuncture	1.66 (0.317,3.03)	−0.170 (−4.83,4.52)	1.52 (0.269, 2.81)	0.41246
	Sham vs. electroacupuncture	0.290 (−3.02,3.57)	2.13 (−1.46, 5.76)	1.14 (−1.21,3.49)	0.41685
TCM syndrome score	Catgut embedding vs. acupuncture	−5.31 (−9.76,-0.845)	−0.347 (−6.07,5.36)	−3.39 (−8.64,1.72)	0.10125
	Conventional medicine vs. acupuncture	0.181 (−6.81,7.12)	−2.26 (−9.24,4.74)	−1.04 (−5.70,3.52)	0.53476
	Scalp needle vs. acupuncture	−0.495 (−7.67,6.64)	−2.13 (−11.4,7.15)	−1.12 (−6.30,4.01)	0.72979
	Conventional medicine vs. catgut embedding	0.531 (−3.86,4.93)	5.48 (−0.289,11.2)	2.34 (−2.81,7.54)	0.10059
	Scalp needle vs. conventional medicine	−0.698 (−7.87,6.50)	0.931 (−8.35,10.2)	−0.0788 (−5.22,5.08)	0.72865
Adverse event rate	Conventional medicine vs. acupuncture	2.72 (0.224,23.0)	0.137 (0.0000359,261)	2.36 (0.267,14.5)	0.411375
	Sham vs. acupuncture	0.284 (0.0210,2.00)	6.23 (0.00319,8,350)	0.376 (0.0357,2.29)	0.370525
	Conventional medicine vs. catgut embedding	8.03 (0.0300,2,260)	1.10 (0.00190,421)	2.93 (0.0800,88.4)	0.5844
	Electroacupuncture vs. conventional medicine	1.73 (0.0104,307)	0.0847 (0.000239,21.3)	0.415 (0.0108,16.9)	0.376425
	Sham vs. electroacupuncture	1.14 (0.00955,133)	0.0552 (0.000114,18.6)	0.382 (0.00915,12.5)	0.376

## 4 Discussion

Utilizing NMA, this research included 80 studies to estimate the potency and safety of varied acupuncture therapies among individuals with PI, including short-term (4-week) and long-term effects on PSQI scores, anxiety, depression, TCM syndrome scores, and clinical efficacy rates. The results indicated that abdominal acupuncture is superior for improving PSQI and anxiety scores (both short- and long-term). Catgut embedding exhibits greater efficacy in reducing depression and TCM syndrome scores. Electroacupuncture is more effective in improving clinical efficacy rates. Wrist ankle needle demonstrates a potential advantage in lower incidence of adverse events, though further validation is required.

Based on NMA findings, abdominal acupuncture may demonstrate potential advantages in improving both short-term and long-term PSQI scores. Research by Kim et al. (98) exhibited that, relative to conventional medications, most included studies detected significant effects of acupuncture in reducing PSQI within 4 weeks. Zhang et al. (99) detected that the optimal duration for acupuncture was 3–4 weeks. Thus, we implemented Bayesian NMA to compare PSQI within 4 weeks and the longest duration of PSQI (≥4 weeks). The results differ from previous findings (100). Fang et al. (100) reported seven studies on PSQI outcomes, which exhibited significant heterogeneity. Their results indicated no statistical distinctions between acupuncture and conventional medications in diminishing PSQI scores. However, our analysis detected that abdominal acupuncture, acupuncture, and catgut embedding considerably diminished PSQI scores relative to conventional medications. This might be attributed to the updated study design, larger sample size, and more diverse acupuncture interventions included in this research. Regarding PSQI within 4 weeks, this research detected marked efficacy with abdominal acupuncture, acupuncture, and catgut embedding relative to conventional medications. Nevertheless, no marked distinctions were detected among varied acupuncture methods. Warm acupuncture exhibits better long-term efficacy than acupuncture, while the efficacy of fire needle and warm acupuncture for PSQI within 4 weeks remains underexplored. Based on SUCRA, abdominal acupuncture may demonstrate promising advantages in reducing PSQI scores. This differs from the findings of Lu et al. (11), whose study detected that the best results were achieved with catgut embedding when abdominal acupuncture was not included. Therefore, future research should include more evidence to verify this discrepancy, which might be owing to distinctions in the acupuncture interventions included. The advantages of abdominal acupuncture might be attributed to the rich autonomic nerve plexus in the abdomen. The short-term effects of abdominal acupuncture are likely more dependent on the rapid regulation of neural reflexes, directly and quickly adjusting imbalanced organ functions by stimulating abdominal meridian points, with a short path and quick effect. Its long-term effects involve deeper physiological changes that require time to accumulate. The long-term, regular stimulation of abdominal acupuncture can optimize intestinal function and indirectly promote the stable secretion of endogenous melatonin, thus stabilizing the sleep–wake cycle over time. It can also inhibit the overactive hypothalamic–pituitary–adrenal axis, thereby restoring normal hormonal circadian rhythms.

There were no marked distinctions among the varied acupuncture methods in reducing anxiety and depression scores. This might be attributed to inconsistent rating scales and insufficient inclusion of relevant studies. Based upon SUCRA, we detected that abdominal acupuncture exhibited an advantage in diminishing anxiety scores. This might be attributed to the fact that abdominal acupuncture typically uses fine needles with shallow insertion and gentle techniques, resulting in a treatment process that is either painless or causes minimal pain. This characteristic in itself helps alleviate anxiety and promote parasympathetic nervous system excitation. Catgut embedding exhibited a greater advantage in diminishing depression scores. Catgut embedding may reinforce the effectiveness of acupuncture, providing stronger and more durable effects. Following the catgut embedding procedure, sterile inflammation is induced. This stimulates tissue repair and adjustment mechanisms, triggering a self-healing effect. Nevertheless, the quantity of studies on these outcome measures is limited, and further validation is required in future research. Regarding TCM syndrome scores, there were no marked distinctions in the relative effects between the five acupuncture methods and three non-acupuncture interventions. However, based on SUCRA, catgut embedding may hold potential advantages. This might be because catgut embedding provides mild and sustained physiological stimulation to the acupoints, leading to a long-lasting therapeutic effect, which aligns with prior evidence (11).

Electroacupuncture may demonstrate potential advantages in improving clinical efficacy rates, which is inconsistent with prior evidence (11, 12). This might be owing to distinctions in clinical practice and trial design. Traditional acupuncture therapies heavily rely on the operator’s technique (frequency, amplitude, and intensity of the needling) and the patient’s sensation of ‘deqi’. Furthermore, given the differences in the criteria for determining clinical efficacy rates among the original studies, the findings should be interpreted with caution. These factors are highly variable and difficult to standardize. Electroacupuncture uses equipment to deliver pulsed currents with standardized, quantifiable, and rhythmic stimulation, allowing for a more direct and effective intervention on the neurophysiological basis of insomnia. This unique characteristic might be the key to its elevated efficacy rates. Numerous acupuncture therapies are superior to sham acupuncture, but there were no marked distinctions in relative effectiveness among varied acupuncture methods. This finding aligns with prior evidence (11, 100), which might be owing to variations in treatment duration, acupoint selection, or the skill levels of acupuncturists. Numerous acupuncture therapies exhibited marked distinctions relative to conventional medications, which are inconsistent with prior evidence (100). We speculate that this discrepancy may be due to the continuous evolution of research methodology in the field of acupuncture, as recent RCTs have become more standardized regarding trial design and operator training.

Eighteen studies reported adverse events. No severe adverse events linked to acupuncture were observed across all studies, which aligns with prior evidence (11, 101). Based upon the NMA results, no acupuncture method considerably diminished the incidence of adverse events. Also, no marked distinctions in relative effects were detected. According to SUCRA rankings, wrist-ankle needle may show higher potential safety. However, due to inconsistent monitoring and reporting standards for adverse events across included studies, this analysis solely extracted and pooled explicitly reported adverse events. Consequently, comparative results of adverse events should be interpreted with caution.

This research has numerous strengths, primarily stemming from the inclusion of the most recent and comprehensive evidence. Regarding primary outcomes, we estimated the short-term effects of the PSQI (4 weeks), providing detailed treatment duration-related improvement data. Also, we estimated the long-term effects of the PSQI to observe overall therapeutic changes. This research has several limitations. First, despite including diverse acupuncture modalities, some techniques were underrepresented in the literature, warranting cautious interpretation of related findings. Future studies should validate these results. What’s more, methodological heterogeneity (e.g., varying acupoints, needle retention times, treatment frequencies) even within the same acupuncture technique necessitates cautious interpretation. Moreover, complete blinding in acupuncture procedures remains unattainable. Future research requires stricter designs and assessment criteria. Finally, the restriction to English and Chinese publications might introduce language bias. Future research ought to include more studies in varied languages for validation. Based upon NMA results, future research should prioritize abdominal acupuncture, catgut embedding, electroacupuncture, and wrist ankle needle for systematic investigation across diverse populations and regions.

## 5 Conclusion

Abdominal acupuncture may exhibit relative advantages in improving both short-term and long-term PSQI scores and alleviating anxiety symptoms, whereas catgut embedding may demonstrate greater benefits in reducing depression and TCM syndrome scores. Meanwhile, electroacupuncture may show certain superiority in enhancing overall clinical efficacy rates. Future research should focus on single-modality interventions, adopt more rigorous experimental designs and evaluation criteria, and analyze differences in the efficacy of different acupoint prescriptions for primary insomnia, thereby exploring optimal protocols to further validate the findings of this study.

## Data Availability

The original contributions presented in the study are included in the article/Supplementary material, further inquiries can be directed to the corresponding authors.
