# Supplementary material for: Comparative effectiveness and safety of acupuncture treatments for primary insomnia: a systematic review and network meta-analysis of randomized trial
Source: Front Neurol. 2026 Mar 3;17:1750474. doi: 10.3389/fneur.2026.1750474 (PMC12992266; doi:10.3389/fneur.2026.1750474)
Supplement: Supplementary file 1 [file Table_1.DOCX]

**Appendix A:** Search strategies

**Pubmed**

**#1** "Acupuncture"[Mesh]

**#2** "Acupuncture therapy"[Mesh]

**#3** "Acupuncture, Ear"[Mesh]

**#4** "Acupuncture Points"[Mesh]

**#5** "Acupuncture Analgesia"[Mesh]

**#6** "Dry Needling"[Mesh]

**#7** "Electroacupuncture"[Mesh]

**#8** Acupuncture* OR Pharmacopuncture OR Pharmacoacupuncture Treatment OR Pharmacoacupuncture Therapy OR Acupotom* OR Acupoint* OR dry needl* OR catgut implantation OR burnt needle therapy OR fire needl* OR thermoacupuncture OR scalp needle OR auriculoacupuncture OR auriculotherapy OR warm needling OR head penetration needling OR abdominal needle OR wrist ankle needle OR triple puncture OR manual-acupuncture OR the therapy of eye-acupuncture OR oculo-acupuncture therapy OR auriculo-acupuncture OR thermo-acupuncture OR electro-acupuncture OR intramuscular stimulation OR Electroacupuncture [Title/Abstract]

**#9** #1 OR #2 OR #3 OR #4 OR #5 OR #6 OR #7 OR #8

**#10** "Sleep Initiation and Maintenance Disorders"[Mesh]

**#11** Insomnia* OR Disorders of Initiating AND Maintaining Sleep OR Sleeplessness OR Early Awakening OR Primary Insomnia OR Sleep Initiation Dysfunction* OR Wakeful* [Title/Abstract]

**#12** #10 OR #11

**#13** randomized controlled trial OR controlled clinical trial OR randomized OR placebo OR randomly OR trial OR random* OR placebo* OR group* OR control* [Title/Abstract]

**#14** #9 AND #12 AND #13

**Cochrane Library**

**#1** MeSH descriptor: [Sleep Initiation and Maintenance Disorders] explode all trees

**#2** Insomnia* OR Disorders of Initiating AND Maintaining Sleep OR Sleeplessness OR Early Awakening OR Primary Insomnia OR Sleep Initiation Dysfunction* OR Wakeful* :ti,ab,kw

**#3** #1 OR #2

**#4** MeSH descriptor: [Acupuncture] explode all trees

**#5** MeSH descriptor: [Acupuncture Therapy] explode all trees

**#6** MeSH descriptor: [Acupuncture, Ear] explode all trees

**#7** MeSH descriptor: [Acupuncture Points] explode all trees

**#8** MeSH descriptor: [Acupuncture Analgesia] explode all trees

**#9** MeSH descriptor: [Dry Needling] explode all trees

**#10** MeSH descriptor: [Electroacupuncture] explode all trees

**#11** Acupuncture* OR Pharmacopuncture OR Pharmacoacupuncture Treatment OR Pharmacoacupuncture Therapy OR Acupotom* OR Acupoint* OR dry needl* OR catgut implantation OR burnt needle therapy OR fire needl* OR thermoacupuncture OR scalp needle OR auriculoacupuncture OR auriculotherapy OR warm needling OR head penetration needling OR abdominal needle OR wrist ankle needle OR triple puncture OR manual-acupuncture OR the therapy of eye-acupuncture OR oculo-acupuncture therapy OR auriculo-acupuncture OR thermo-acupuncture OR electro-acupuncture OR intramuscular stimulation OR Electroacupuncture :ti,ab,kw

**#12** #4 OR #5 OR #6 OR #7 OR #8 OR #9 OR #10 OR #11

**#13** randomized controlled trial OR controlled clinical trial OR randomized OR placebo OR randomly OR trial OR random* OR placebo* OR group* OR control* :ti,ab,kw

**#14** #3 AND #12 AND #13

**Web of Science**

TS=(Insomnia* OR Disorders of Initiating and Maintaining Sleep OR Sleeplessness OR Early Awakening OR Primary Insomnia OR Sleep Initiation Dysfunction* OR Wakeful*) AND TS=(Acupuncture* OR Pharmacopuncture OR Pharmacoacupuncture Treatment OR Pharmacoacupuncture Therapy OR Acupotom* OR Acupoint* OR dry needl* OR catgut implantation OR burnt needle therapy OR fire needl* OR thermoacupuncture OR scalp needle OR auriculoacupuncture OR auriculotherapy OR warm needling OR head penetration needling OR abdominal needle OR wrist ankle needle OR triple puncture OR manual-acupuncture OR the therapy of eye-acupuncture OR oculo-acupuncture therapy OR auriculo-acupuncture OR thermo-acupuncture OR electro-acupuncture OR intramuscular stimulation OR Electroacupuncture) AND TS=(randomized controlled trial OR controlled clinical trial OR randomized OR placebo OR randomly OR trial OR random* OR placebo* OR group* OR control*)

**Embase**

**#1** 'primary insomnia'/exp

**#2 insomnia* OR ('disorders of initiating':ab,ti AND 'maintaining sleep':ab,ti) OR sleeplessness OR 'early awakening' OR 'primary insomnia' OR 'sleep initiation dysfunction*' OR wakeful* :ab,ti**

**#3 #1 OR #2**

**#4 'acupuncture'/exp OR 'electroacupuncture'/exp OR 'dry needling'/exp OR 'catgut embedding'/exp OR 'body acupuncture'/exp OR 'warm acupuncture'/exp OR 'filiform needle'/exp OR 'manual acupuncture'/exp OR 'auricular acupuncture'/exp OR 'scalp vein needle'/exp OR 'plum blossom needle'/exp**

**#5 acupuncture* OR pharmacopuncture OR 'pharmacoacupuncture treatment' OR 'pharmacoacupuncture therapy' OR acupotom* OR acupoint* OR 'dry needl*' OR 'catgut implantation' OR 'burnt needle therapy' OR 'fire needl*' OR thermoacupuncture OR 'scalp needle' OR auriculoacupuncture OR auriculotherapy OR 'warm needling' OR 'head penetration needling' OR 'abdominal needle'i OR 'wrist ankle needle' OR 'triple puncture' OR 'manual acupuncture' OR 'the therapy of eye-acupuncture' OR 'oculo-acupuncture therapy' OR 'auriculo acupuncture' OR 'thermo acupuncture' OR 'electro acupuncture' OR 'intramuscular stimulation' OR electroacupuncture OR 'catgut embedding' OR 'scalp vein needle' OR 'plum blossom needle' :ab,ti**

**#6 #4 OR #5**

**#7 'randomized controlled trial' OR 'controlled clinical trial' OR randomized OR placebo OR randomly OR trial OR random* OR placebo* OR group* OR control* :ab,ti**

**#8 #3 AND #6 AND #7**

**VIP**

(原发性失眠+失眠+不寐+睡眠障碍+睡眠起始功能障碍+早醒+入睡和睡眠失调) AND (针灸+针刺+体针+电针+温针+耳埋+耳针+耳廓针刺术+头皮针+头针+火针+燔针+腹针+眼针+腕踝针+埋线+皮内针+手针+梅花针+七星针+平衡针+毫针+揿针) AND (随机对照+随机分组+随机)

**CNKI**

TKA = ('针灸'+'针刺'+'体针'+'电针'+'温针'+'耳埋'+'耳针'+'耳廓针刺术'+'头皮针'+'头针'+'火针'+'燔针'+'腹针'+'眼针'+'腕踝针'+'埋线'+'皮内针'+'手针'+'梅花针'+'七星针'+'平衡针'+'毫针'+'揿针') AND TKA = ('原发性失眠'+'失眠'+'不寐'+'睡眠障碍'+'睡眠起始功能障碍'+'早醒'+'入睡和睡眠失调') AND TKA = ('随机对照'+'随机分组'+'随机')

**Wanfang**

(原发性失眠 OR 失眠 OR 不寐 OR 睡眠障碍 OR 睡眠起始功能障碍 OR 早醒 OR 入睡和睡眠失) AND (针灸 OR 针刺 OR 电针 OR 温针 OR 耳埋 OR 耳针 OR 耳廓针刺术 OR 头皮针 OR 头针 OR 火针 OR 眼针 OR 埋线 OR 皮内针 OR 梅花针 OR 毫针 OR 揿针) AND (随机对照 OR 随机分组 OR 随机)

**CBM**

**#1** "针灸疗法"[不加权:扩展]

**#2** "针灸 OR 针刺 OR 体针 OR 电针 OR 温针 OR 耳埋 OR 耳针] OR 耳廓针刺术 OR 头皮针 OR 头针 OR 火针 OR 燔针 OR 腹针 OR 眼针 OR 腕踝针 OR 埋线 OR 皮内针 OR 手针 OR 梅花针 OR 七星针 OR 平衡针 OR 毫针 OR 揿针" [常用字段:智能]

**#3** #1 OR #2

**#4** "入睡和睡眠障碍"[不加权:扩展]

**#5** "原发性失眠 OR 失眠 OR 不寐 OR 睡眠障碍 OR 睡眠起始功能障碍 OR 早醒 OR 入睡和睡眠失调" [常用字段:智能]

**#6** #4 OR #5

**#7** "随机 OR 随机分组 OR " [常用字段:智能]

**#8** #3 AND #6 AND #7
