# Supplementary material for: Comparative effectiveness and safety of acupuncture treatments for primary insomnia: a systematic review and network meta-analysis of randomized trial
Source: Front Neurol. 2026 Mar 3;17:1750474. doi: 10.3389/fneur.2026.1750474 (PMC12992266; doi:10.3389/fneur.2026.1750474)

**Appendix C:** SUCRA

(1)PSQI 4:


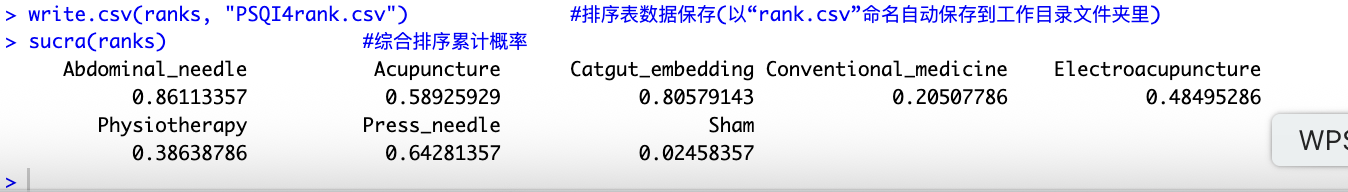


(2)PSQI Maximum Time:


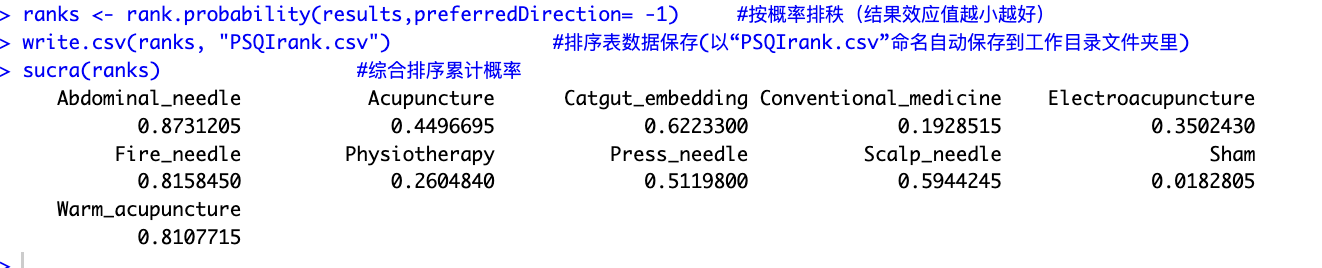


(3)Anxiety Score:


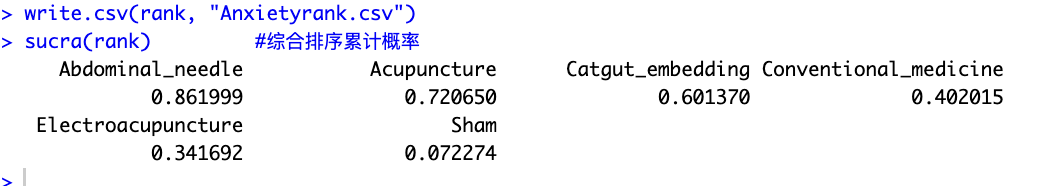


1. Depression Score:


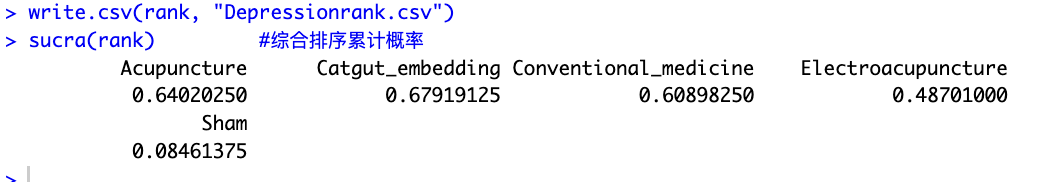


1. TCM Syndrome Score:


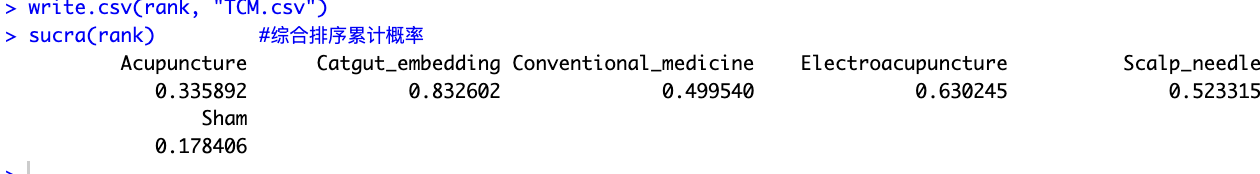


1. Clinical Effective Rate:


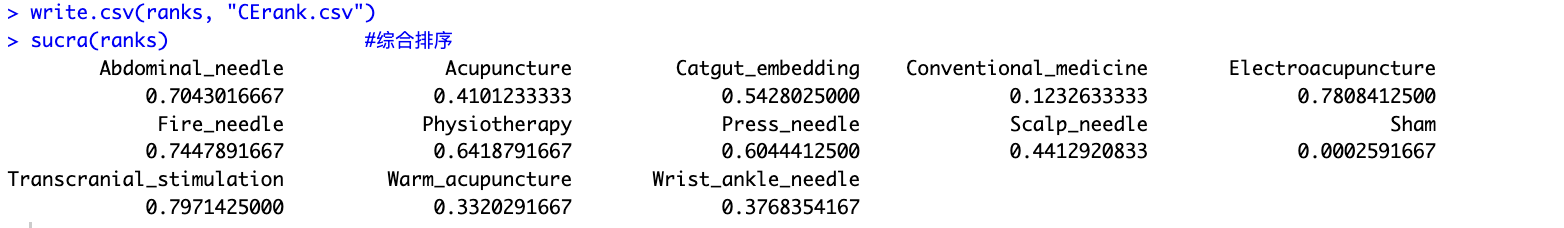


(7)Adverse Event Rate:


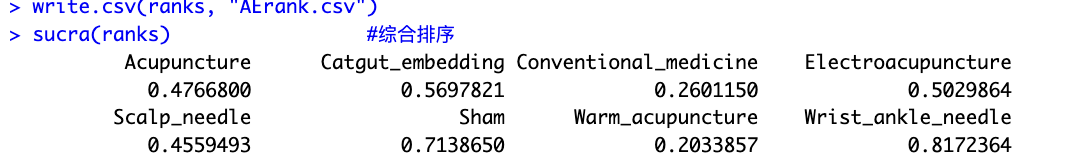

Supplement: Supplementary file 2 [file Table_2.DOCX]
