# Supplementary material for: Comparative effectiveness and safety of acupuncture treatments for primary insomnia: a systematic review and network meta-analysis of randomized trial
Source: Front Neurol. 2026 Mar 3;17:1750474. doi: 10.3389/fneur.2026.1750474 (PMC12992266; doi:10.3389/fneur.2026.1750474)

**Appendix E:** Heterogeneity test

# Heterogeneity test

1. PSQI within 4 Weeks


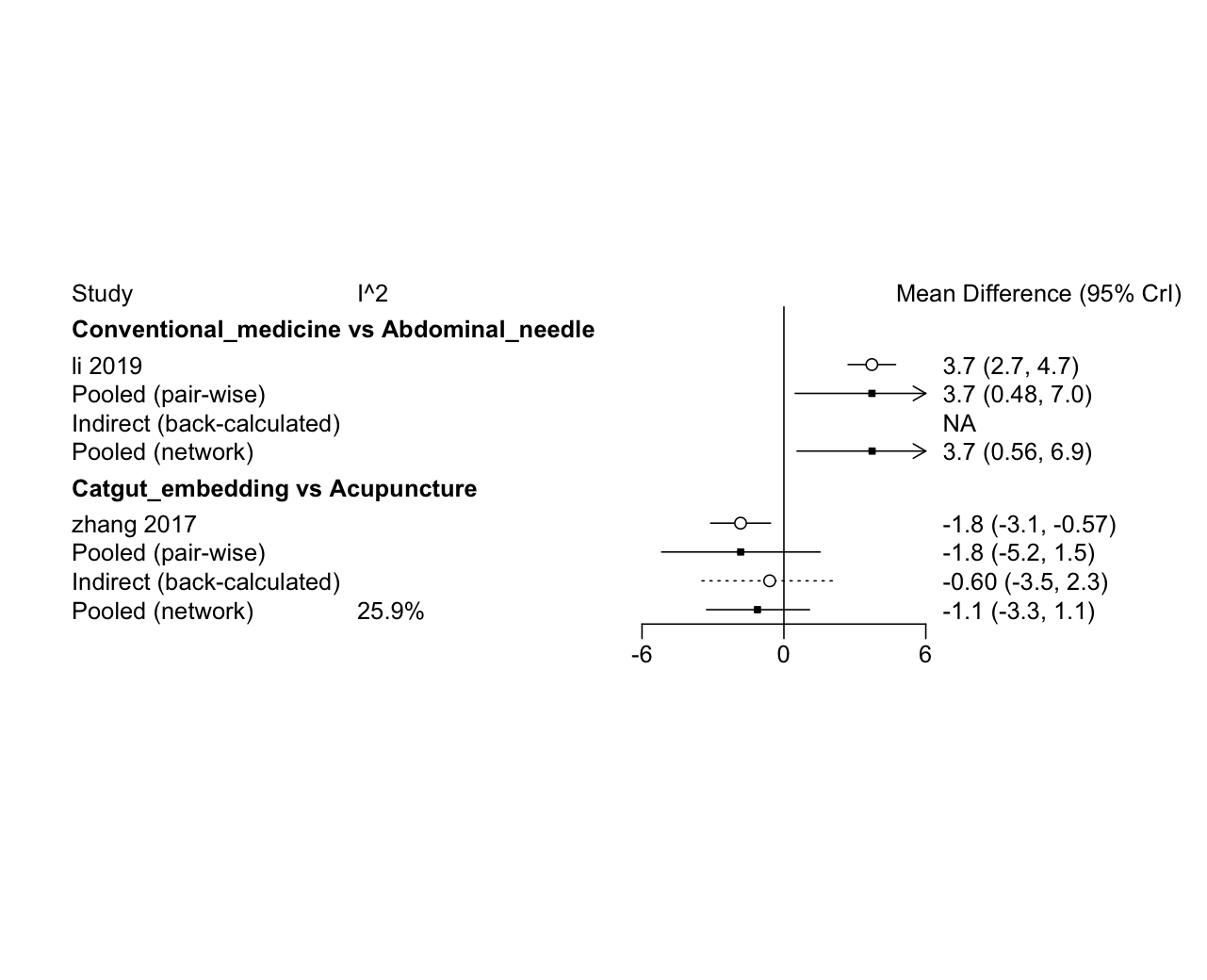

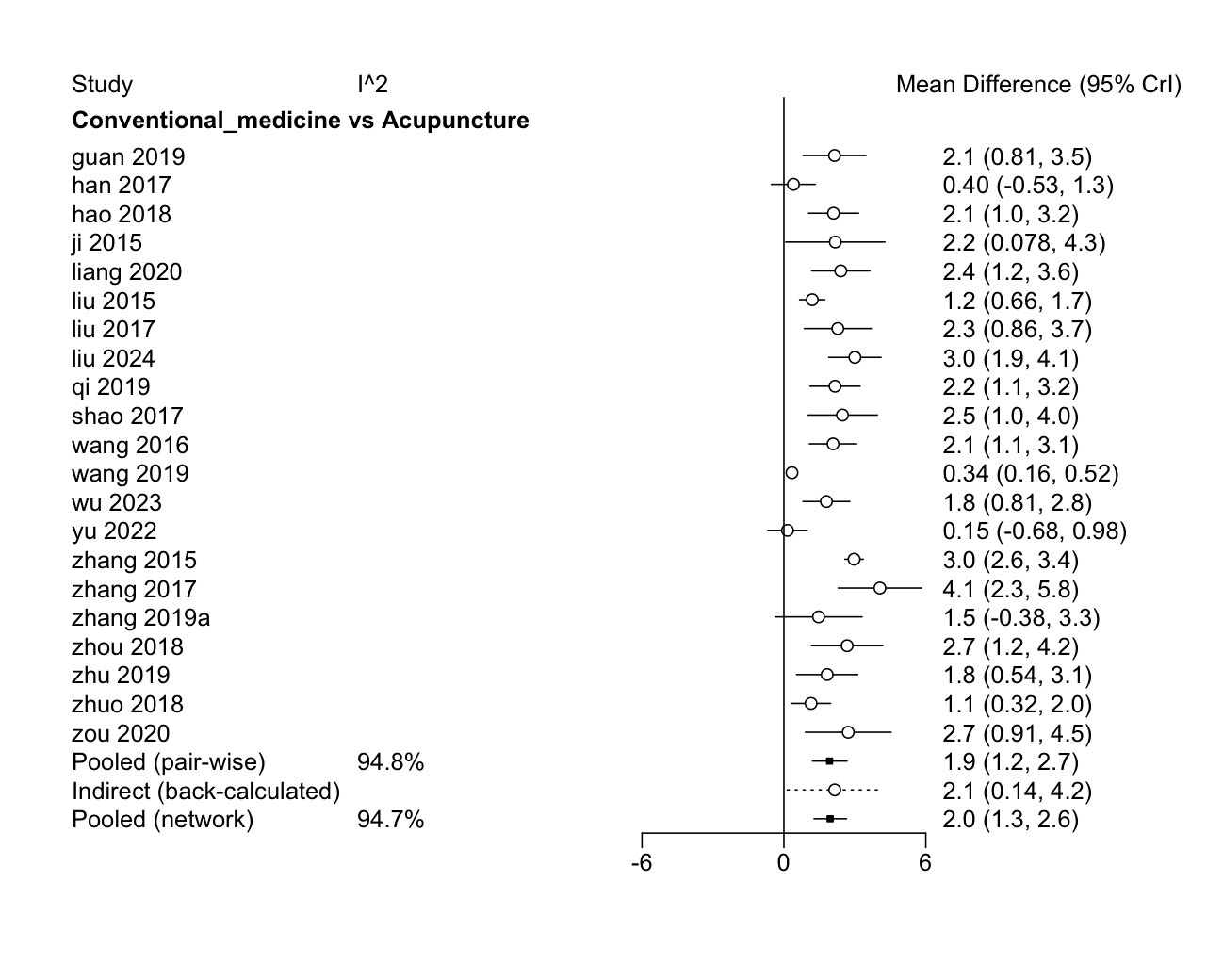

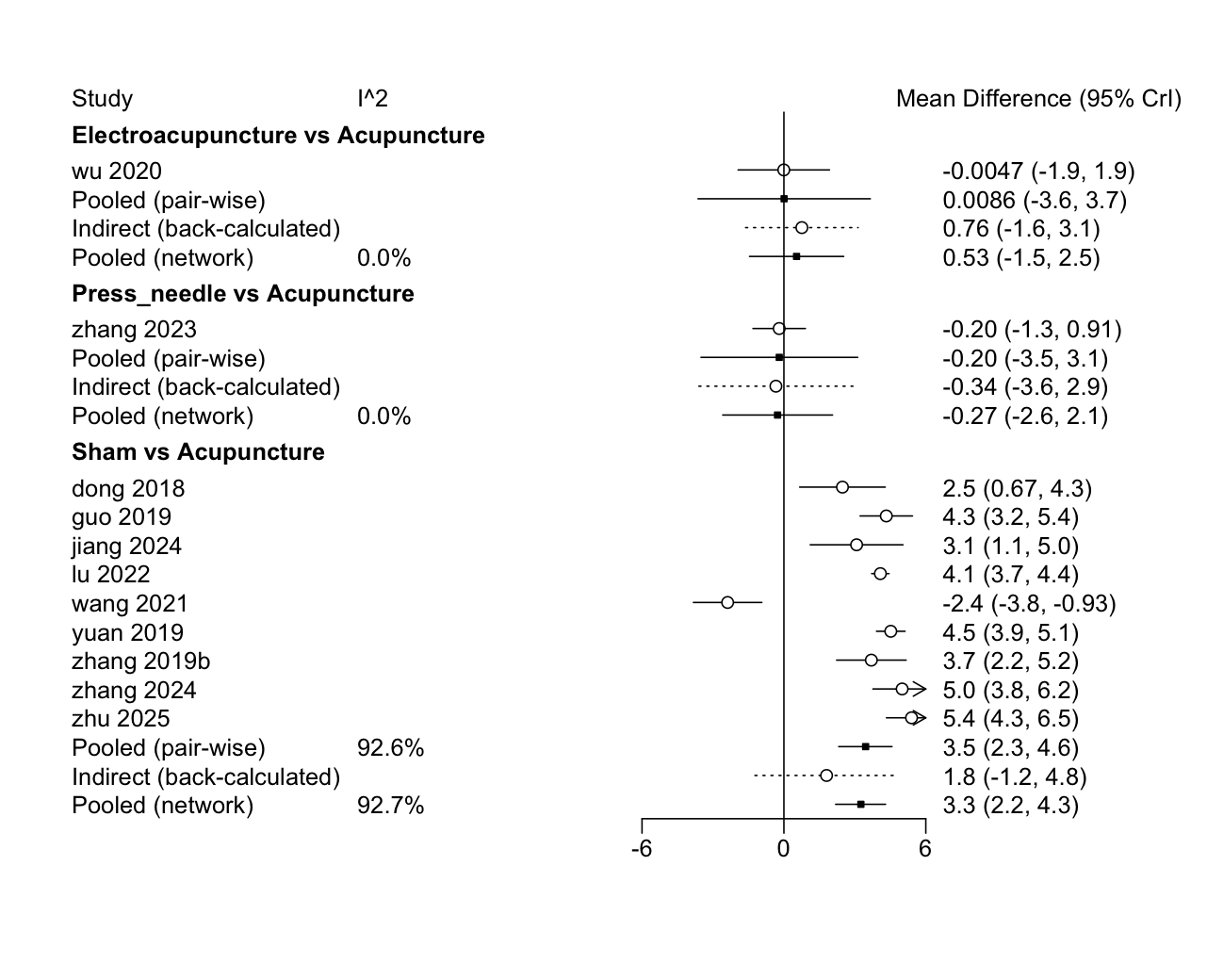

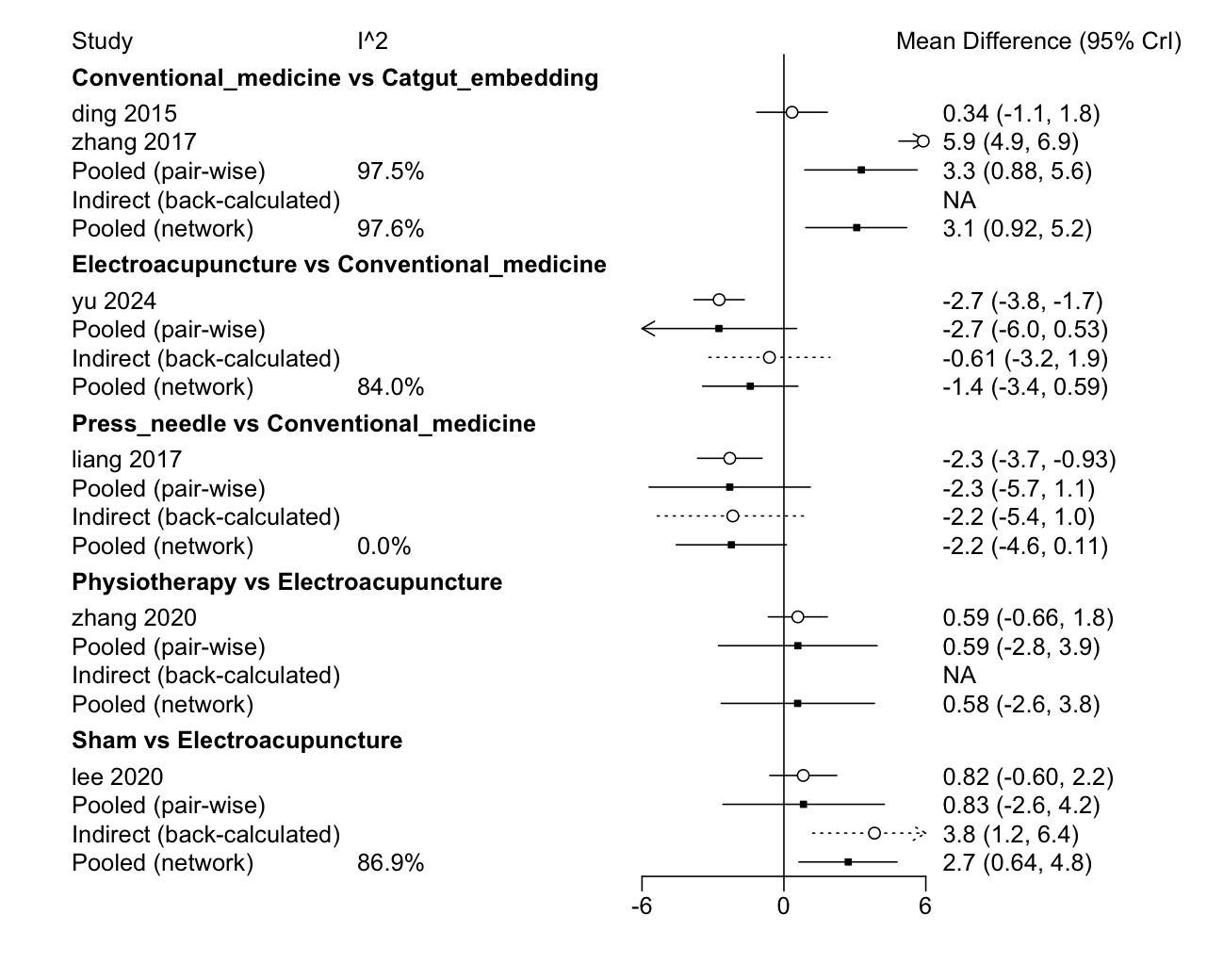


1. Long-Term Effects on PSQI


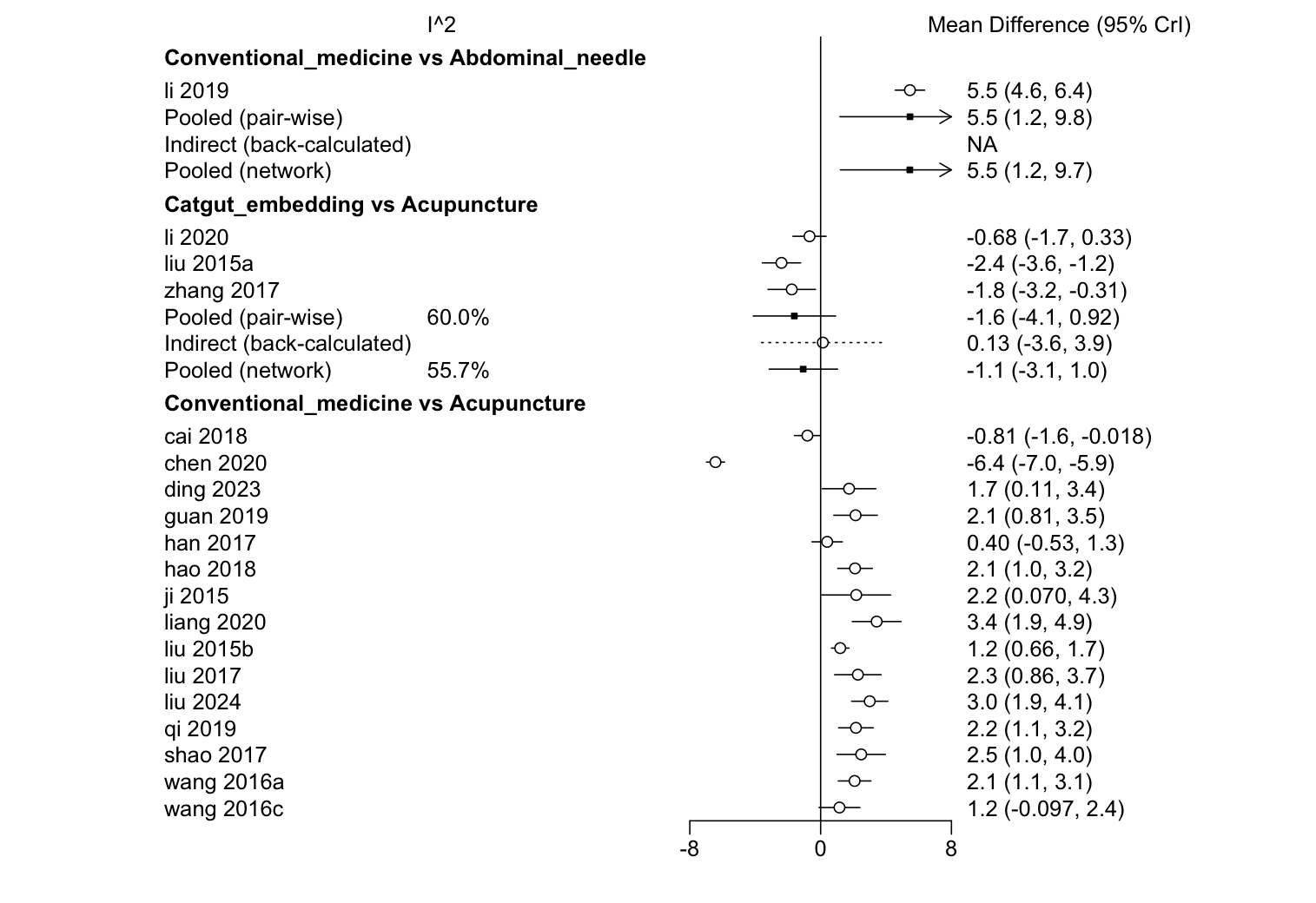

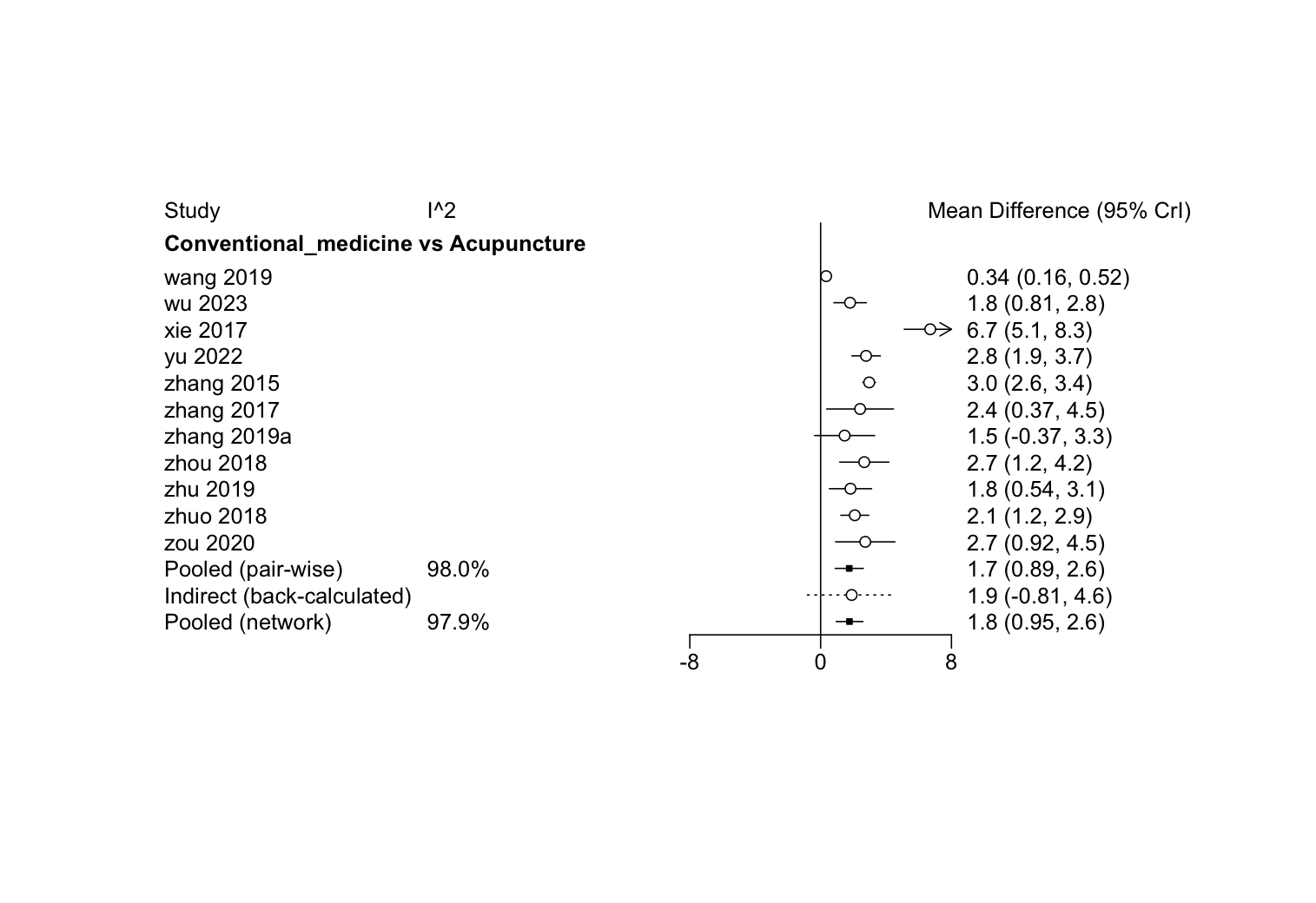

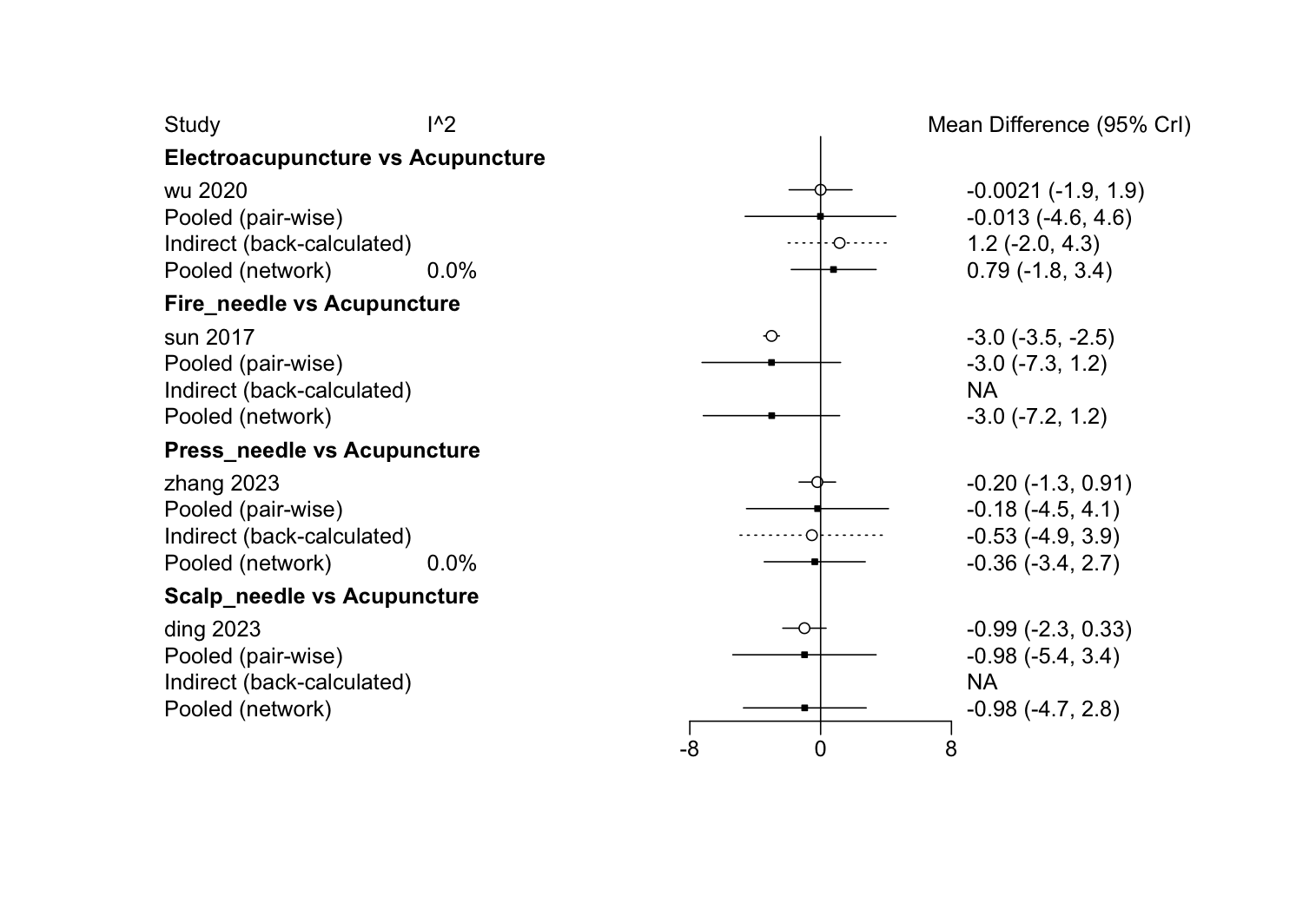

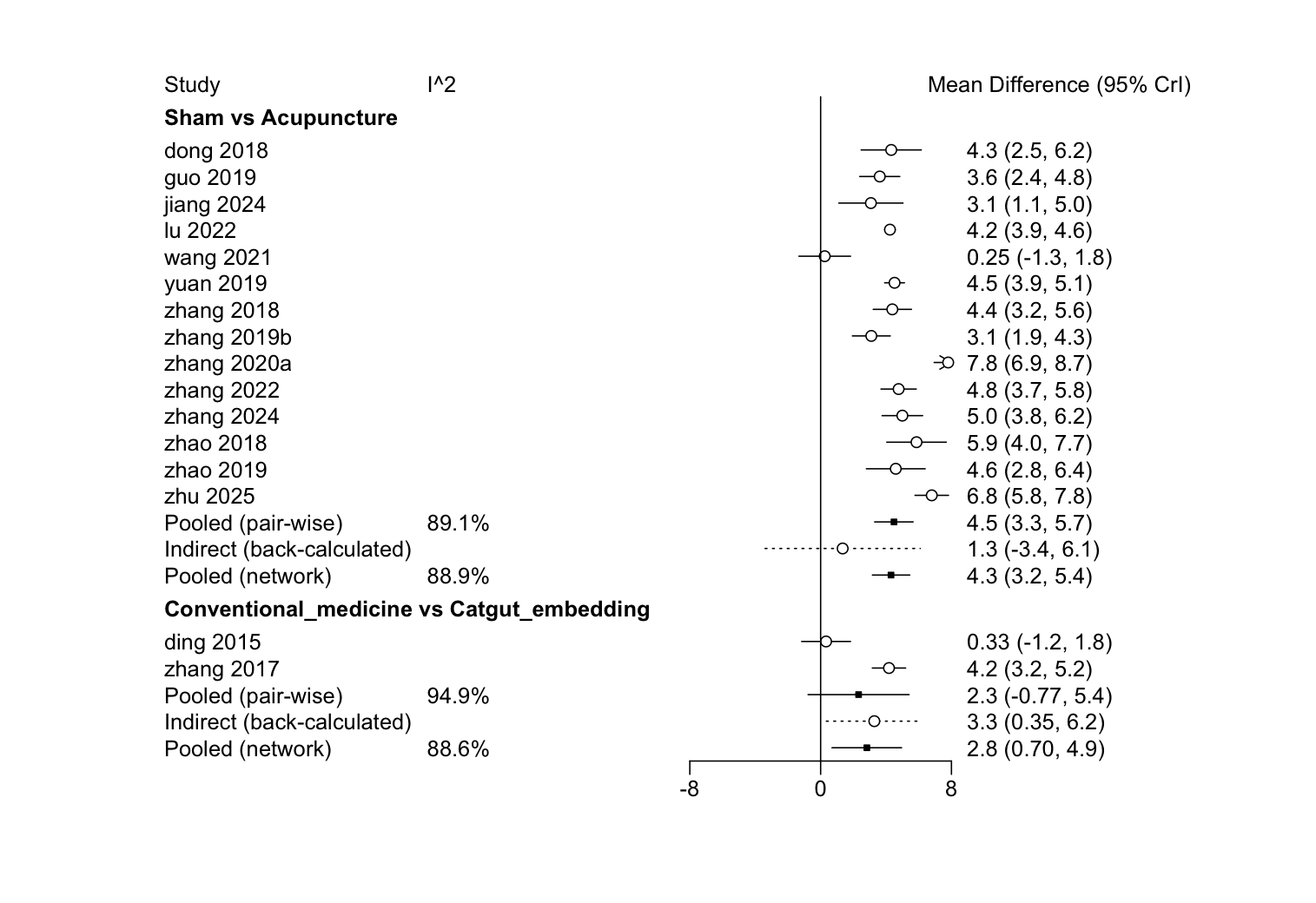

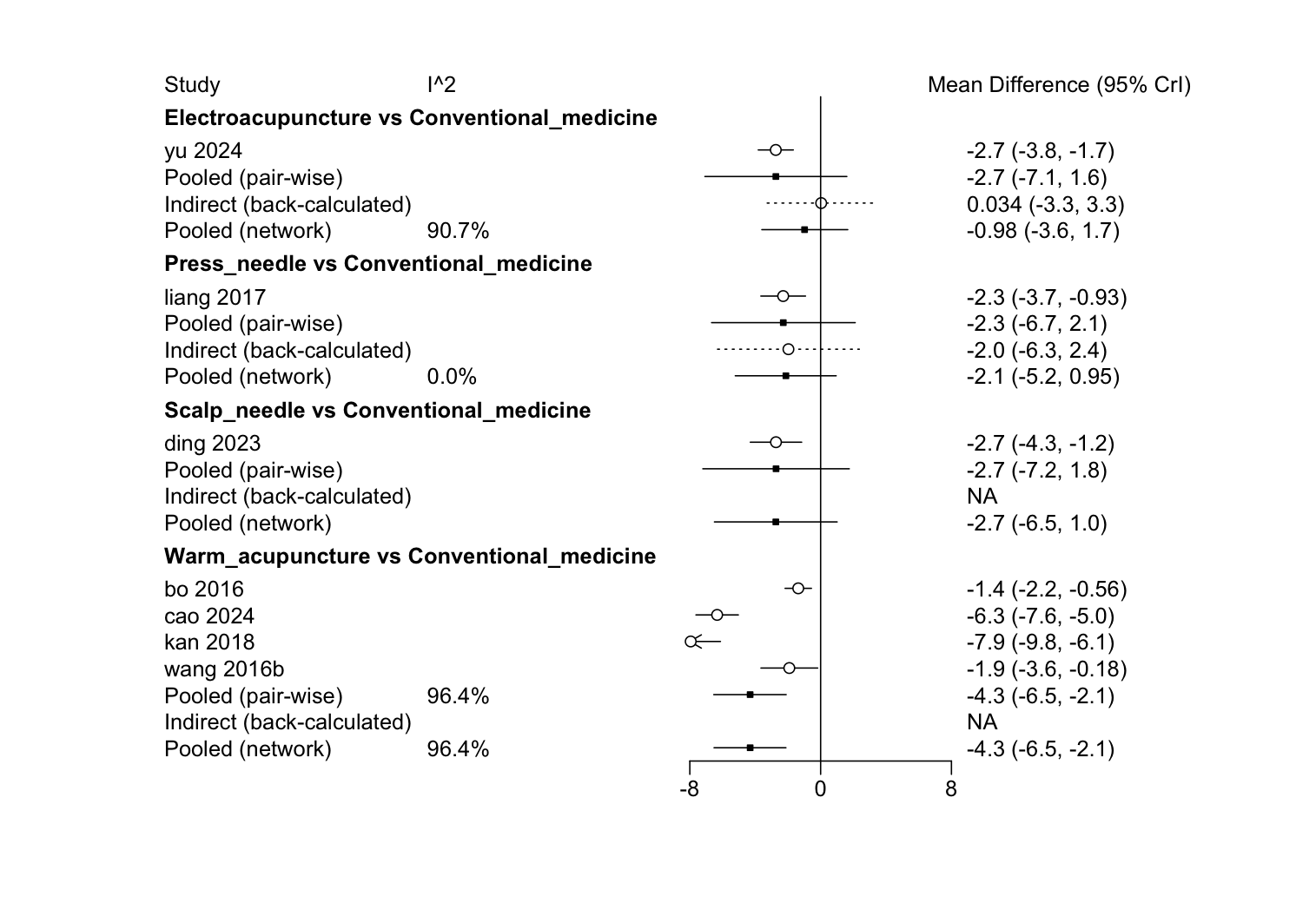

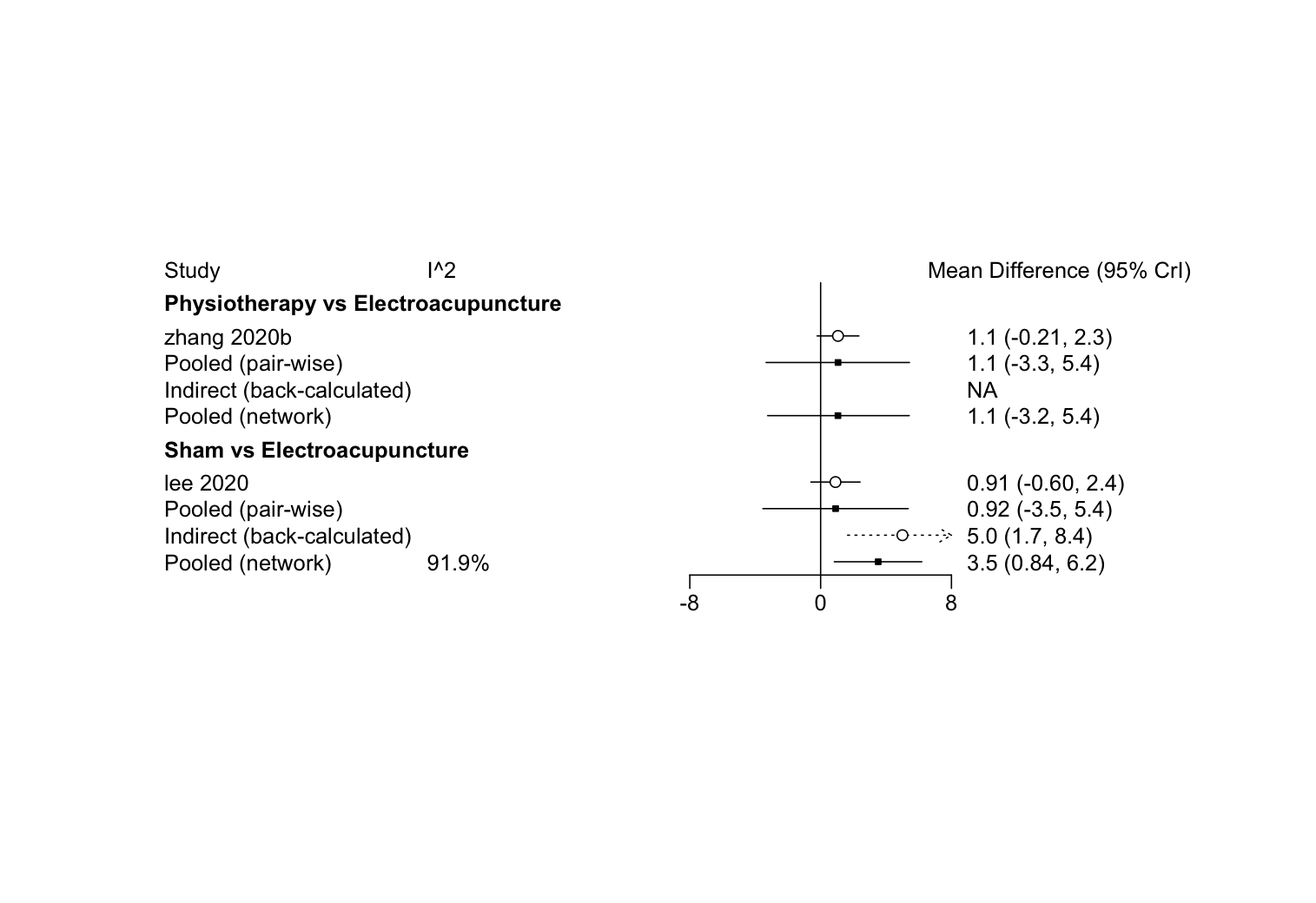


1. Anxiety Scores


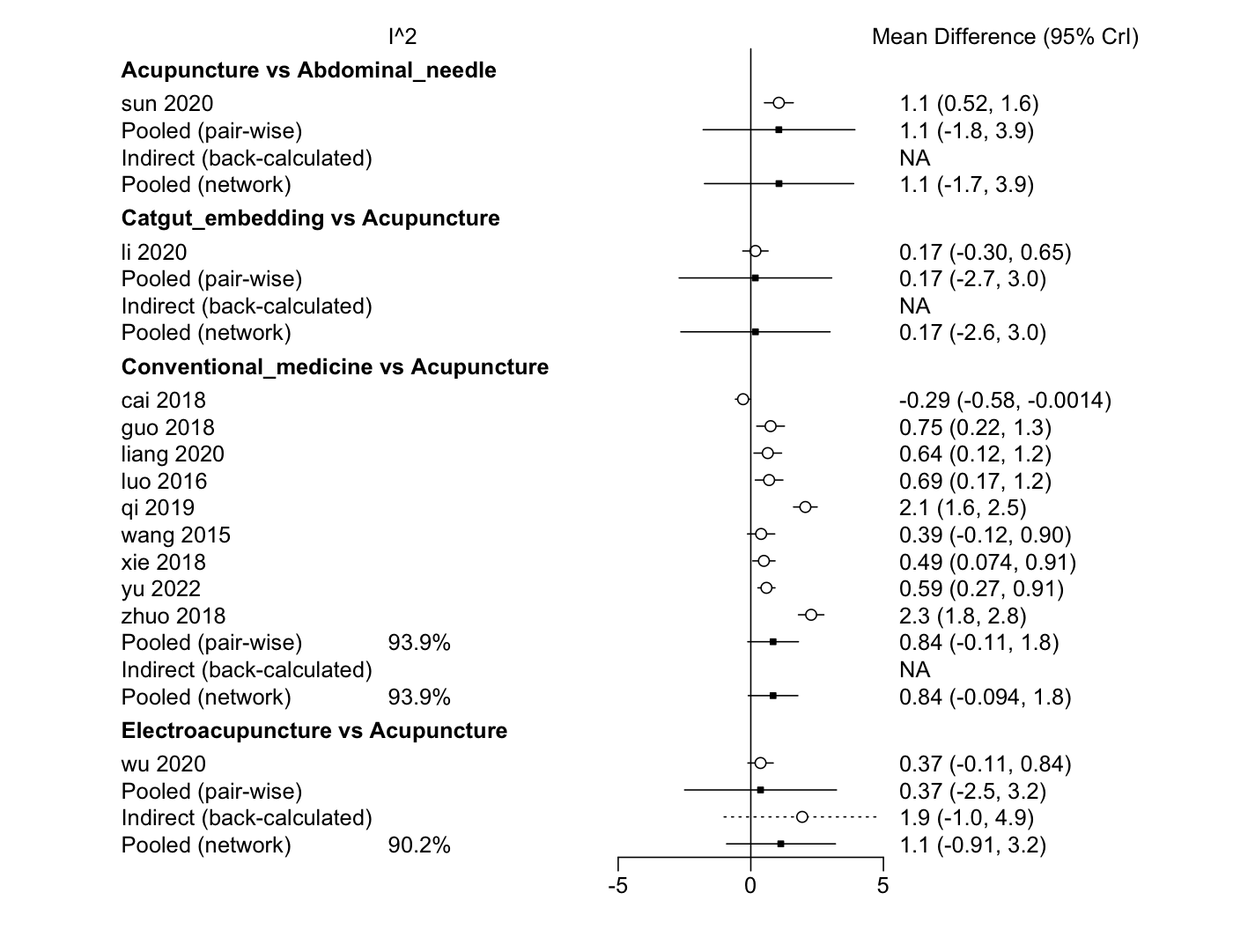

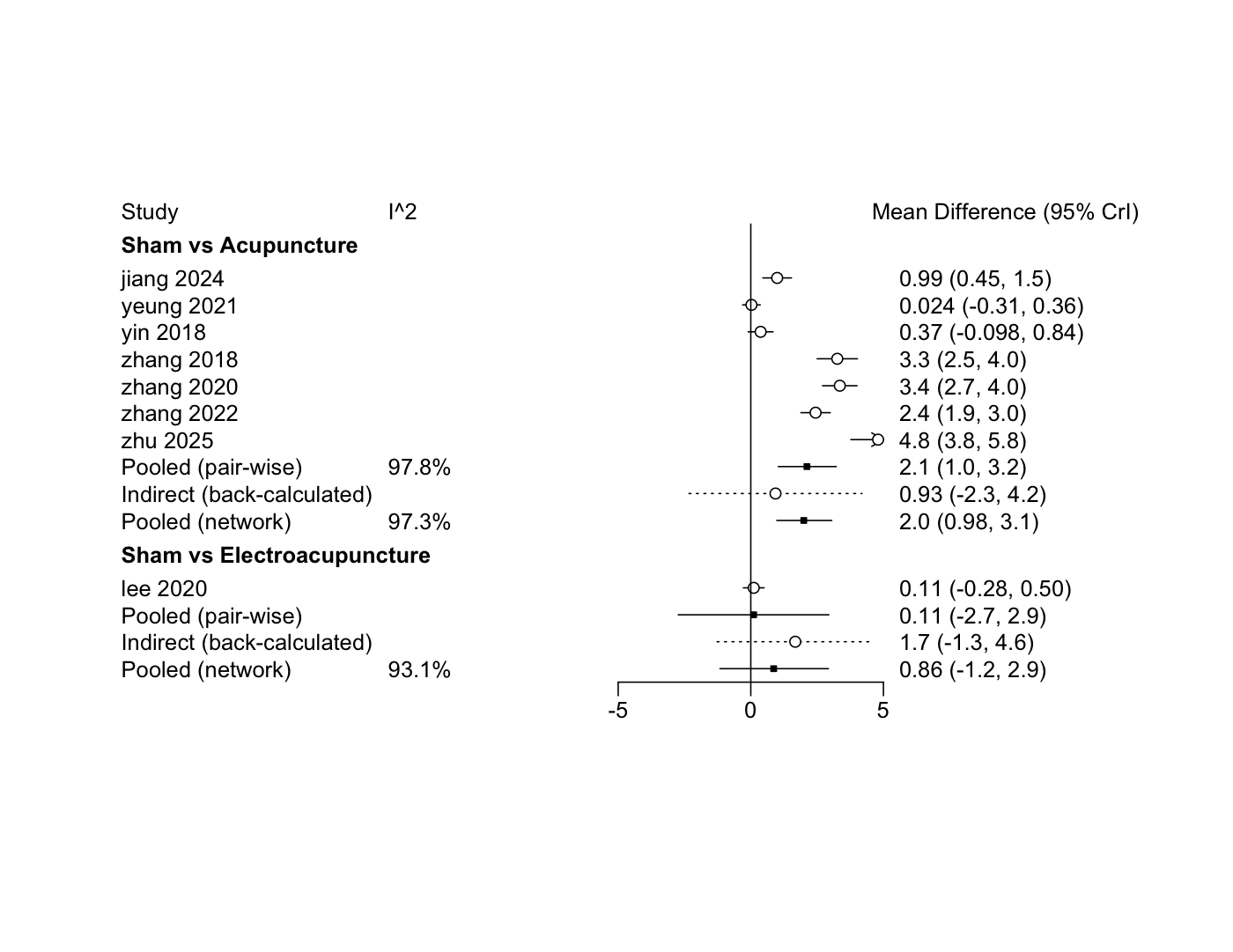


1. Depression Scores


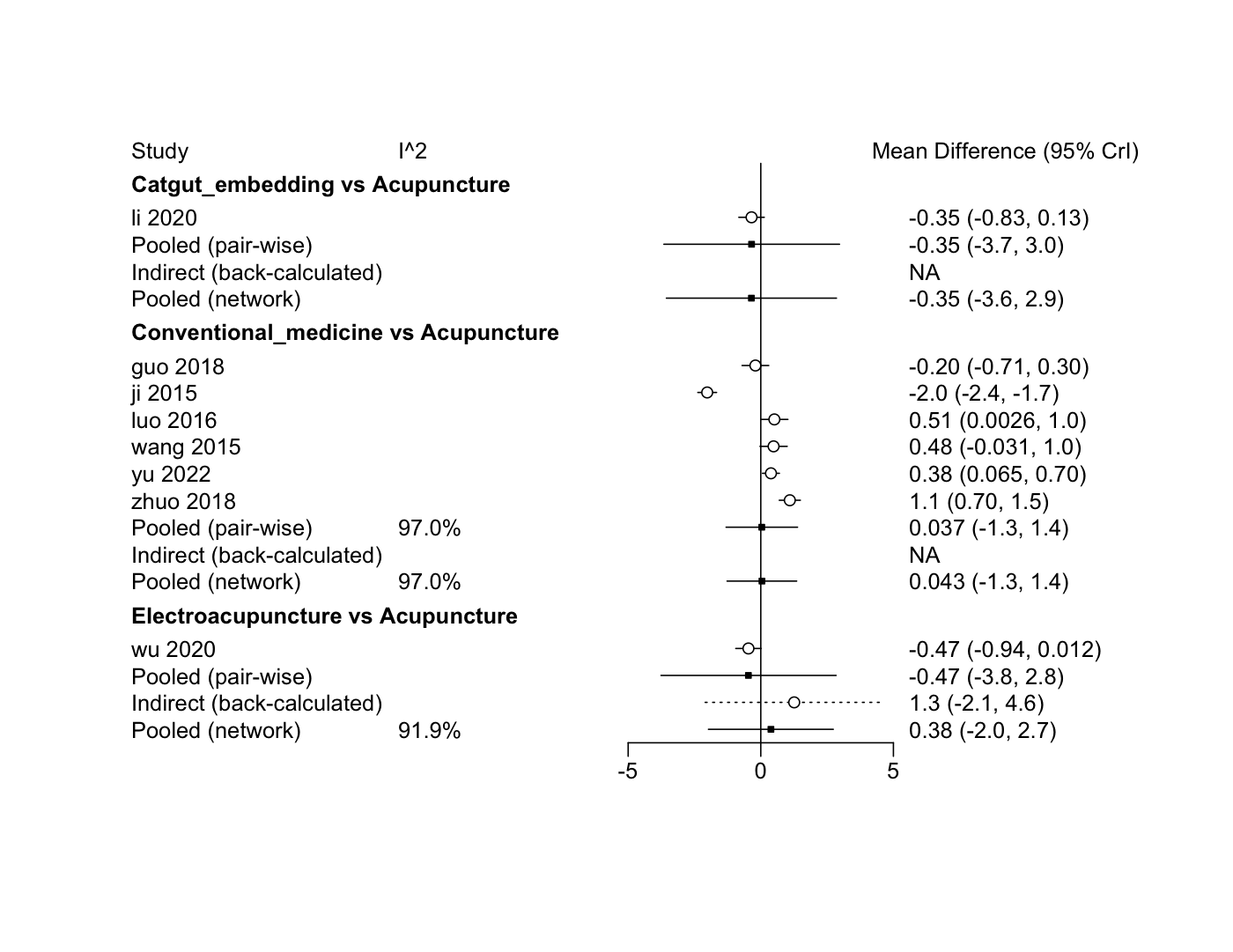

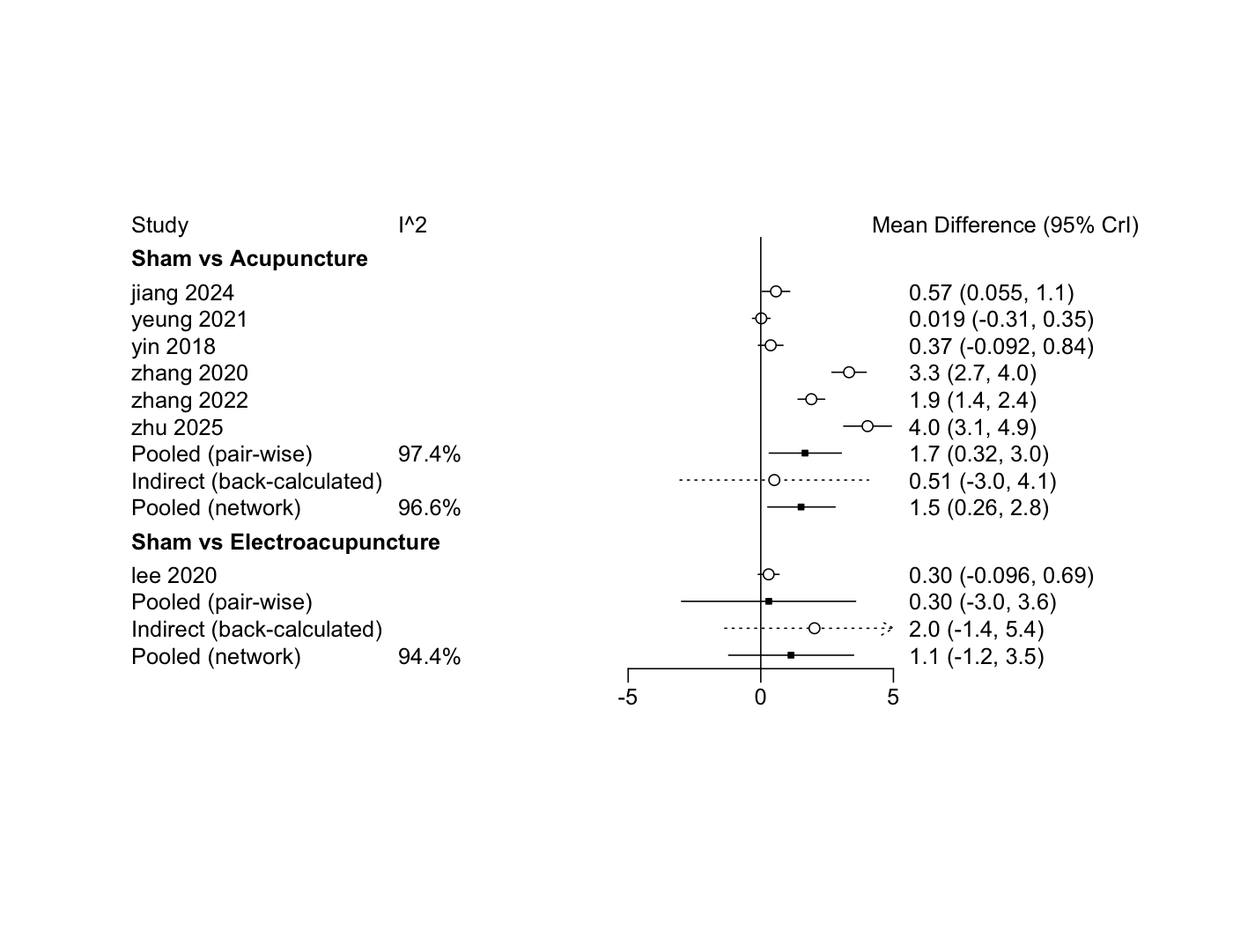


1. TCM Syndrome Scores


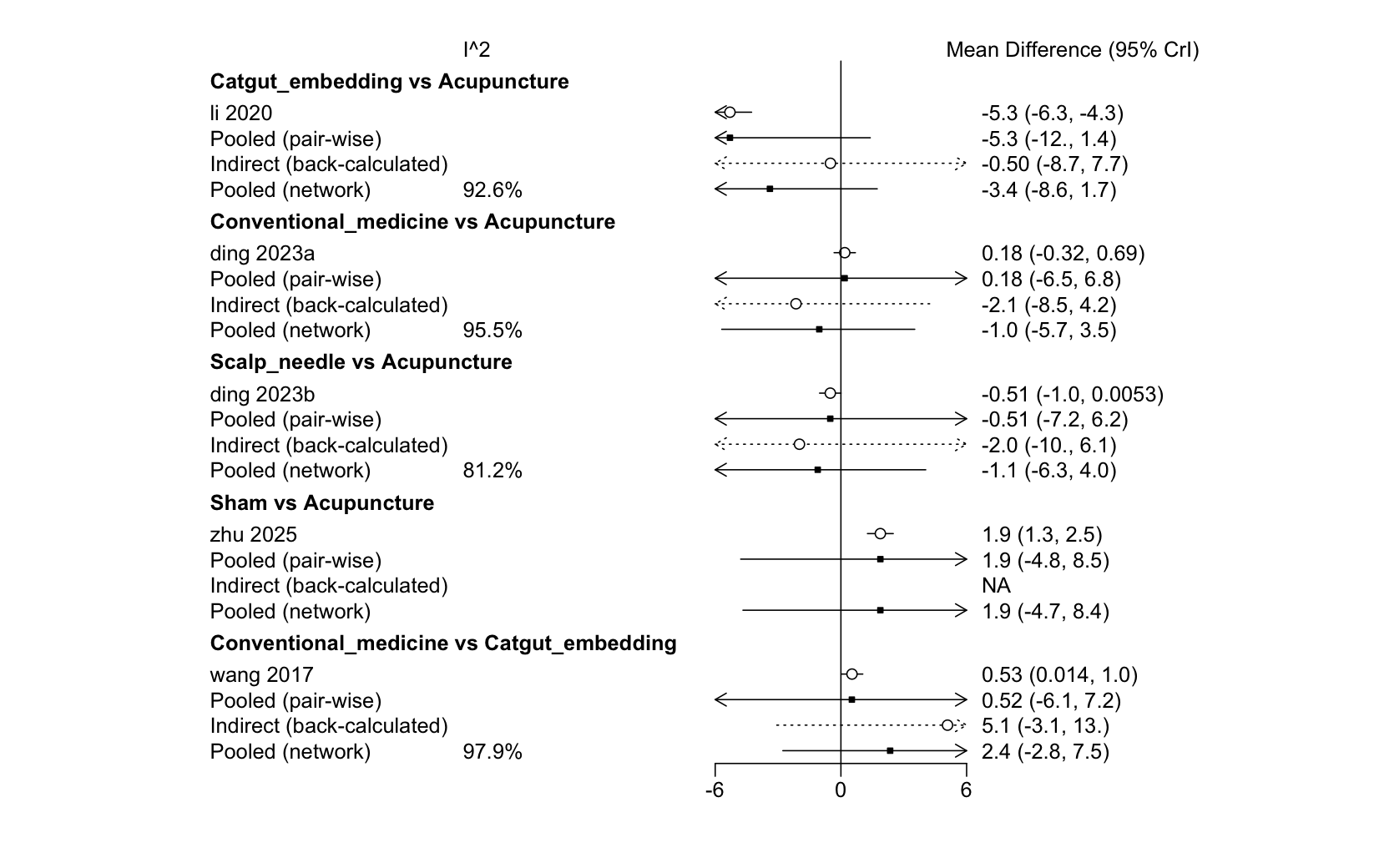

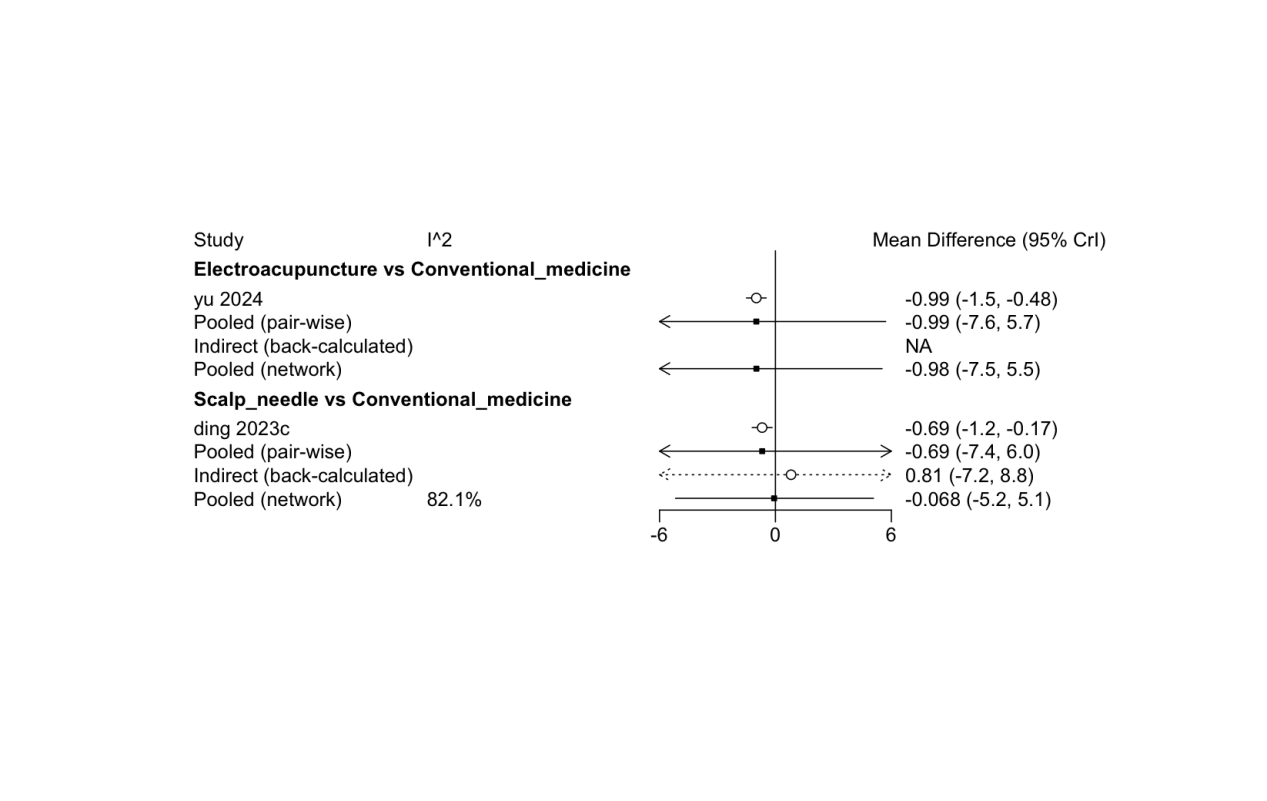


1. Clinical Efficacy Rates


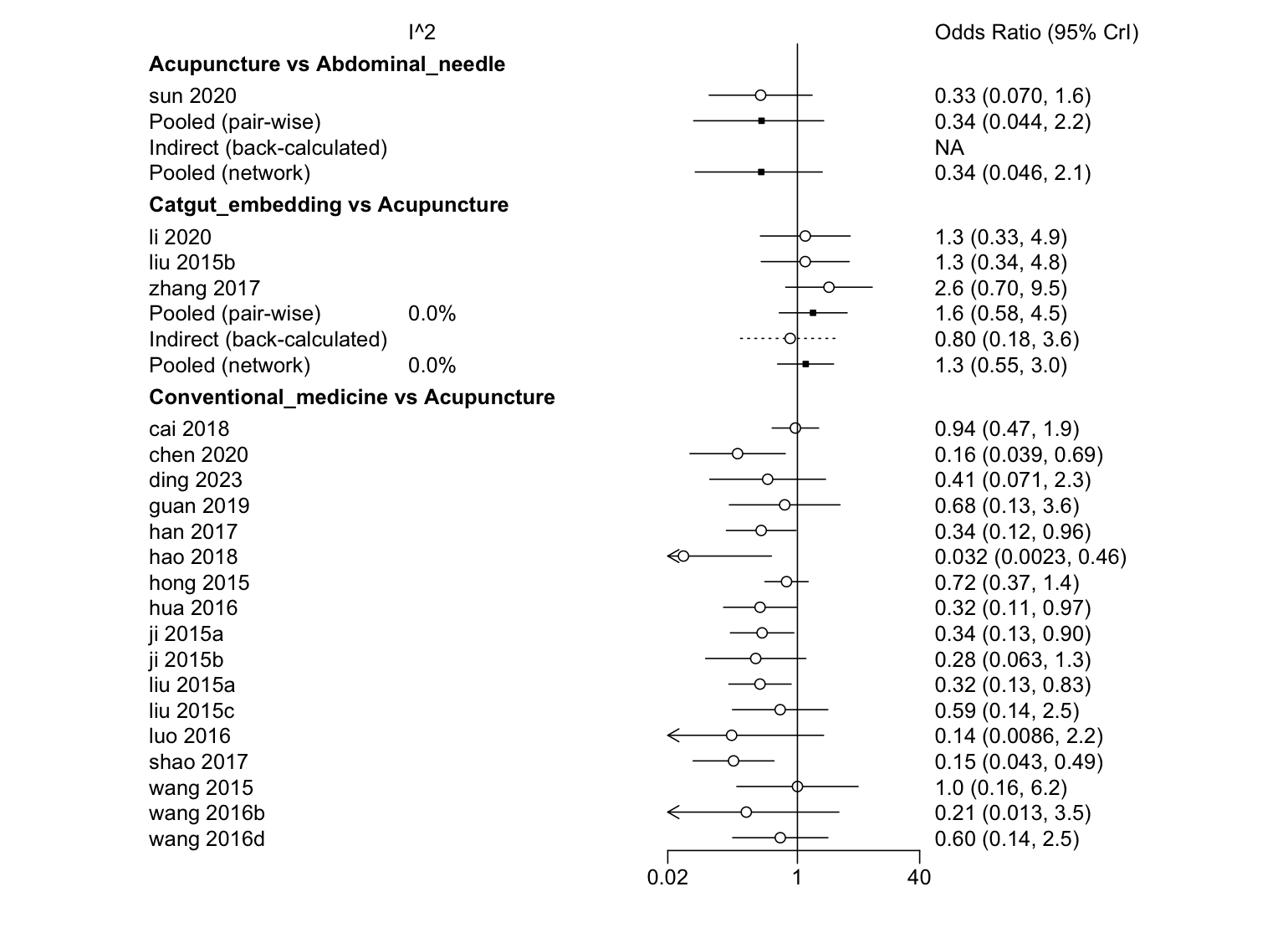

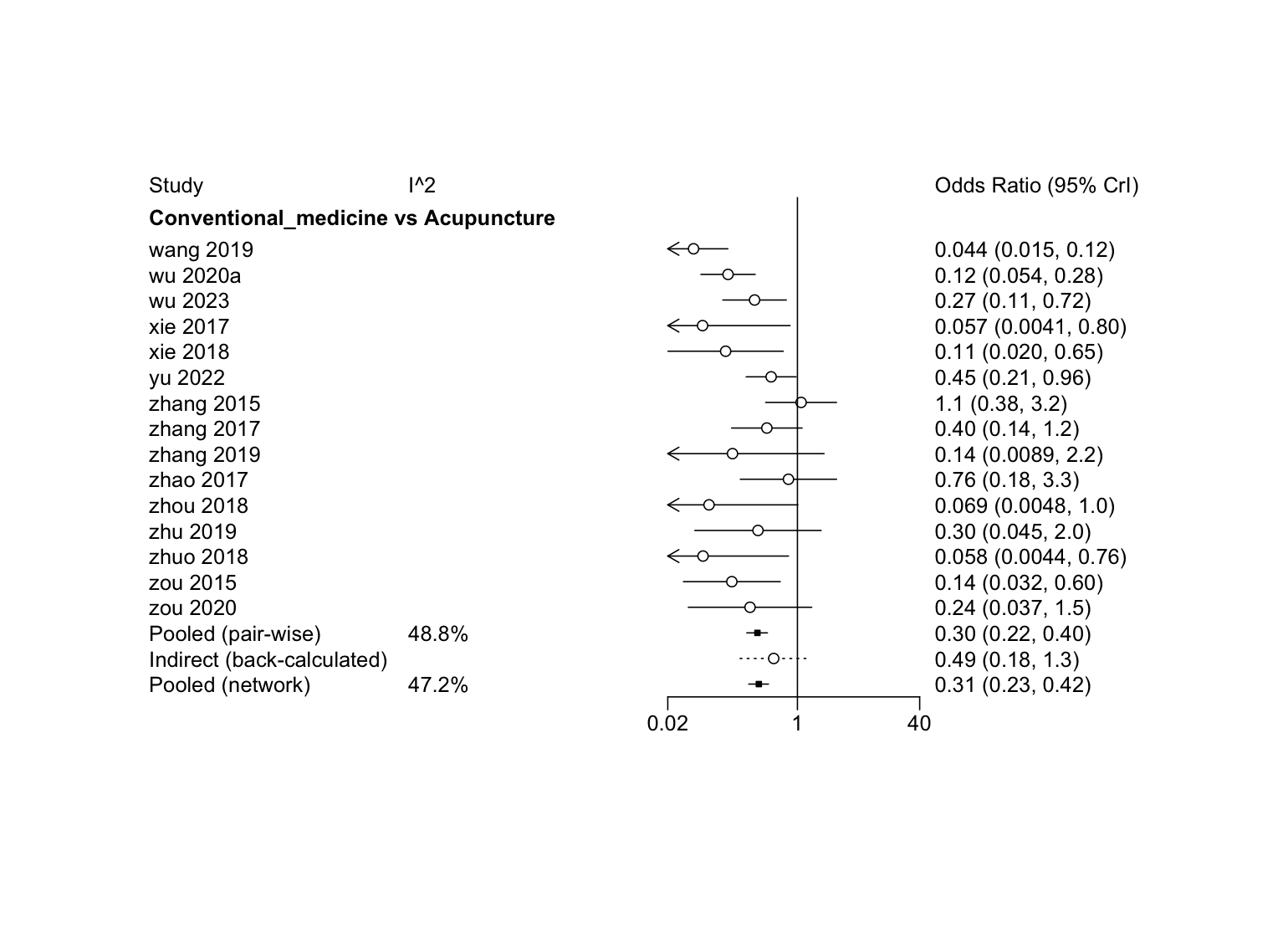

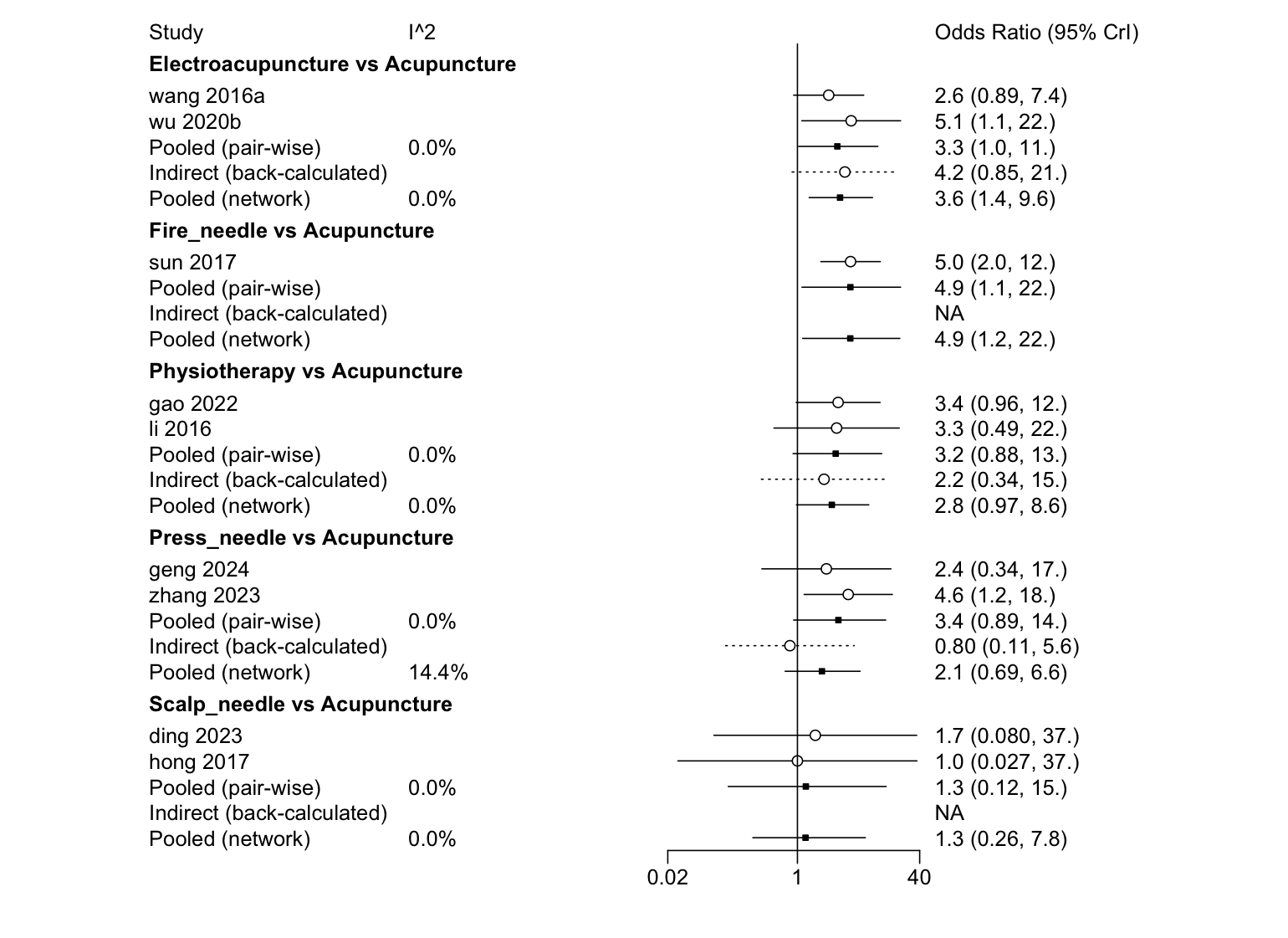

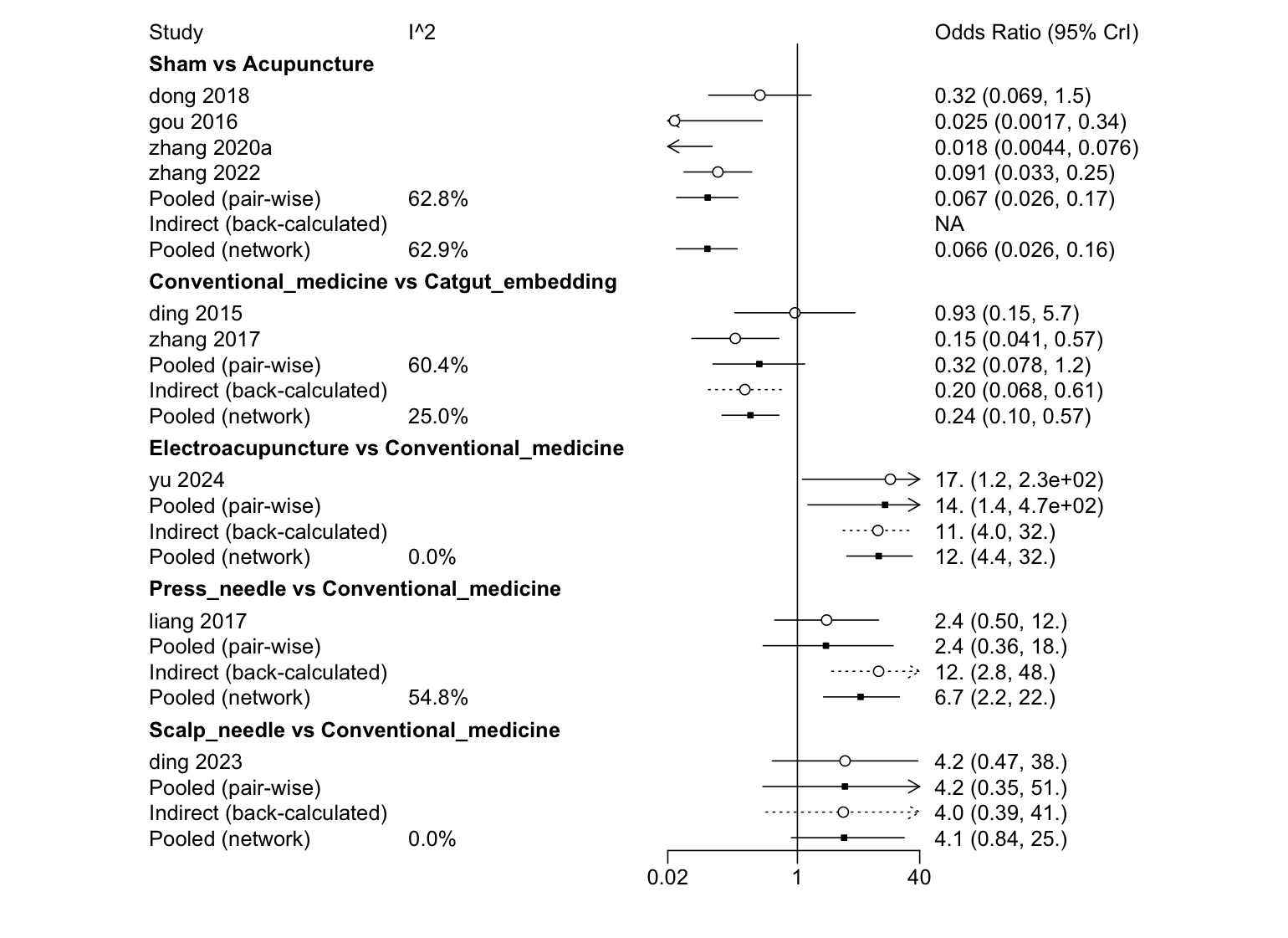

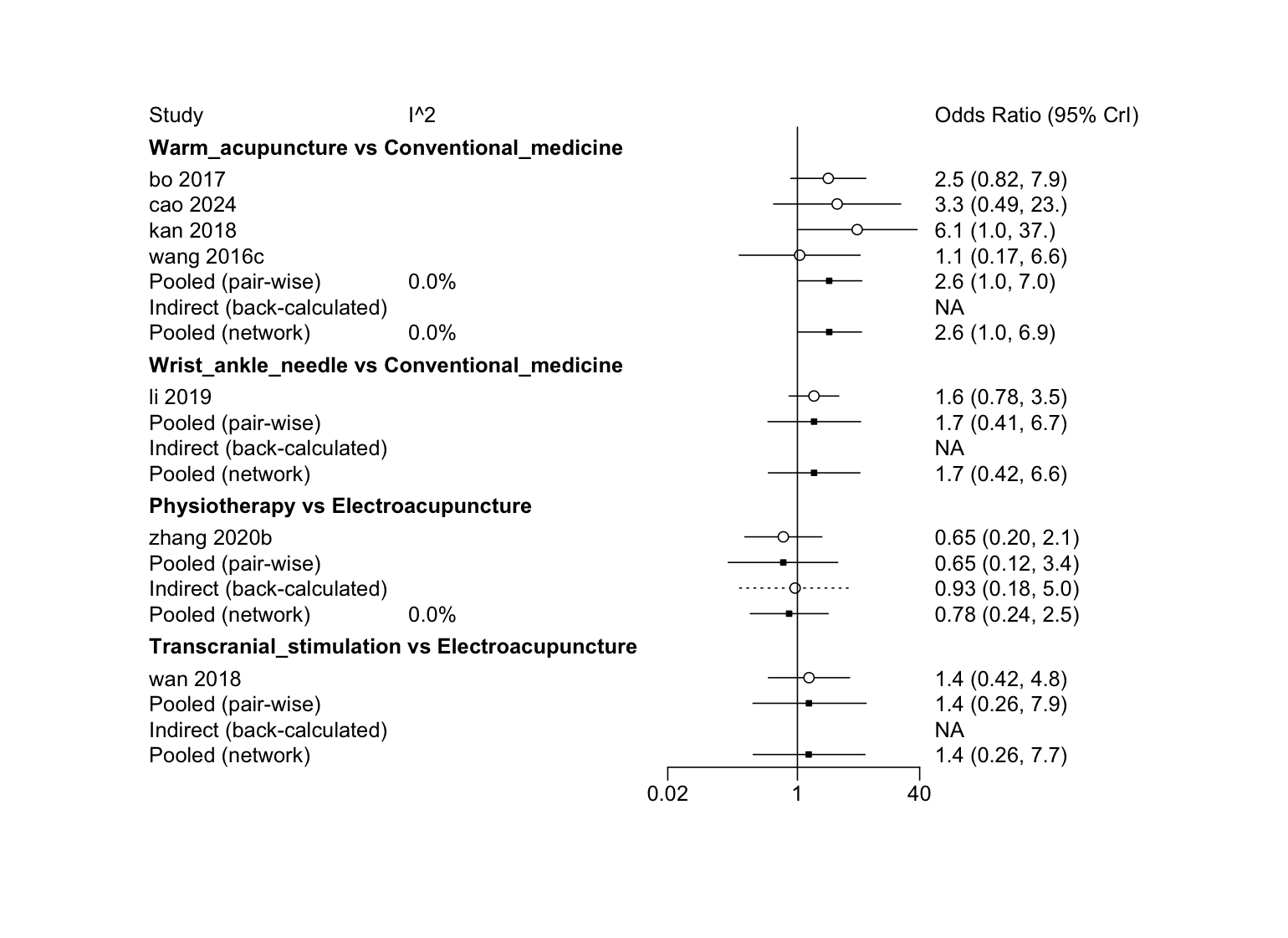


1. Adverse Events


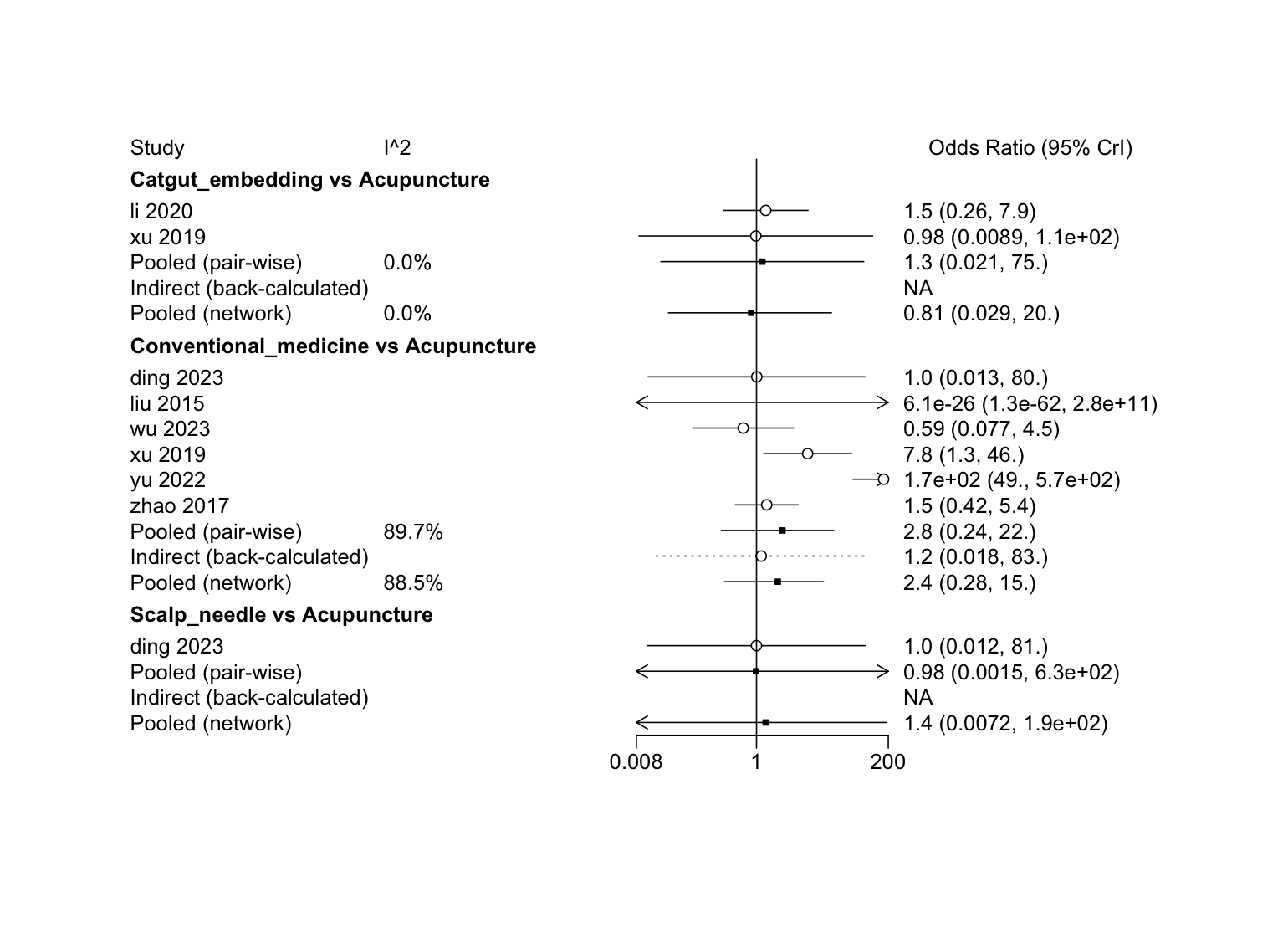

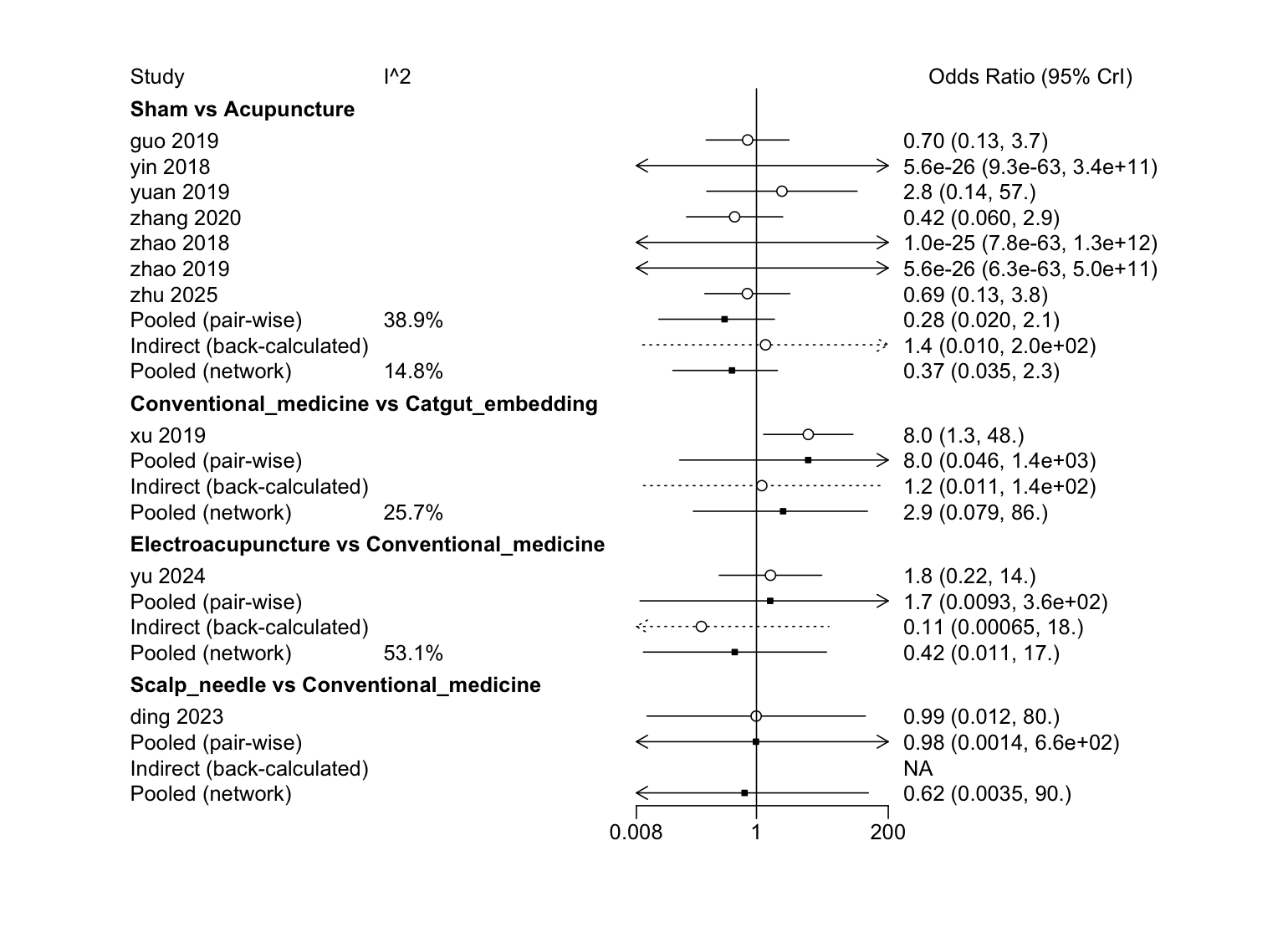

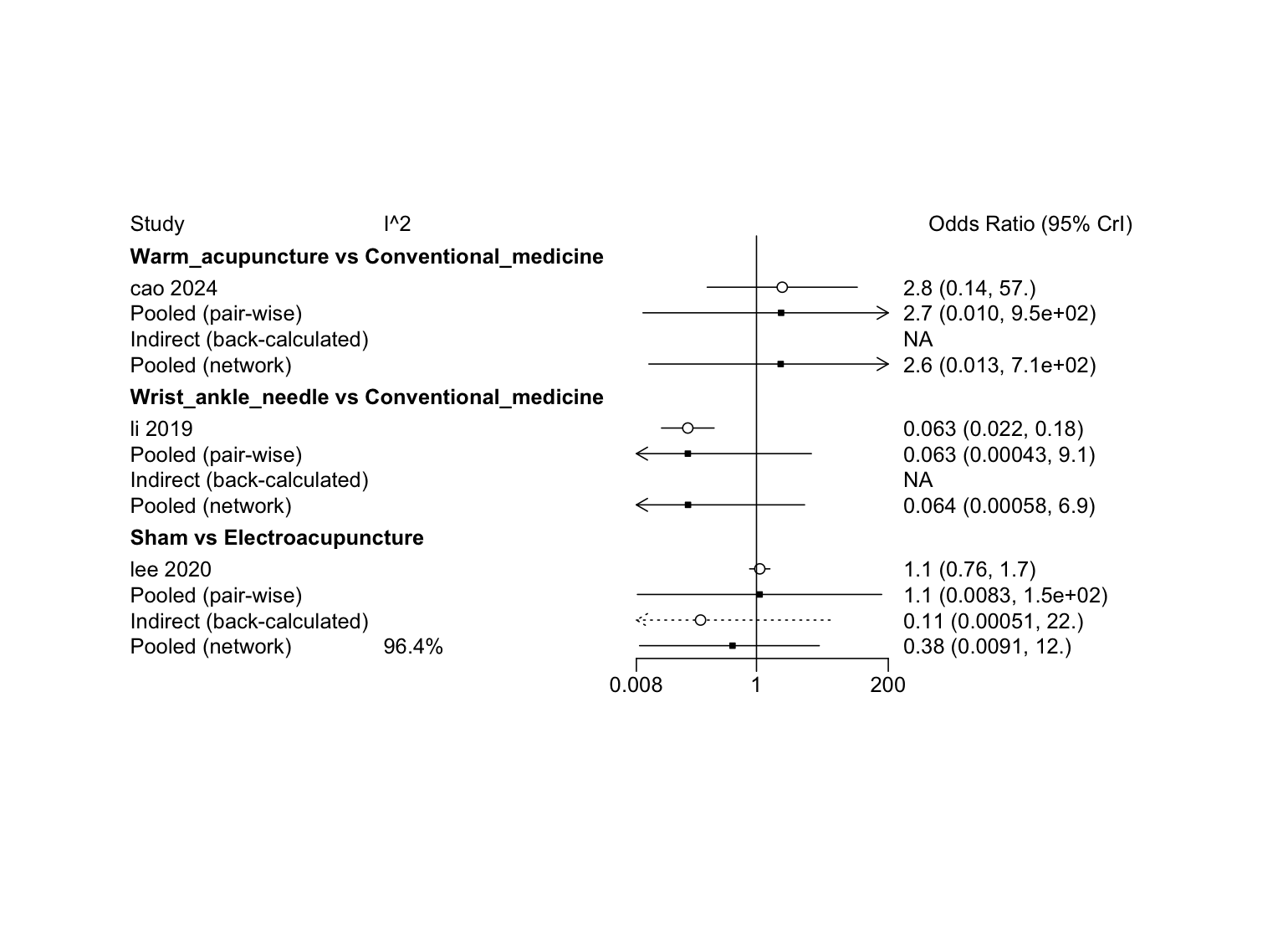

Supplement: Supplementary file 4 [file Table_4.DOCX]
