# Supplementary material for: Comparative effectiveness and safety of acupuncture treatments for primary insomnia: a systematic review and network meta-analysis of randomized trial
Source: Front Neurol. 2026 Mar 3;17:1750474. doi: 10.3389/fneur.2026.1750474 (PMC12992266; doi:10.3389/fneur.2026.1750474)

**Appendix F:** Funnel plot

(1)PSQI 4:


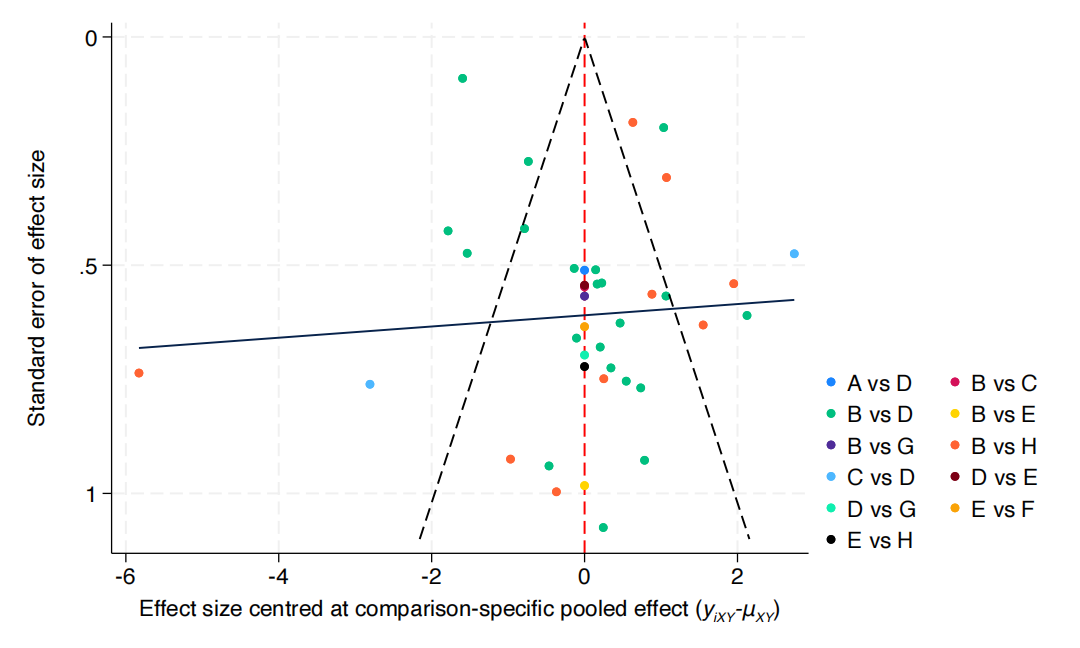


(2)PSQI Maximum Time:


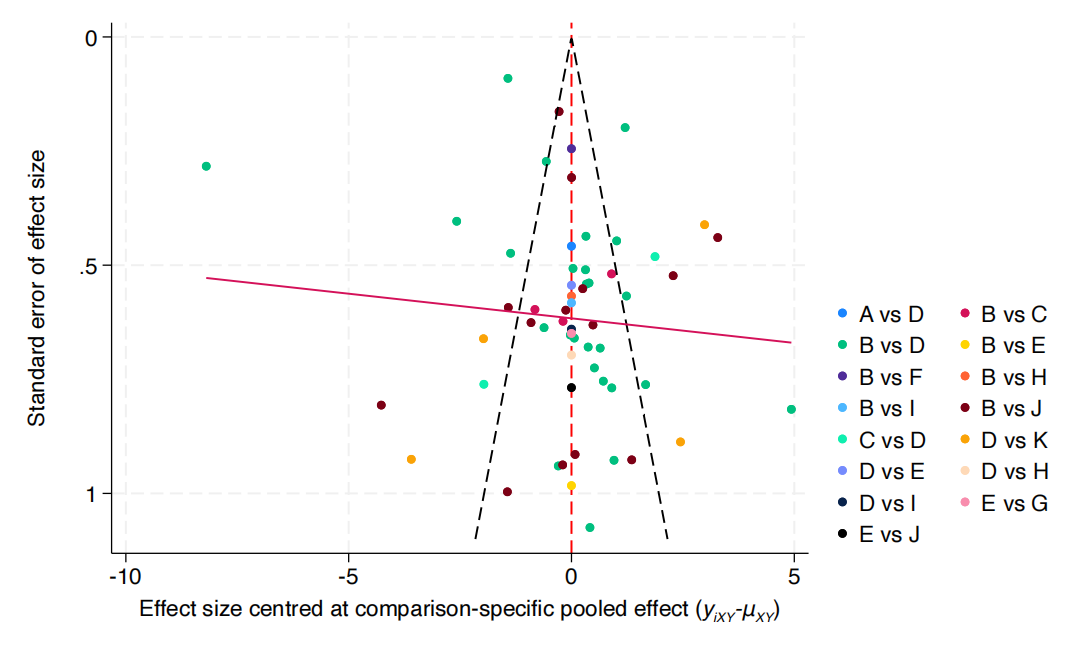


(3)Anxiety Score:


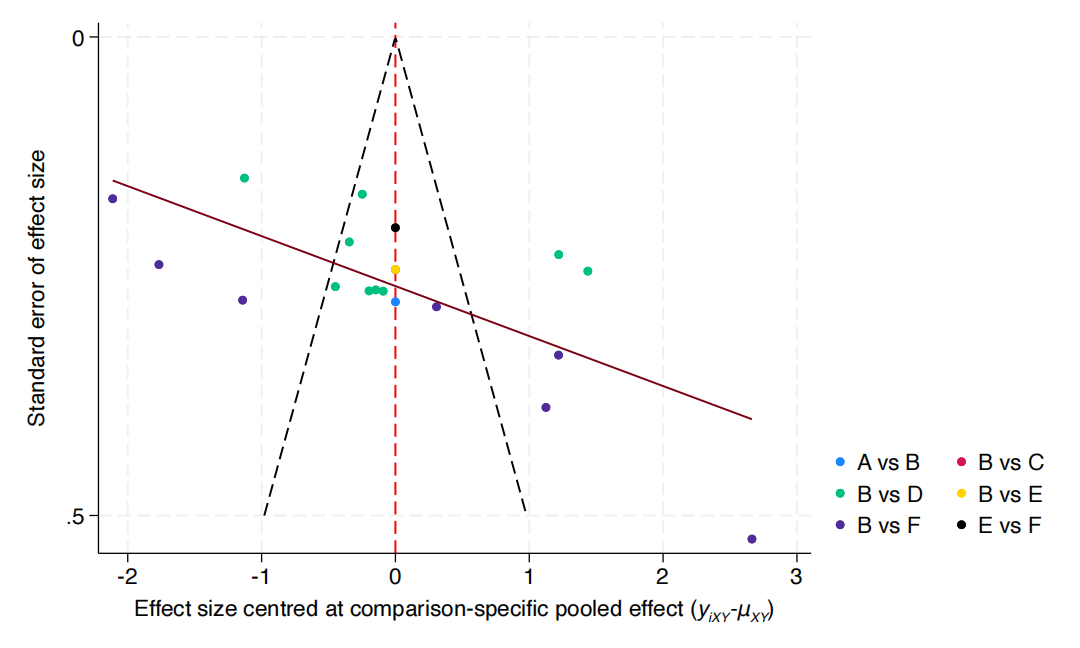


(4)Depression Score:


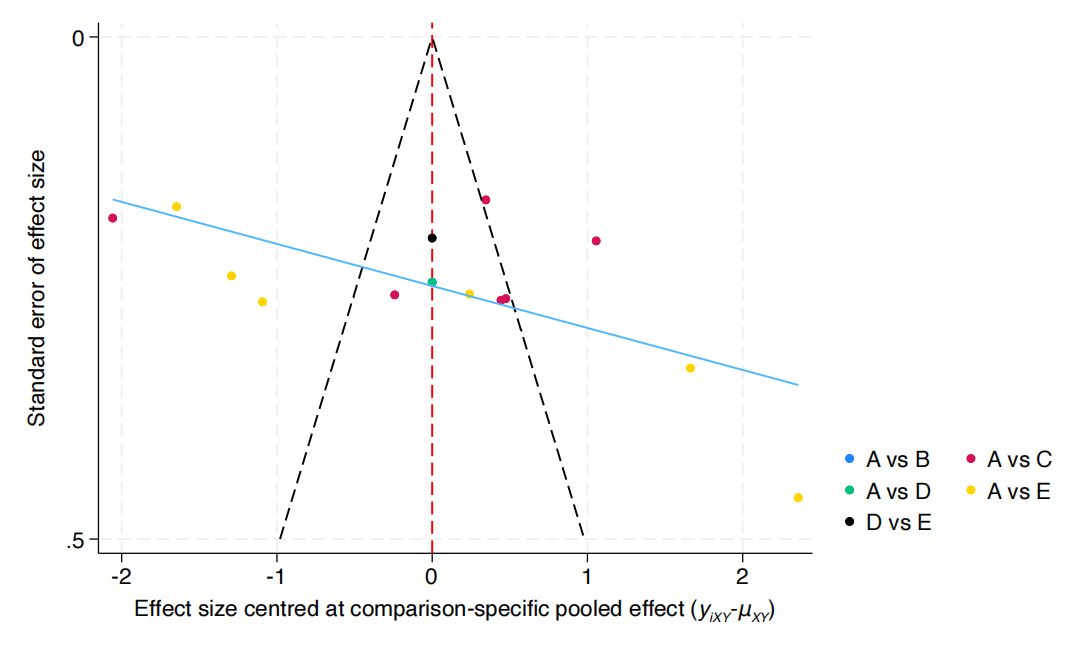


(5)TCM Syndrome Score:


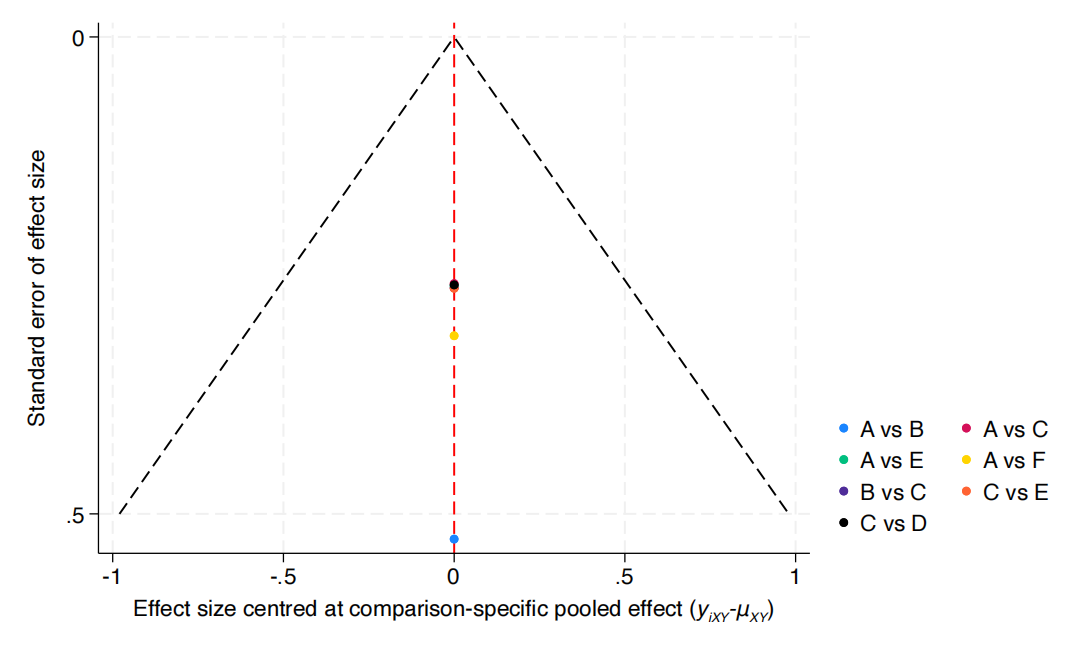


(6)Clinical Effective Rate:


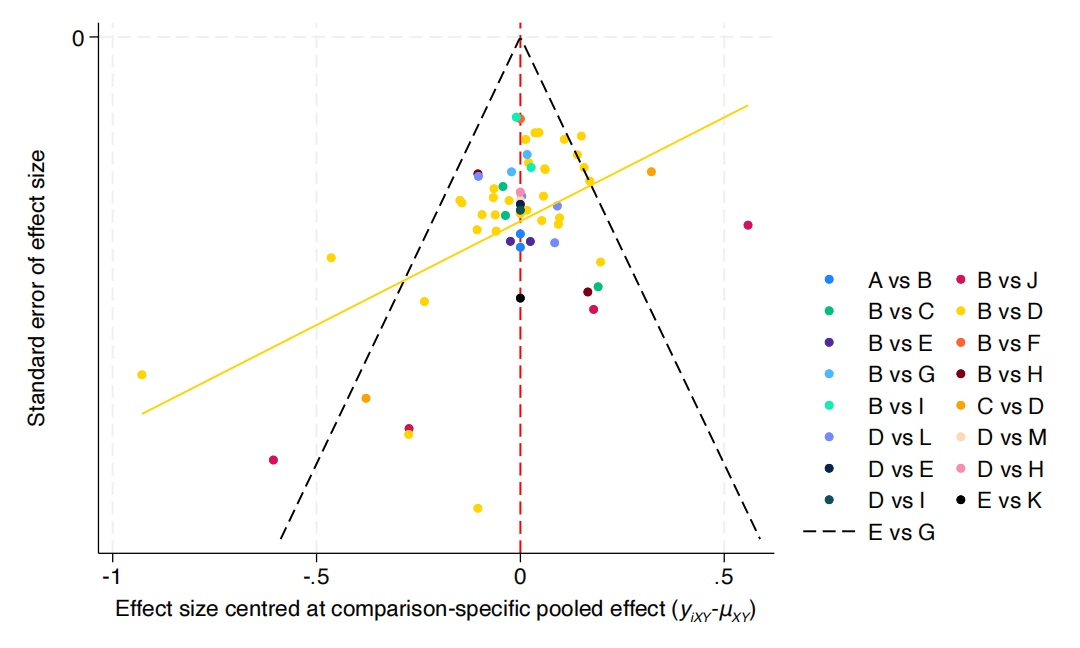


(7)Adverse Event Rate:


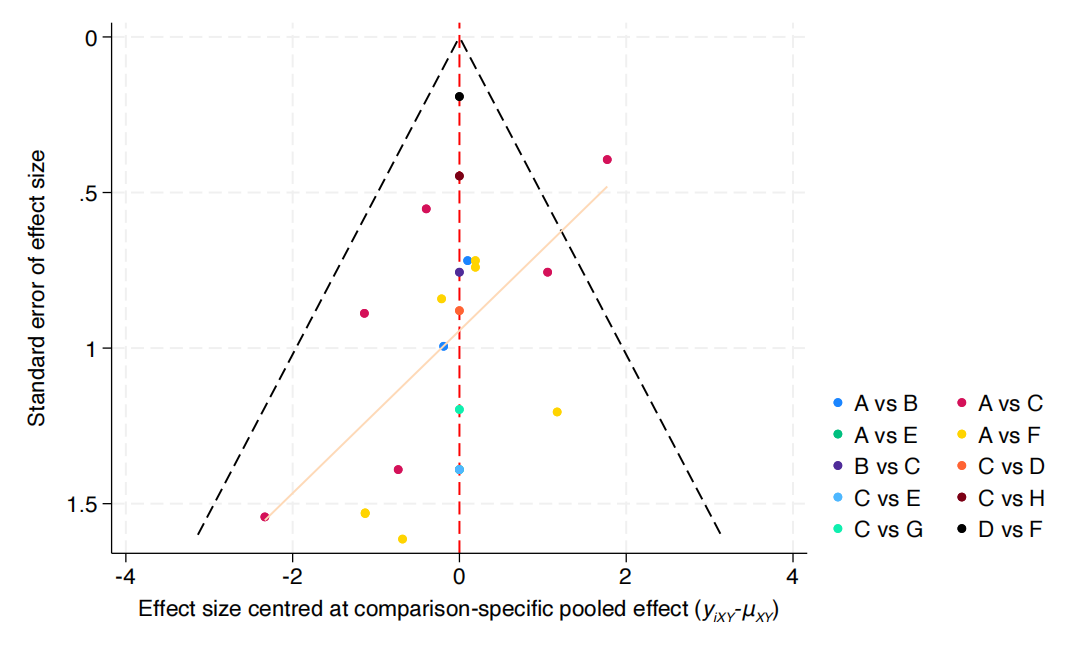

Supplement: Supplementary file 5 [file Table_5.DOCX]
