# Supplementary material for: Comparative effectiveness and safety of acupuncture treatments for primary insomnia: a systematic review and network meta-analysis of randomized trial
Source: Front Neurol. 2026 Mar 3;17:1750474. doi: 10.3389/fneur.2026.1750474 (PMC12992266; doi:10.3389/fneur.2026.1750474)

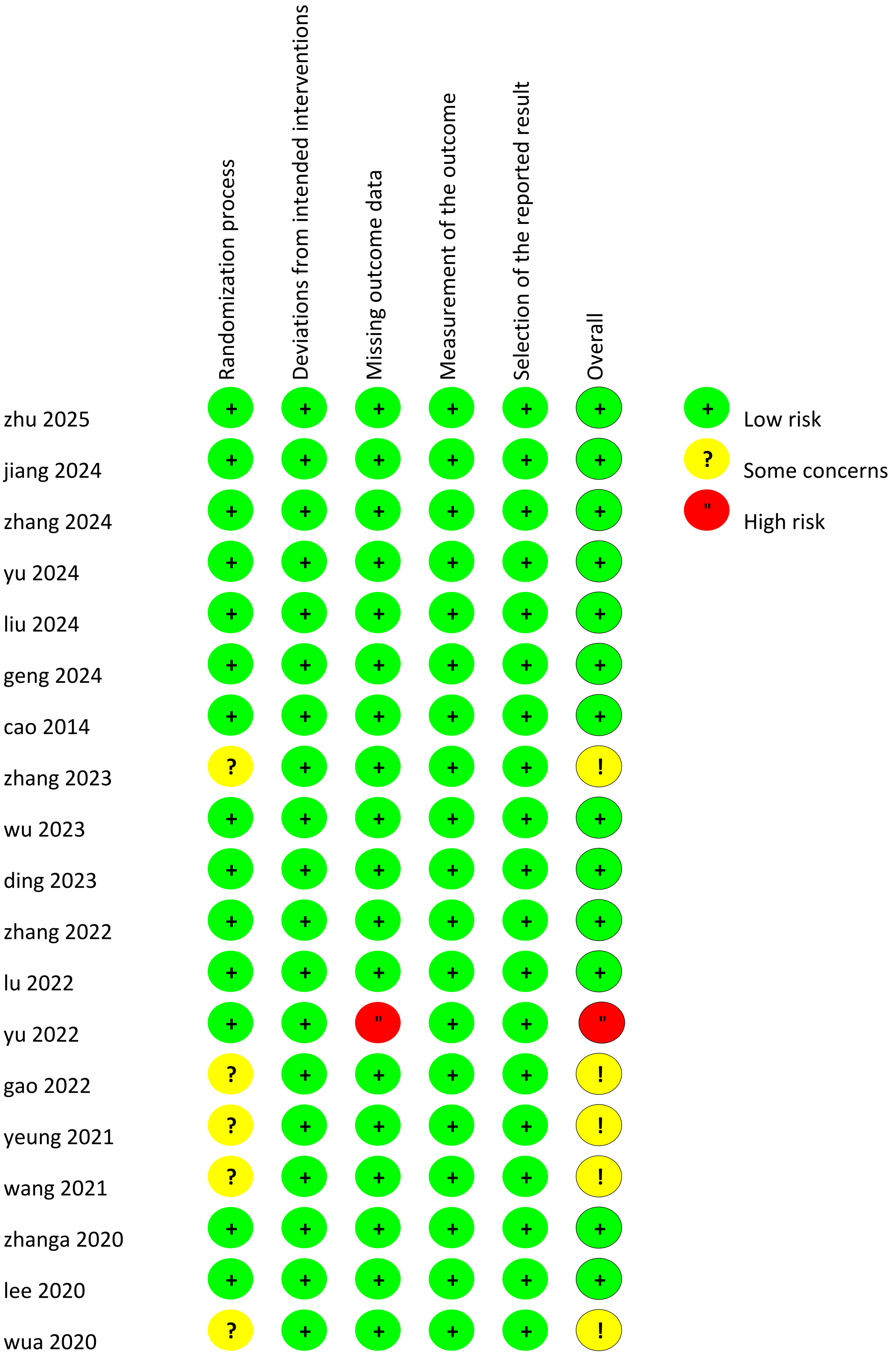

Randomization process
Deviations from intended interventions
Missing outcome data
Measurement of the outcome
Selection of the reported result
Overall
zhu 2025
jiang 2024
zhang 2024
yu 2024
liu 2024
geng 2024
cao 2014
zhang 2023
wu 2023
ding 2023
zhang 2022
lu 2022
yu 2022
gao 2022
yeung 2021
wang 2021
zhanga 2020
lee 2020
wua 2020
Low risk
Some concerns
High risk

zhangb 2020	+	+	+	+	+	+
liang 2020	+	+	+	+	+	+
wub 2020	+	+	+	+	+	+
sun 2020	+	+	+	+	+	+
li 2020	+	+	+	+	+	+
chen 2020	?	+	+	+	+	!
zou 2020	+	+	+	+	+	+
zhao 2019	+	+	+	+	+	+
xu 2019	+	+	"	+	+	"
guan 2019	+	+	+	+	+	+
zhu 2019	+	+	+	+	+	+
lia 2019	+	+	+	+	+	+
zhanga 2019	?	+	+	+	+	!
zhangb 2019	+	+	+	+	+	+
yuan 2019	+	+	+	+	+	+
wang 2019	+	+	+	+	+	+
guo 2019	?	+	"	+	+	"
lib 2019	+	+	+	+	+	+
qi 2019	?	+	+	+	+	!
zhao 2018	+	+	+	+	+	+
zhuo 2018	?	+	+	+	+	!
wan 2018	+	+	"	+	+	"
dong 2018	+	+	"	+	+	"
xie 2018	+	+	"	+	+	"
zhang 2018	?	+	"	+	+	"
cai 2018	+	+	+	+	+	+
hao 2018	+	+	+	+	+	+
kan 2018	+	+	+	+	+	+
zhou 2018	+	+	+	+	+	+
guo 2018	+	+	+	+	+	+

yin 2017	+	+	+	+	+	+
zhao 2017	+	+	+	+	+	+
wang 2017	?	+	+	+	+	!
han 2017	?	+	+	+	+	!
xie 2017	+	+	+	+	+	+
shao 2017	?	+	+	+	+	!
hong 2017	?	+	+	+	+	!
sun 2017	+	+	+	+	+	+
liu 2017	+	+	"	+	+	"
liang 2017	+	+	+	+	+	+
zhang 2017	+	+	+	+	+	+
wanga 2016	?	+	+	+	+	!
bo 2016	+	+	+	+	+	+
li 2016	+	+	+	+	+	+
hua 2016	?	+	+	+	+	!
gou 2016	?	+	"	+	+	"
wangb 2016	"	+	+	+	+	"
wangc 2016	+	+	"	+	+	"
luo 2016	?	+	+	+	+	!
wangd 2016	?	+	+	+	+	!
zhanga 2015	+	+	+	+	+	+
jia 2015	?	+	+	+	+	!
ding 2015	+	+	+	+	+	+
zou 2015	?	+	+	+	+	!
liua 2015	+	+	+	+	+	+
liub 2015	+	+	+	+	+	+
hong 2015	?	+	+	+	+	!
wang 2015	+	+	+	+	+	+
zhangb 2015	+	+	+	+	+	+
liuc 2015	?	+	+	+	+	!

jib 2015

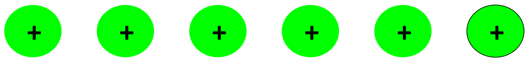

Supplement: Supplementary file 6 [file Data_Sheet_1.PDF]
